# Supplementary material for: Identifying monitoring information needs that support the management of fish in large rivers
Source: PLoS One. 2022 Apr 29;17(4):e0267113. doi: 10.1371/journal.pone.0267113 (PMC9053787; doi:10.1371/journal.pone.0267113)
Supplement: S1 Appendix — (DOCX) [file pone.0267113.s001.docx]

**Appendix S1.**

**South Canadian River**

The South Canadian River is the mainstem river of the Canadian River basin. The basin occupies a significant west-east climate gradient with precipitation ranging from 40 to 145 cm/yr [1]. Land use in the basin is primarily cropland or pasture that transitions to the urban area near Oklahoma City. Three major reservoirs on the South Canadian River have significantly altered the river flow patterns, particularly the frequency and magnitude of high-flow events, and the number of zero flow days (Fig S1). Multiyear to decadal droughts are not uncommon in the region [2], and groundwater pumping, particularly in the upper basin, has severed groundwater connections in many areas transforming the fish assemblage [3]. Further, groundwater pumping in the alluvial aquifer has decreased stream baseflows within this region [4, 5]. The rivers of the Great Plains are characterized by extreme physicochemical conditions (i.e., water temperatures 4 - 40°C; salinities greater than ocean water) and extensive flooding and extended periods of drought, and native fishes are well adapted to those extremes. Pelagic broadcast spawning minnows (i.e., pelagophils) belong to a reproductive guild of diminutive fishes that are emblematic of these stream systems. Of the approximately 20 species in this reproductive guild, 13 are of conservation concern; the status of the remaining seven species is poorly understood [6, 7].

**Colorado River**

The Colorado River flows 2,330 km from its origin in Colorado to its confluence with the Gulf of California in Mexico draining parts of seven U.S. and two Mexican states; our assessment focused on the portion of the Colorado River that flows through the Grand Canyon in Arizona (Fig 1). Water storage associated with the allocation of water in the Colorado River for human consumption and hydroelectric power generation has altered the hydrologic regime (Fig S2) in the Colorado River between Glen Canyon Dam and Lake Mead. Most fish species native to the Colorado River have declined in abundance and distribution while numerous non-native species have become established [8].

**Columbia River**

The Columbia River is the fourth largest river by volume in the United States [9] and drains a basin of 671,000 km^2^ that includes parts of seven states, land ceded to 14 groups of affiliated tribes in the U.S. portion of the Columbia River Basin, land ceded to three tribal groups, known as First Nations, in the Canadian portion of the basin, and one Canadian province ([10]; Fig 1). The Columbia River supports anadromous fish species such as Chinook (*Oncorhynchus tshawytscha)*, Coho (*O.* *kisutch)*, Chum (*O. keta)*, and Sockeye Salmon (*O. nerka)*, including 12 populations of four species of salmon and steelhead (*O. mykiss)* listed as threatened or endangered under the ESA. The Columbia River also contains a complement of resident native and non-native fishes.

**Upper Mississippi and Illinois Rivers**

The Upper Mississippi River begins at Lake Itasca in northern Minnesota and flows approximately 2300 km along the boundaries of five midwestern states, traversing 8 degrees of latitude before converging with the Ohio River (and subsequently referred to the Lower Mississippi River) [11]. The Illinois River is a significant tributary to the Upper Mississippi River and encompasses 439 km. Despite substantial alterations to both the channel environment and the basin, these two rivers support approximately 145 species of warmwater native fishes (and 8 nonnative species), including robust recreational and commercial stocks [12, 13].

**References**

1. Woods AJ, Omernik JM, Butler DR, Ford JG, Henley JE, Hoagland BW, et al. Ecoregions of Oklahoma (color poster with map, descriptive text, summary tables, and photographs). Reston, Virginia: U.S. Geological Survey; 2005.

2. Smith SJ, Ellis JH, Wagner DL, Peterson SM. Hydrogeology and simulated groundwater flow and availability in the North Fork Red River aquifer, southwest Oklahoma, 1980–2013. U.S. Geological Survey Scientific Investigations Report 2017–5098; 2017. p. 107.

3. Perkin JS, Gido KB, Falke JA, Fausch KD, Crockett H, Johnson ER, et al. Groundwater declines are linked to changes in Great Plains stream fish assemblages. P Natl Acad Sci USA. 2017;114(28):7373-8. doi: 10.1073/pnas.1618936114. PubMed PMID: WOS:000405177100073.

4. Fox GA. Evaluation of a stream aquifer analysis test using analytical solutions and field data. J Am Water Resour As. 2004;40(3):755-63. doi: DOI 10.1111/j.1752-1688.2004.tb04457.x. PubMed PMID: WOS:000222484600015.

5. Fox GA, Heeren DM, Miller RB, Mittelstet AR, Storm DE. Flow and transport experiments for a streambank seep originating from a preferential flow pathway. Journal of Hydrology. 2011;403(3-4):360-6. doi: 10.1016/j.jhydrol.2011.04.014. PubMed PMID: WOS:000291914200014.

6. Warren ML, Burr BM, Walsh SJ, Bart HL, Cashner RC, Etnier DA, et al. Diversity, distribution, and conservation status of the native freshwater fishes of the southern United States. Fisheries. 2000;25(10):7-31. doi: Doi 10.1577/1548-8446(2000)025<0007:Ddacso>2.0.Co;2. PubMed PMID: WOS:000089569600002.

7. Worthington TA, Echelle AA, Perkin JS, Mollenhauer R, Farless N, Dyer JJ, et al. The emblematic minnows of the North American Great Plains: A synthesis of threats and conservation opportunities. Fish and Fisheries. 2018;19(2):271-307. doi: 10.1111/faf.12254. PubMed PMID: WOS:000426503000006.

8. Olden JD, Poff NL, Bestgen KR. Life-history strategies predict fish invasions and extirpations in the Colorado River Basin. Ecol Monogr. 2006;76(1):25-40. PubMed PMID: WOS:000236562100003.

9. Kammerer JC. Largest rivers in the United States: U.S. Geological Survey Open-File Report OFR-87-242. 1987. p. 2.

10. Ebel WJ, Becker CD, Mullan JW, Raymond HL. The Columbia River: toward a holistic understanding. Canadian special publication of fisheries and aquatic sciences. 1989;106:205-19.

11. Delong MD. Upper Mississippi River Basin. In: Benke AC, Cushing CE, editors. Rivers of North America. 1. Burlington, MA: Elsevier Academic Press; 2005.

12. Schramm HL, Hatch JT, Hrabik RA, Slack WT. Fishes of the Mississippi River. In: Chen Y, Chapman D, Jackson J, Chen D, Zhongjie L, Killgore J, et al., editors. Fishery resources, environment, and conservation in the Mississippi and Yangtze (Changjiang) River basins. 84. Bethesda, MD: American Fisheries Society; 2016. p. 53-77.

13. Schramm HL, Ickes BS. The Mississippi River: A place for fish. In: Chen Y, Chapman D, Jackson J, Chen D, Zhongjie L, Killgore J, et al., editors. Fishery resources, environment, and conservation in the Mississippi and Yangtze (Changjiang) River basins. 84. Bethesda, MD: American Fisheries Society; 2016. p. 3-34.
