# Supplementary material for: Identifying monitoring information needs that support the management of fish in large rivers
Source: PLoS One. 2022 Apr 29;17(4):e0267113. doi: 10.1371/journal.pone.0267113 (PMC9053787; doi:10.1371/journal.pone.0267113)

Fig S1. Daily discharge (m^3^/s) patterns from 1938 to 2019 in the Canadian River, Oklahoma near Canadian, TX (data are available at <https://waterdata.usgs.gov/nwis/uv/?site_no=07228000>).


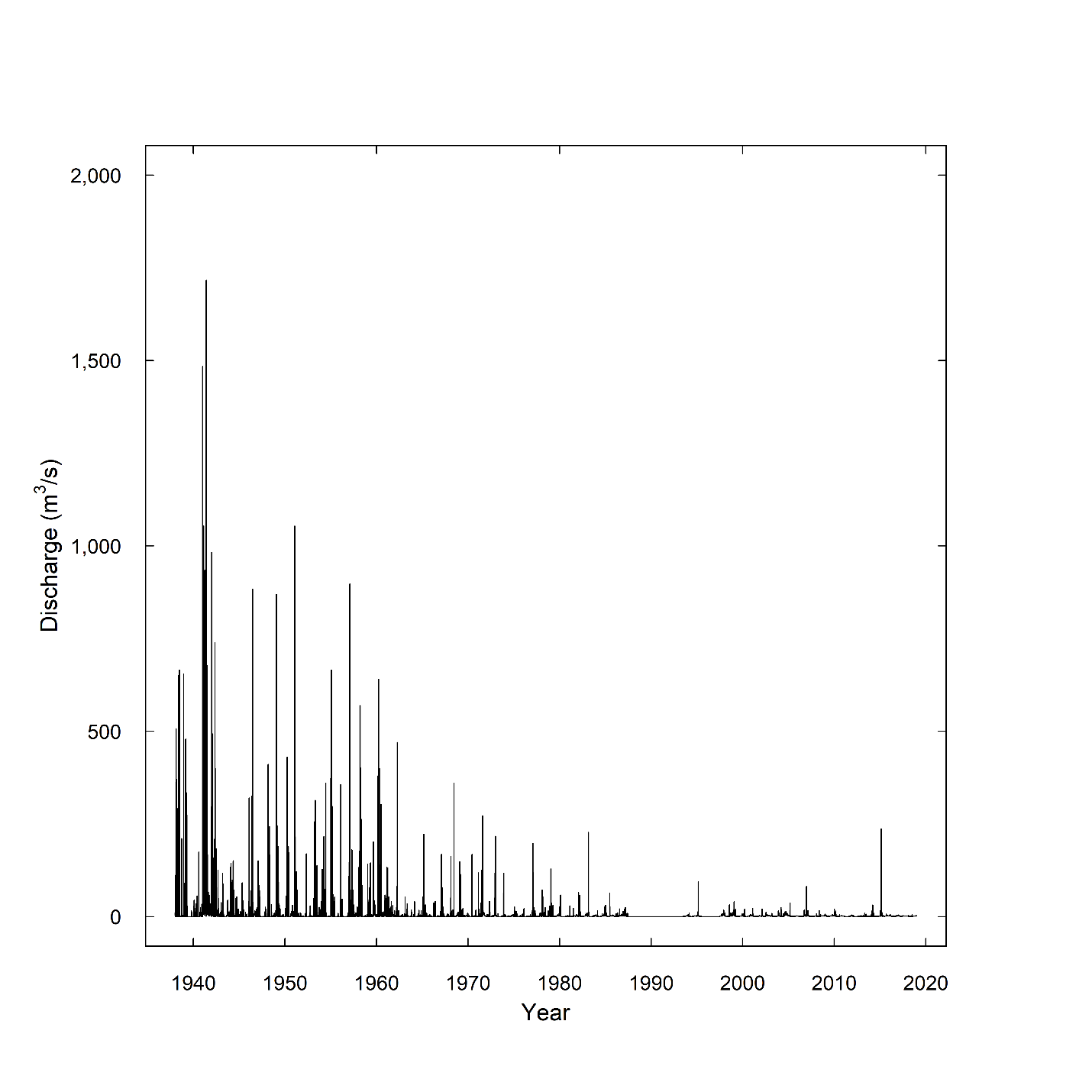

Supplement: S1 Fig — (DOCX) [file pone.0267113.s002.docx]
