# Supplementary material for: Identifying monitoring information needs that support the management of fish in large rivers
Source: PLoS One. 2022 Apr 29;17(4):e0267113. doi: 10.1371/journal.pone.0267113 (PMC9053787; doi:10.1371/journal.pone.0267113)
Supplement: S2 Fig — (DOCX) [file pone.0267113.s003.docx]

Fig S2. Hydrograph of the Colorado River at Lees Ferry, AZ from 1930 to 2019 showing pre- and post-Glen Canyon Dam closure in 1964 (dashed line) mean monthly discharge (m^3^/s), which transitions from seasonally stochastic to a more homogeneous regime focusing on anthropogenic interests (data are available at <https://waterdata.usgs.gov/nwis/dv/?site_no=09380000>).
