# Supplementary material for: Identifying monitoring information needs that support the management of fish in large rivers
Source: PLoS One. 2022 Apr 29;17(4):e0267113. doi: 10.1371/journal.pone.0267113 (PMC9053787; doi:10.1371/journal.pone.0267113)
Supplement: S3 Fig — A:Tier 2 EEC = larval Humpback Chub habitat; Stressors = habitat fragmentation, turbidity and Tier 2 EEC = Humpback Chub spawning habitat; Stressor = water temperature and Tier 3 EEC = insect production; Stressor = benthic macroinvertebrate habitat quantity and quality and Tier 3 EECs = all; Inter-tier interaction = trophic level interactions; B:Tier 3 EEC = larval Humpback Chub production; Stressor = larval Humpback chub habitat quantity and quality; C: Tier 3 EEC = Humpback Chub egg quality and production; Stressors = Humpback Chub spawning habitat quantity and quality; D:Larval Humpback Chub production; Inter-tier interaction = mortality of Humpback Chub eggs; E:Tier 3 EEC = larval Humpback Chub production; Stressor = predation by invasive species and Tier 3 EEC = Primary production; Stressor = nutrient flux; F:Tier 1 EEC = biogeochemistry/thermodynamics; Inter-tier interaction = sediment adsorption of contaminants and nutrients; G:Tier 3 EEC = Humpback Chub age-0 recruitment; Inter-tier interaction = mortality of larval Humpback Chub. (DOCX) [file pone.0267113.s004.docx]

Fig S3. The spatial and temporal scales of the management goal, the scientific inferences needed to inform the management goal, and that data collection needs to occur to support the inferences for monitoring information needs identified as requiring additional data in the case study addressing Humpback Chub recruitment in the Colorado River between Glen Canyon Dam and Lake Mead, Arizona (see Table S2 for additional detail). A:Tier 2 EEC= larval Humpback Chub habitat; Stressors=habitat fragmentation, turbidity and Tier 2 EEC= Humpback Chub spawning habitat; Stressor= water temperature and Tier 3 EEC=insect production; Stressor=benthic macroinvertebrate habitat quantity and quality and Tier 3 EECs=all; Inter-tier interaction=trophic level interactions; B:Tier 3 EEC=Humpback Chub larvae production; Stressor= larval Humpback chub habitat quantity and quality; C: Tier 3 EEC=Humpback Chub egg quality and production; Stressors= Humpback Chub spawning habitat quantity and quality; D:Humpback Chub larvae production; Inter-tier interaction=mortality of Humpback Chub eggs; E:Tier 3 EEC= Humpback Chub larvae production; Stressor=predation by invasive species and Tier 3 EEC=primary production; Stressor=nutrient flux; F:Tier 1 EEC= biogeochemistry/thermodynamics; Inter-tier interaction=sediment adsorption of contaminants and nutrients; G:Tier 3 EEC=Humpback Chub age-0 recruitment; Inter-tier interaction=mortality of Humpback Chub larvae.
