# Supplementary material for: Identifying monitoring information needs that support the management of fish in large rivers
Source: PLoS One. 2022 Apr 29;17(4):e0267113. doi: 10.1371/journal.pone.0267113 (PMC9053787; doi:10.1371/journal.pone.0267113)
Supplement: S4 Fig — (DOCX) [file pone.0267113.s005.docx]

Fig S4. Proportion of total annual Columbia River discharge at The Dalles, OR occurring in the month of June from 1879 to 2015 (data are available at <https://waterdata.usgs.gov/nwis/dv/?site_no=14105700>).
