# Supplementary material for: Identifying monitoring information needs that support the management of fish in large rivers
Source: PLoS One. 2022 Apr 29;17(4):e0267113. doi: 10.1371/journal.pone.0267113 (PMC9053787; doi:10.1371/journal.pone.0267113)
Supplement: S5 Fig — A:Tier 1 EEC = biogeochemistry/thermodynamic; Stressor = altered biogeochemical regime and Tier 2 EEC = benthic macroinvertebrate habitat; Stressors = channel stability, sediment deposition, fragmentation and Tier 2 EEC = Larval White Sturgeon habitat; Stressors = habitat fragmentation sediment deposition, water temperature and Tier 2 EEC = White Sturgeon spawning habitat; Stressors = contaminants, sediment deposition and Tier 3 EEC = White Sturgeon egg quality and production; Stressor = predation by invasive species and Tier 3 EEC = White Sturgeon larvae production; Stressor = predation by invasive species and Tier 3 EEC = benthic macroinvertebrate production; Stressor = benthic macroinvertebrate habitat quantity and quality; B:Tier 1 EEC = sediment transport; Stressors = altered sediment regime, altered hydraulic regime and Tier 1 EECs = channel morphology/hydraulics, sediment transport; Stressor = altered hydraulic regime and Tier 3 EEC = primary production; Stressor = nutrient fluxes; C:Tier 1 EEC = channel morphology/hydraulics, sediment transport; Stressor = altered hydraulic regime and Tier 1 EEC = channel morphology/hydraulics, sediment transport; Inter-tier interaction = sediment transport dynamics; D:Tier 1 EEC = channel morphology/hydraulics, sediment transport; Stressor = altered hydraulic regime and Tier 1 EEC = channel morphology/hydraulics, sediment transport; Inter-tier interaction = sediment transport dynamics; E:Tier 1 EEC = biogeochemistry/thermodynamics; Inter-tier interaction = sediment adsorption of contaminants and nutrients; F: Tier 3 EEC = White Sturgeon larvae production; Stressors = larval White Sturgeon habitat quantity and quality and Tier 3 EEC = White Sturgeon larvae production, age-0 White Sturgeon recruitment; Inter-tier interaction = mortality and White Sturgeon egg quality and production; Inter-tier interaction = predation of White Sturgeon eggs by native fish and Tier 3 EECs = White Sturgeon larvae production; Inter-tier interacti [file pone.0267113.s006.docx]

Fig S5. The spatial and temporal scales of the management goal, the scientific inferences needed to inform the management goal, and that data collection needs to occur to support the inferences for monitoring information needs identified as requiring additional data in the case study addressing White Sturgeon recruitment in the Columbia River (see Table S3 for additional detail). A:Tier 1 EEC= biogeochemistry/thermodynamic; Stressor= altered biogeochemical regime and Tier 2 EEC= benthic macroinvertebrate habitat; Stressors=channel stability, sediment deposition, fragmentation and Tier 2 EEC=larval White Sturgeon habitat; Stressors=habitat fragmentation sediment deposition, water temperature and Tier 2 EEC=White Sturgeon spawning habitat; Stressors=contaminants, sediment deposition and Tier 3 EEC=White Sturgeon egg quality and production; Stressor=predation by invasive species and Tier 3 EEC=White Sturgeon larvae production; Stressor=predation by invasive species and Tier 3 EEC=benthic macroinvertebrate production; Stressor=benthic macroinvertebrate habitat quantity and quality; B:Tier 1 EEC=sediment transport; Stressors=altered sediment regime, altered hydraulic regime and Tier 1 EECs=channel morphology/hydraulics, sediment transport; Stressor=altered hydraulic regime and Tier 3 EEC=primary production; Stressor=nutrient fluxes; C:Tier 1 EEC=channel morphology/hydraulics, sediment transport; Stressor=altered hydraulic regime and Tier 1 EEC = channel morphology/hydraulics, sediment transport; Inter-tier interaction=sediment transport dynamics; D:Tier 1 EEC=channel morphology/hydraulics, sediment transport; Stressor=altered hydraulic regime and Tier 1 EEC=channel morphology/hydraulics, sediment transport; Inter-tier interaction=sediment transport dynamics; E:Tier 1 EEC=biogeochemistry/thermodynamics; Inter-tier interaction=sediment adsorption of contaminants and nutrients; F: Tier 3 EEC=White Sturgeon larvae production; Stressors=larval White Sturgeon habitat quantity and quality and Tier 3 EEC=White Sturgeon larvae production, White Sturgeon age-0 recruitment; Inter-tier interaction=mortality and White Sturgeon egg quality and production; Inter-tier interaction=predation of White Sturgeon eggs by native fish and Tier 3 EECs=White Sturgeon larvae production; Inter-tier interaction=predation of White Sturgeon larvae by native fish and Tier 3 EECs=all; Inter-tier interactions=trophic level interactions.
