# Supplementary material for: Identifying monitoring information needs that support the management of fish in large rivers
Source: PLoS One. 2022 Apr 29;17(4):e0267113. doi: 10.1371/journal.pone.0267113 (PMC9053787; doi:10.1371/journal.pone.0267113)

Fig S6. Largemouth bass (*Micropterus salmoides*) data from the Pool 13 of the Upper Mississippi River (A, B) and the La Grange Pool of the Illinois River (C, D). The two river reaches are roughly the same latitude, but the La Grange Pool is more limited in overwintering habitat. Population abundance is presented in panels A and C where each point is an individual fish cumulatively caught with standardized day time electrofishing annually from 1993 to 2017 (data are available at <https://umesc.usgs.gov/data_library/fisheries/fish1_query.shtml>). The dashed triangle highlights ‘missing’ >400 mm size classes since 2000 in the La Grange Pool. Population size structure is indexed by proportional stock density (PSD) is presented in panels B and D with the dashed line showing trends in the largest size classes from 1993 to 2017 (data are available at <https://umesc.usgs.gov/data_library/fisheries/graphical/fish_front.html>).


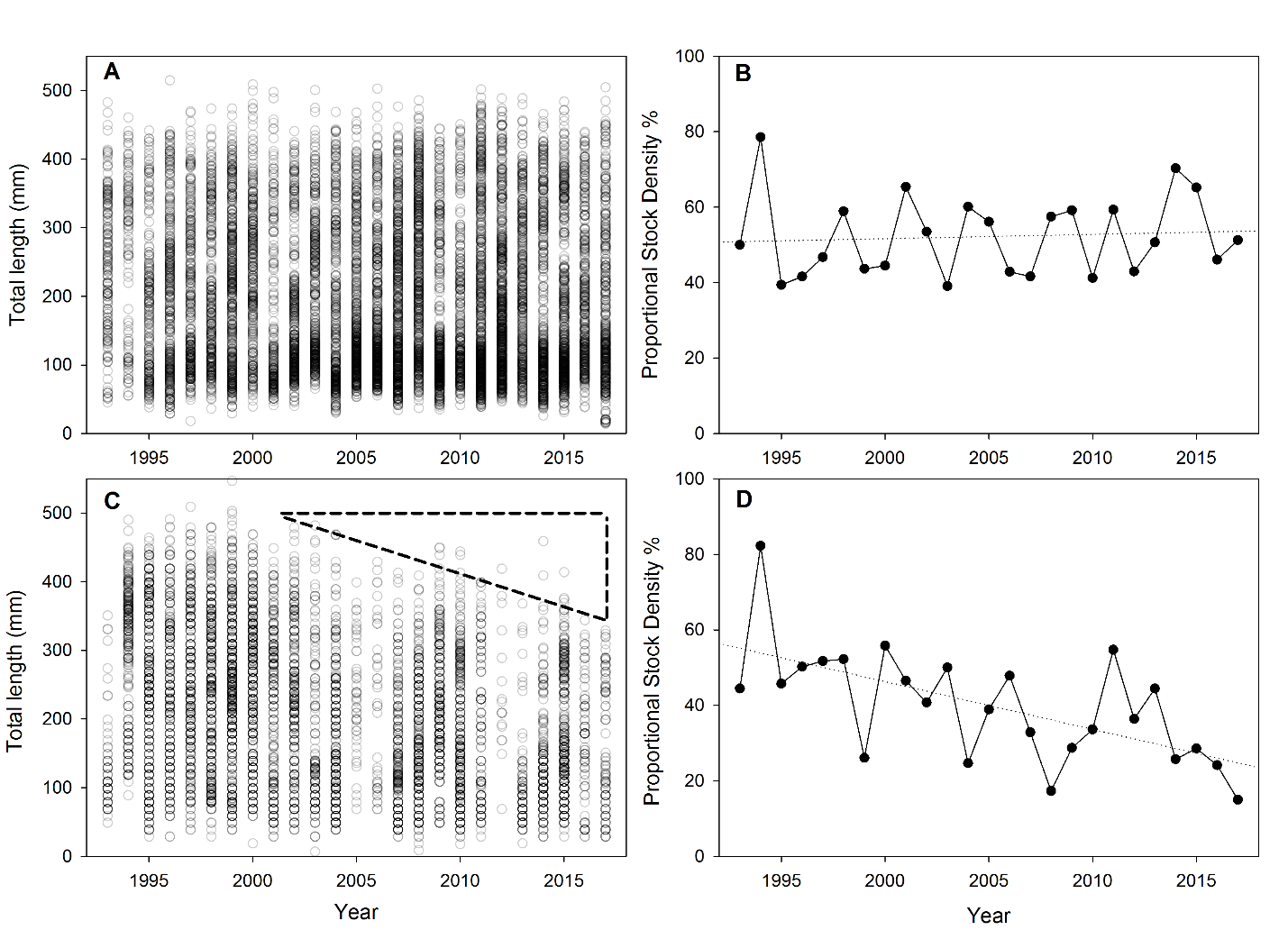

Supplement: S6 Fig — The two river reaches are roughly the same latitude, but the La Grange Pool is more limited in overwintering habitat. Population abundance is presented in panels A and C where each point is an individual fish cumulatively caught with standardized day time electrofishing annually from 1993 to 2017 (data available at https://umesc.usgs.gov/data_library/fisheries/fish1_query.shtml). The dashed triangle highlights ‘missing’ >400 mm size classes since 2000 in the La Grange Pool. Population size structure is indexed by proportional stock density (PSD) is presented in panels B and D with the dashed line showing trends in the largest size classes from 1993 to 2017 (data available at https://umesc.usgs.gov/data_library/fisheries/graphical/fish_front.html). (DOCX) [file pone.0267113.s007.docx]
