# Supplementary material for: Identifying monitoring information needs that support the management of fish in large rivers
Source: PLoS One. 2022 Apr 29;17(4):e0267113. doi: 10.1371/journal.pone.0267113 (PMC9053787; doi:10.1371/journal.pone.0267113)

Fig S7. The spatial and temporal scales of the management goal, the scientific inferences needed to inform the management goal, and that data collection needs to occur to support the inferences for monitoring information needs identified as requiring additional data in the case study addressing native fish biodiversity and habitat diversity in the Mississippi and Illinois rivers (see Table S4 for additional detail). A:Tier 1 EEC=sediment transport; Stressor=altered hydraulic regime and Tier 1 EEC=biogeochemistry/thermodynamics; Stressor=altered biogeochemical regime and Tier 1 EEC=biogeochemistry/thermodynamics; Inter-tier interaction=sediment adsorption of contaminants and nutrients; B: Tier 2 EEC=adult native fish overwintering habitat; Stressors=water velocity, water temperature, dissolved oxygen, sediment deposition and Tier 2 EEC=juvenile native fish habitat; Stressors=water depth, water velocity, water temperature, dissolved oxygen, contaminants, sediment deposition and Tier 2 EEC=native fish spawning habitat; Stressors=water depth, water velocity, habitat fragmentation, sediment deposition, water temperature, dissolved oxygen, contaminants and Tier 3 EEC=adult and juvenile native fish recruitment; Inter-tier interaction=mortality and Tier 3 EEC=all; Stressors=invasive species and Tier 3 EEC=all; Inter-tier interaction=trophic level interactions; C:Tier 1 EEC=channel morphology/hydraulics; Inter-tier interaction=channel forming processes; D:Tier 1 EEC=channel morphology/hydraulics, sediment transport; Inter-tier interaction=sediment transport dynamics; E:Tier 3 EEC=adult native fish recruitment; Stressor=adult native fish overwintering habitat quantity and quality and Tier 3 EEC=juvenile native fish recruitment; Stressor=juvenile native fish habitat quantity and quality and Tier 3 EEC=native fish egg quality and production; Stressor=spawning habitat quantity and quality.


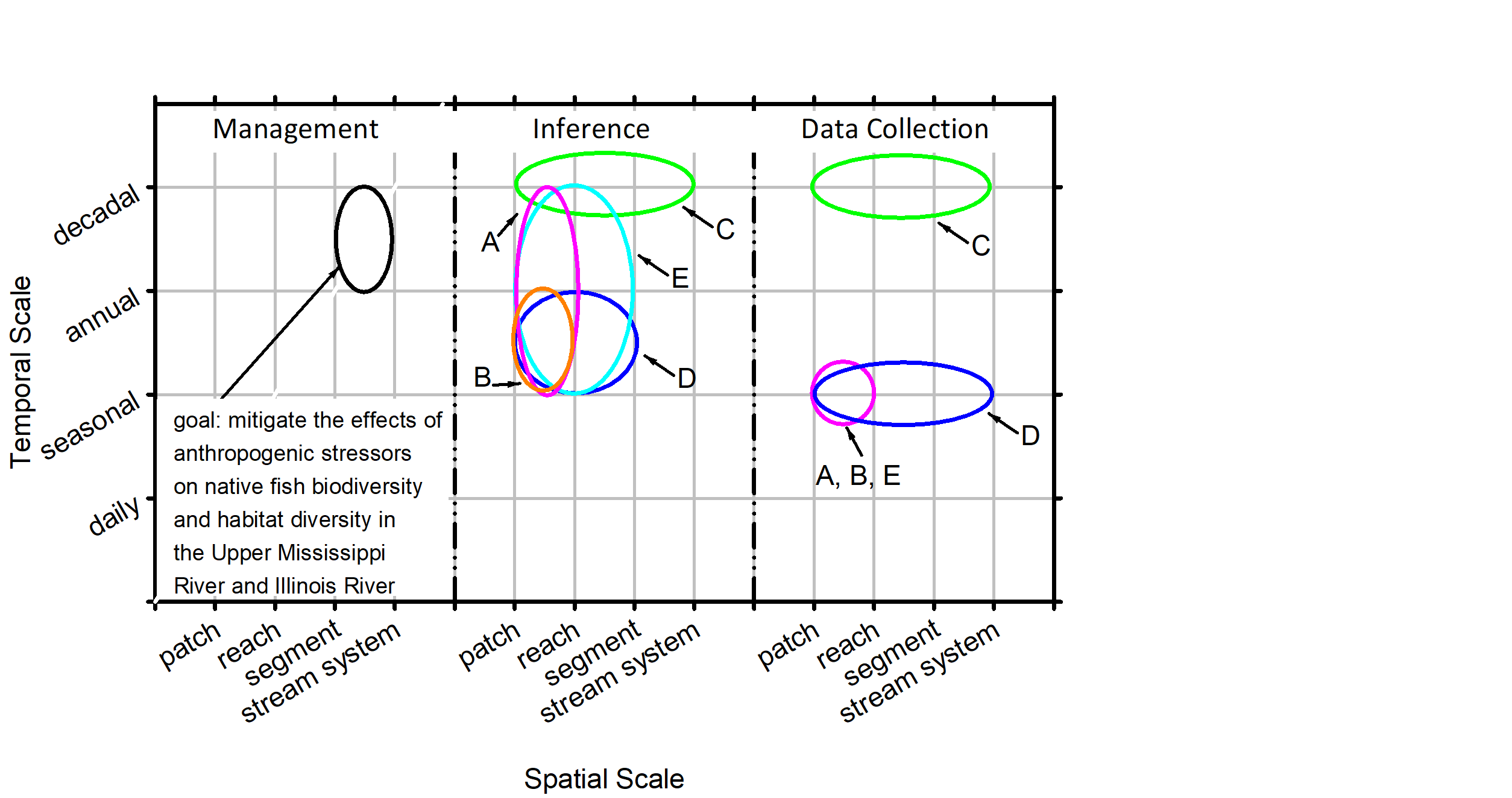

Supplement: S7 Fig — A:Tier 1 EEC = sediment transport; Stressor = altered hydraulic regime and Tier 1 EEC = biogeochemistry/thermodynamics; Stressor = altered biogeochemical regime and Tier 1 EEC = biogeochemistry/thermodynamics; Inter-tier interaction = sediment adsorption of contaminants and nutrients; B: Tier 2 EEC = adult native fish overwintering habitat; Stressors = water velocity, water temperature, dissolved oxygen, sediment deposition and Tier 2 EEC = juvenile native fish habitat; Stressors = water depth, water velocity, water temperature, dissolved oxygen, contaminants, sediment deposition and Tier 2 EEC = native fish spawning habitat; Stressors = water depth, water velocity, habitat fragmentation, sediment deposition, water temperature, dissolved oxygen, contaminants and Tier 3 EEC = adult and juvenile native fish recruitment; Inter-tier interaction = mortality and Tier 3 EEC = all; Stressors = invasive species and Tier 3 EEC = all; Inter-tier interaction = trophic level interactions; C:Tier 1 EEC = channel morphology/hydraulics; Inter-tier interaction = channel forming processes; D:Tier 1 EEC = channel morphology/hydraulics, sediment transport; Inter-tier interaction = sediment transport dynamics; E:Tier 3 EEC = adult native fish recruitment; Stressor = adult native fish overwintering habitat quantity and quality and Tier 3 EEC = juvenile native fish recruitment; Stressor = juvenile native fish habitat quantity and quality and Tier 3 EEC = native fish egg quality and production; Stressor = spawning habitat quantity and quality. (DOCX) [file pone.0267113.s008.docx]
