# Supplementary material for: Identifying monitoring information needs that support the management of fish in large rivers
Source: PLoS One. 2022 Apr 29;17(4):e0267113. doi: 10.1371/journal.pone.0267113 (PMC9053787; doi:10.1371/journal.pone.0267113)
Supplement: S1 Table — (DOCX) [file pone.0267113.s009.docx]

Table S1. Summary of information needs identified in the Conceptual Model describing factors affecting the recruitment of the Arkansas River Shiner in the South Canadian River, OK (Fig 4; this publication) by Essential Ecosystem Characteristic (EEC) Tier, EEC, and stressor or within Tier interactions and an assessment of the status of existing information that could be used to address the information needs.

| EEC Tier | EEC | Stressor or inter-tier interaction | Information need | Status of existing information |
| --- | --- | --- | --- | --- |
| 1 | Hydrology, Channel morphology/Hydraulics, Sediment transport | Altered hydrologic regime | Discharge | Insufficient |
| 1 | Channel morphology/Hydraulics | Altered hydraulic regime | Channel morphology characteristics and hydrodynamics in river segments with and without salt cedar | Insufficient |
| 1 | Biogeochemistry/Thermodynamics | Altered water temperature regime | Water temperatures | Insufficient |
| 1 | Biogeochemistry/Thermodynamics | Altered biogeochemical regimes | Contaminant and nutrient concentrations | Not available |
| 1 | Channel morphology/Hydraulics | Channel forming processes | Bathymetric change | Available |
| 1 | Channel morphology/Hydraulics, Sediment transport | Sediment transport dynamics | Bathymetric change, hydrodynamic model | Available |
| 1 | Biogeochemistry/Thermodynamics | Sediment adsorption of contaminants and nutrients | Estimates of contaminant and nutrient concentrations, turbidity, sediment composition | Not available |
| 2 | Arkansas River Shiner spawning habitat | Habitat fragmentation, contaminants, water temperature | Relation of contaminants and water temperature to Arkansas River Shiner spawning habitat, geospatial accounting of contaminants, water temperature, and Arkansas River Shiner spawning habitat | Insufficient |
| 2 | Larval Arkansas River Shiner habitat | Habitat fragmentation, water temperature | Relation between water temperatures and Arkansas River Shiner larval development and abundance, geospatial accounting of water temperature and Arkansas River Shiner larval abundance | Insufficient |

Table S1 (Cont.). Summary of information needs identified in the Conceptual Model describing factors affecting the recruitment of the Arkansas River Shiner in the South Canadian River, OK (Fig. 4; this publication) by Essential Ecosystem Characteristic (EEC) Tier, EEC, and stressor or within Tier interactions and an assessment of the status of existing information that could be used to address the information needs.

| EEC Tier | EEC | Stressor or inter-tier interaction | Information need | Status of existing information |
| --- | --- | --- | --- | --- |
| 2 | Invertebrate habitat | Discharge, sediment deposition, altered riparian plant community | Relation of discharge and sedimentation patterns to riparian vegetation, relation of discharge and riparian vegetation to invertebrate abundance and drift, geospatial accounting of riparian vegetation and drift invertebrate abundance | Not available |
| 3 | Primary production | Nutrient flux | Nutrient concentrations | Not available |
| 3 | Invertebrate production | Invertebrate habitat quantity and quality | Indices of invertebrate habitat quality, geospatial accounting of invertebrate habitat | Not available |
| 3 | Arkansas River Shiner larvae production | Arkansas River Shiner larval habitat quantity and quality | Habitat classification and indices of quality, geospatial accounting of larval Arkansas River Shiner habitat | Insufficient |
| 3 | Arkansas River Shiner larvae production | Direct mortality from recreational use (ATV and in-river traffic) | Arkansas River Shiner mortality from recreational use | Not available |
| 3 | Arkansas River Shiner larvae production | Predation/competition by invasive species | Arkansas River Shiner predation/competition with invasive species | Not available |
| 3 | Arkansas River Shiner egg quality and production | Arkansas River Shiner spawning habitat quantity and quality | Habitat classification and indices of quality, geospatial accounting of Arkansas River Shiner spawning habitat | Insufficient |

Table S1 (Cont.). Summary of information needs identified in the Conceptual Model describing factors affecting the recruitment of the Arkansas River Shiner in the South Canadian River, OK (Fig. 4; this publication) by Essential Ecosystem Characteristic (EEC) Tier, EEC, and stressor or within Tier interactions and an assessment of the status of existing information that could be used to address the information needs.

| EEC Tier | EEC | Stressor or inter-tier interaction | Information need | Status of existing information |
| --- | --- | --- | --- | --- |
| 3 | Arkansas River Shiner egg quality and production | Arkansas River Shiner age 1+ fish condition | Age, length, and weight | Available |
| 3 | Arkansas River Shiner larvae production | Arkansas River Shiner egg mortality | Arkansas River Shiner egg mortality | Not available |
| 3 | Arkansas River Shiner age-0 recruitment | Arkansas River Shiner larvae mortality | Arkansas River Shiner larvae mortality | Insufficient |
| 3 | Arkansas River Shiner age-1+ recruitment | Arkansas River Shiner age-0 mortality | Arkansas River Shiner age-0 mortality | Insufficient |
| 3 | All | Trophic level interactions | Trophic level dynamics | Not available |
