# Supplementary material for: Identifying monitoring information needs that support the management of fish in large rivers
Source: PLoS One. 2022 Apr 29;17(4):e0267113. doi: 10.1371/journal.pone.0267113 (PMC9053787; doi:10.1371/journal.pone.0267113)
Supplement: S2 Table — (DOCX) [file pone.0267113.s010.docx]

Table S2. Summary of information needs identified in the Conceptual Model describing factors affecting the recruitment of the Humpback Chub in the Colorado River, Arizona (Fig 6; this publication) by Essential Ecosystem Characteristic (EEC) Tier, EEC, and stressor or inter-tier interactions and an assessment of the status of existing information that could be used to address the information needs.

| EEC Tier | EEC | Stressor or inter-tier interaction | Information need | Status of existing information |
| --- | --- | --- | --- | --- |
| 1 | Hydrology, Sediment transport | Altered hydrologic regime | Discharge | Available |
|  |  |  |  |  |
| 1 | Biogeochemistry/thermodynamics | Altered water temperature regime | Water temperature | Available |
| 1 | Channel morphology/Hydraulics | Channel forming processes | Bathymetric change | Available |
| 1 | Channel morphology/Hydraulics, Sediment transport | Sediment transport dynamics | Bathymetric change, hydrodynamic model | Available |
| 1 | Biogeochemistry/thermodynamics | Sediment adsorption of contaminants and nutrients | Turbidity, sediment composition | Available |
| 1 | Biogeochemistry/thermodynamics | Sediment adsorption of contaminants and nutrients | Estimates of contaminant and nutrient concentrations | Insufficient |
| 2 | Benthic macroinvertebrate habitat | Channel stability, habitat fragmentation, sediment deposition | Relation of channel stability and sediment deposition to benthic macroinvertebrate abundance, geospatial accounting of channel stability, sediment deposition, and benthic macroinvertebrate abundance | Available |
| 2 | Larval Humpback Chub habitat | Habitat fragmentation, turbidity | Relation of turbidity to Humpback Chub larval abundance, geospatial accounting of turbidity and Humpback Chub larval abundance | Insufficient |
| 2 | Humpback Chub spawning habitat | Water temperature | Relation of water temperature to Humpback Chub spawning habitat, geospatial accounting of water temperature | Insufficient |

Table S2 (cont.). Summary of information needs identified in the Conceptual Model describing factors affecting the recruitment of the Humpback Chub in the Colorado River, Arizona (Fig. 6; this publication) by Essential Ecosystem Characteristic (EEC) Tier, EEC, and stressor or inter-tier interactions and an assessment of the status of existing information that could be used to address the information needs.

| EEC Tier | EEC | Stressor or inter-tier interaction | Information need | Status of existing information |
| --- | --- | --- | --- | --- |
| 3 | Primary production | Nutrient flux | Nutrient concentrations | Insufficient |
| 3 | Insect production | Benthic macroinvertebrate habitat quantity and quality | Habitat classification and indices of quality, geospatial accounting of benthic macroinvertebrate habitat | Insufficient |
| 3 | Humpback Chub egg quality and production | Humpback Chub spawning habitat quantity and quality | Habitat classification and indices of quality, geospatial accounting of Humpback Chub spawning habitat | Insufficient |
| 3 | Humpback Chub larvae production | Larval Humpback chub habitat quantity and quality | Habitat classification and indices of quality, geospatial accounting of larval Humpback Chub habitat | Not available |
| 3 | Humpback Chub larvae production | Mortality of Humpback Chub eggs | Estimates of egg production and mortality rates | Not available |
| 3 | Humpback Chub larvae production | Predation by invasive species | Larval Humpback Chub predation rates | Insufficient |
| 3 | Humpback Chub age-0 recruitment | Mortality of larval Humpback Chub | Estimates of larval Humpback Chub production and mortality rates | Insufficient |
| 3 | All | Trophic level interactions | Trophic level dynamics | Insufficient |
