# Supplementary material for: Identifying monitoring information needs that support the management of fish in large rivers
Source: PLoS One. 2022 Apr 29;17(4):e0267113. doi: 10.1371/journal.pone.0267113 (PMC9053787; doi:10.1371/journal.pone.0267113)
Supplement: S3 Table — (DOCX) [file pone.0267113.s011.docx]

Table S3. Summary of information needs identified in the Conceptual Model describing factors affecting the recruitment of age-0 White Sturgeon in the Columbia River (Fig 7; this publication), by Essential Ecosystem Characteristic (EEC) Tier, EEC, and stressor or inter-tier interactions and an assessment of the status of existing information that could be used to address the information needs.

| EEC Tier | EEC | Stressor or inter-tier interaction | Information need | Status of existing information |
| --- | --- | --- | --- | --- |
| 1 | Hydrology | Altered hydrologic regime | Discharge | Available |
| 1 | Channel morphology/Hydraulics, Sediment transport | Altered hydraulic regime | Bathymetry, hydrodynamic model | Insufficient |
| 1 | Sediment transport | Altered sediment regime | Hydrodynamic model, substrate composition | Insufficient |
| 1 | Biogeochemistry/Thermodynamics | Altered water temperature regime | Water temperature | Available |
| 1 | Biogeochemistry/Thermodynamics | Altered biogeochemical regime | Contaminant and nutrient concentrations in sediment and water | Insufficient |
| 1 | Channel morphology/Hydraulics | Channel forming processes | Bathymetric change | Insufficient |
| 1 | Channel morphology/Hydraulics, Sediment transport | Sediment transport dynamics | Bathymetric change, hydrodynamic model, substrate composition | Insufficient |
| 1 | Biogeochemistry/Thermodynamics | Sediment adsorption of contaminants and nutrients | Estimates of contaminant and nutrient concentrations, turbidity, sediment composition | Insufficient |
| 2 | Benthic macroinvertebrate habitat | Channel stability, habitat fragmentation, sediment deposition | Relation of channel stability and sediment deposition to benthic macroinvertebrate abundance; geospatial accounting of channel stability, sediment deposition, and benthic macroinvertebrate abundance | Not available |
| 2 | Larval White Sturgeon habitat | Habitat fragmentation sediment deposition, water temperature | Relation of sediment deposition and water temperature to larval White Sturgeon abundance; geospatial accounting of sediment deposition and White Sturgeon larval abundance | Not available |

Table S3 (cont.). Summary of information needs identified in the Conceptual Model describing factors affecting the recruitment of age-0 White Sturgeon in the Columbia River (Fig. 7; this publication), by Essential Ecosystem Characteristic (EEC) Tier, EEC, and stressor or inter-tier interactions and an assessment of the status of existing information that could be used to address the information needs.

| EEC Tier | EEC | Stressor or inter-tier interaction | Information need | Status of existing information |
| --- | --- | --- | --- | --- |
| 2 | White Sturgeon spawning habitat | Water velocity, habitat fragmentation, water temperature | Relation of water temperature and water velocity to White Sturgeon spawning habitat quantity and quality | Available |
| 2 | White Sturgeon spawning habitat | Sediment deposition, contaminants | Sediment deposition in spawning areas, contaminant concentrations in sediment and water, | Not available |
| 3 | White Sturgeon egg quality and production | Predation by invasive species | White Sturgeon egg predation rates | Not available |
| 3 | White Sturgeon larvae production | Predation by invasive species | Larval White Sturgeon predation rates | Not available |
| 3 | Primary production | Nutrient fluxes | Nutrient concentrations, point source contributions of nutrients | Insufficient |
| 3 | Benthic macroinvertebrate production | Benthic macroinvertebrate habitat quantity and quality | Habitat classification and indices of quality, geospatial accounting of benthic macroinvertebrate habitat | Not available |
| 3 | White Sturgeon larvae production | Larval White Sturgeon habitat quantity and quality | Habitat classification and indices of quality, geospatial accounting of larval White Sturgeon habitat | Not available |
| 3 | White Sturgeon egg quality and production | White Sturgeon spawning habitat quantity and quality | Habitat classification and indices of quality, geospatial accounting of White Sturgeon spawning habitat | Available |
| 3 | White Sturgeon larvae production | Mortality of White Sturgeon eggs | White Sturgeon egg mortality rates | Not available |

Table S3 (cont.). Summary of information needs identified in the Conceptual Model describing factors affecting the recruitment of age-0 White Sturgeon in the Columbia River (Fig. 7; this publication), by Essential Ecosystem Characteristic (EEC) Tier, EEC, and stressor or inter-tier interactions and an assessment of the status of existing information that could be used to address the information needs.

| EEC Tier | EEC | Stressor or inter-tier interaction | Information need | Status of existing information |
| --- | --- | --- | --- | --- |
| 3 | White Sturgeon age-0 recruitment | Mortality of larval white sturgeon | Larval White Sturgeon mortality rates | Not available |
| 3 | White Sturgeon adult recruitment | Mortality of age-0 White Sturgeon | Age-0 White Sturgeon mortality rates | Insufficient |
| 3 | White Sturgeon egg quality and production | Adult fish condition | Adult fish condition | Available |
| 3 | White Sturgeon egg quality and production | Predation of White Sturgeon eggs by native fish | White Sturgeon eggs predation rates | Not available |
| 3 | White Sturgeon larvae production | Predation of White Sturgeon larvae by native fish | Larval White Sturgeon predation rates | Not available |
| 3 | All | Trophic level interactions | Trophic level dynamics | Not available |
