# Supplementary material for: Identifying monitoring information needs that support the management of fish in large rivers
Source: PLoS One. 2022 Apr 29;17(4):e0267113. doi: 10.1371/journal.pone.0267113 (PMC9053787; doi:10.1371/journal.pone.0267113)
Supplement: S4 Table — (DOCX) [file pone.0267113.s012.docx]

Table S4. Summary of information needs identified in the Conceptual Model describing factors affecting the restoration and maintenance of native fish biodiversity and habitat quantity and quality in the Upper Mississippi and Illinois rivers (Fig 8; this publication), by Essential Ecosystem Characteristic (EEC) Tier, EEC, and stressor or inter-tier interactions and an assessment of the status of existing information that could be used to address the information needs.

| EEC Tier | EEC | Stressor | Information need | Status of existing information |
| --- | --- | --- | --- | --- |
| 1 | Hydrology | Altered hydrologic regime | Discharge | Available |
| 1 | Channel morphology/ hydraulics | Altered hydraulic regime | Topographic and bathymetric information | Available |
| 1 | Sediment transport | Altered sediment regime | Sediment deposition, sediment transport | Insufficient |
| 1 | Biogeochemistry/thermodynamic | Altered biogeochemical regime | Dissolved oxygen, contaminant and nutrient concentrations | Insufficient |
| 1 | Biogeochemistry/thermodynamic | Altered water temperature regime | Water temperature | Available |
| 1 | Channel morphology/Hydraulics | Channel forming processes | Bathymetric change | Insufficient |
| 1 | Channel morphology/Hydraulics, Sediment transport | Sediment transport dynamics | Bathymetric change, hydrodynamic model | Insufficient |
| 1 | Biogeochemistry/thermodynamics | Sediment adsorption of contaminants and nutrients | Estimates of contaminant and nutrient concentrations, turbidity, sediment composition | Insufficient |
| 2 | Adult native fish overwintering habitat | Water velocity, water temperature, dissolved oxygen, sediment deposition | Relation of dissolved oxygen, sediment deposition, water temperature, and water velocity to adult native fish overwintering abundance; geospatial accounting of stressors affecting native fish overwintering quality | Insufficient |

Table S4 (cont). Summary of information needs identified in the Conceptual Model describing factors affecting the restoration and maintenance of native fish biodiversity and habitat quantity and quality in the Upper Mississippi and Illinois rivers (Fig. 8; this publication), by Essential Ecosystem Characteristic (EEC) Tier, EEC, and stressor or inter-tier interactions and an assessment of the status of existing information that could be used to address the information needs.

| EEC Tier | EEC | Stressor | Information need | Status of existing information |
| --- | --- | --- | --- | --- |
| 2 | Juvenile native fish habitat | Water depth, water velocity, water temperature, dissolved oxygen, contaminants, sediment deposition | Relation of dissolved oxygen, contaminants, sediment deposition, water depth, water temperature, and water velocity on juvenile abundance; geospatial accounting of stressors affecting juvenile native fish habitat quality | Not available |
| 2 | Native fish spawning habitat | Water depth, water velocity, habitat fragmentation, sediment deposition, water temperature, dissolved oxygen, contaminants | Relation of contaminants, dissolved oxygen, sediment deposition, water depth, water temperature, and water velocity to native fish spawning habitat quality and quantity, geospatial accounting of stressors that affect quality of native fish spawning habitat | Insufficient |
| 3 | Adult native fish recruitment | Adult native fish overwintering habitat quantity and quality | Habitat classification and indices of quality, geospatial accounting of adult native fish overwintering habitat | Insufficient |
| 3 | Juvenile native fish recruitment | Juvenile native fish habitat quantity and quality | Habitat classification and indices of quality, geospatial accounting of juvenile native fish habitat | Insufficient |
| 3 | Native fish egg quality and production | Spawning habitat quantity and quality | Habitat classification and indices of quality, geospatial accounting of native fish spawning habitat | Insufficient |
| 3 | Native fish egg quality and production | Adult native fish condition | Adult native fish condition | Available |

Table S4 (cont). Summary of information needs identified in the Conceptual Model describing factors affecting the restoration and maintenance of native fish biodiversity and habitat quantity and quality in the Upper Mississippi and Illinois rivers (Fig. 8; this publication), by Essential Ecosystem Characteristic (EEC) Tier, EEC, and stressor or inter-tier interactions and an assessment of the status of existing information that could be used to address the information needs.

| EEC Tier | EEC | Stressor | Information need | Status of existing information |
| --- | --- | --- | --- | --- |
| 3 | Adult and juvenile native fish recruitment | Mortality | Native fish egg mortality rates, larval native fish mortality rates, juvenile native fish mortality rates | Not available |
| 3 | All | Invasive species, trophic level interactions | Plankton community composition and production, trophic level dynamics | Insufficient |
