# Supplementary material for: Identifying monitoring information needs that support the management of fish in large rivers
Source: PLoS One. 2022 Apr 29;17(4):e0267113. doi: 10.1371/journal.pone.0267113 (PMC9053787; doi:10.1371/journal.pone.0267113)
Supplement: Attachment — Submitted filename: PONE-D-21-12444_R1_Rev_Oct2021_Fin.pdf [file pone.0267113.s014.pdf]

PLOS ONE

Identifying monitoring information needs that support the management of fish in large rivers

--Manuscript Draft--

**Manuscript Number:**	PONE-D-21-12444R1
**Article Type:**	Research Article
**Full Title:**	Identifying monitoring information needs that support the management of fish in large rivers
**Short Title:**	Identifying monitoring information needs that support the management of fish in large rivers
**Corresponding Author:**	Timothy D. Counihan, MSc<br>US Geological Survey<br>Cook, WA UNITED STATES
**Keywords:**	large river; monitoring; long-term; information needs; fish; conceptual model, ecosystem
**Abstract:**	Management actions intended to benefit fish in large rivers can directly or indirectly affect multiple ecosystem components. Without consideration of the effects of management on non-target ecosystem components, unintended consequences may limit management efficacy. Monitoring can help clarify the effects of management actions, including on non-target ecosystem components, but only if data are collected to characterize key ecosystem processes that could affect the outcome. Scientists from across the U.S. convened to develop a conceptual model that would help identify monitoring information needed to better understand how natural and anthropogenic factors affect large river fishes. We applied the conceptual model to case studies in four large U.S. rivers. The application of the conceptual model indicates the model is flexible and relevant to large rivers in different geographic settings and with different management challenges. By visualizing how natural and anthropogenic drivers directly or indirectly affect cascading ecosystem tiers, our model identified critical information gaps and uncertainties that, if resolved, could inform how to best meet management objectives. Despite large differences in the physical and ecological contexts of the river systems, the case studies also demonstrated substantial commonalities in the data needed to better understand how stressors affect fish in these systems. For example, in most systems information on river discharge and water temperature were needed and available. Conversely, information regarding trophic relationships and the habitat requirements of larval fishes were generally lacking. This result suggests that there may be a common need for a better understanding of certain factors across large-river systems.
**Order of Authors:**	Timothy D. Counihan, MSc
	Kristen L Bouska
	Shannon K Brewer
	Robert Jacobson
	Andrew F Casper
	Colin G Chapman
	Ian R Waite
	Kenneth R Sheehan
	Mark Pyron
	Elise R Irwin
	Karen Riva-Murray
	Alexa McKerrow
	Jennifer M Bayer

Powered by Editorial Manager® and ProduXion Manager® from Aries Systems Corporation

Response to Reviewers:	PONE-D-21-12444

PONE-D-21-12444
Identifying monitoring information needs that support the management of fish in large rivers
PLOS ONE

Reviewer 1 wonders about the utility of such conceptual models in the more realistic case of multiple species contexts. How could the model be expanded in this regard, besides the case of simple richness measures? In addition, how could the model help in actually prioritising or ranking the variables or interactions identified? Finally, reviewer 1 also noticed that an important stressor related to fragmentation and connectivity is only marginally discussed and included in the model. This is critical for meta-population dynamics and should be given more emphasis.

Reviewer 2, similarly raises the critical issue of the multi-species context, and how the needs of different species could be simultaneously identified. Therefore, I advice to expand the Discussion in this regard, eventually acknowledging limitations and suggesting future research needs. Reviewer 2 also wonders how such models could effectively guide restoration and decision-making beyond monitoring needs; if the relative importance of data gaps and interactions is not quantified (e.g. via a cost-benefit analysis), how could it help prioritise the focus of monitoring and action?

Besides carefully responding to each reviewers' comments and modify the manuscript accordingly, I also suggest to simplify the manuscript, which feels rather long. Perhaps some of the background information from each case study could be included as supplementary or shortened. Also, caption from Fig.5 (and sister-figures) is rather hard to digest for the reader. I wonder if this could be simplified as well.

Response: Thank you for the opportunity to revise our manuscript. We have tried to address comments of Reviewer 1 and 2 below and in the revised manuscript. To shorten and simplify the manuscript, we relocated some of the contextual information from the case studies and moved it to an Appendix in Supplemental Information. With respect to Fig 5 and sister figures, we have discussed trying to simplify the figure captions but have not come up with a good solution. There is a lot of information contained within the figures and feel that further generalizations would not be clarifying. We do, however, acknowledge that the figure caption format is awkwardly long. What we propose is that we retain Fig 5 in the main body of the text as an example, and then move subsequent sister figures to Supplemental Materials. Please let us know if this satisfies your and the reviewer's comments to reduce the length of the manuscript.

Comments to the Author

5. Review Comments to the Author

Reviewer #1: In this study a conceptual model is used to aid the development of best practices of large river monitoring programs. The model was developed based on former scientific works and during scientist's workshop negotiations. Case study applications prove that the application of this complex conceptual model can be useful to identify critical information gaps, which can then be used to develop management and monitoring objectives.
I like the approach of developing such conceptual models, which can reveal information gaps, and think that the model in general can be useful to adopt across large river systems with some refinements and local adaptations. Consequently, I believe showing such an approach can provide useful information for the readers.
1)What I lack is to show more convincingly how such complex models can be used for multispecies systems, where not only the requirements of a single species is evaluated, which in fact the more realistic situation. How can individual species level models be put together to provide meaningful information for management? It would be useful to discuss this in more detail in the Discussion section.

Response: Thank you for this comment. We have added text in the discussion that describes how the CM could contribute to our understanding of the need for a multi-

species context in management activities. Please see: L645-691

2)Also a critical issue which should be briefly discussed is how the identified critical target variables should be prioritized, especially in a multispecies systems, where several variables will appear. Development of this section could convince the reader and could clearly show the applicability of such conceptual models by management.

Response: Thank you for this insight. We have provided language in the discussion that describes how the CM could be used to identify critical target variables in a multispecies context. Please see: L650-668

3)Although channel morphology/hydraulics may contain fragmentation/connectivity issues this should be made more clear in the material, because this is one of the most critical issue, which determine fish (meta)population or metacommunity dynamics. In fact fragmentation is often used as one of the most critical variable of anthropogenic drivers and as such is a critically important target to mitigate by management. However, it does not appear either in Fig 2 or Fig 3, but only on the case study figures belonging to morphology/hydraulics TIER1 components.

Response: Thank you. We agree with your assessment. We have further emphasized the importance of fragmentation by specifically mentioning it in the manuscript section describing the CM form. Please see: L223-234. Also, in Table 2, habitat fragmentation is emphasized as affecting multiple facets of the CM in multiple river systems and is mentioned in the text describing Table 2. This result indicates the need to better study the effects of habitat fragmentation on multiple biotic components.

Reviewer #2: Dear Editor,
This study demonstrates how a conceptual model can be used to identifying knowledge gaps in the mechanisms by which Essential Ecosystem Characteristics influence large-river fish species in the USA. These gaps should then be filled to improve the effectiveness of restoration and management.
I agree on the value of these conceptual models to identify knowledge gaps and inform decisions on what to monitor to fill them and allow a better understanding of the system and, therefore, enhance our capacity to manage them adequately. However, I disagree with some of the arguments:
1)The conceptual model represents potential interactions across different structural element of the river system but does not allow quantitative evaluations of strength of those interaction. As such, the value of is conceptual model is limited to identifying knowledge gaps and cannot be used to evaluate the relative importance of each interaction. Therefore, this conceptual model should only be used for identifying knowledge gaps and not for decision-making, as argued (see L771-773), beyond monitoring.

Response: This is an excellent point and we have removed the statement in L771-773 and elaborated on the considerations that need to be accounted for, and the difficulties with, assessing benefit:cost ratios. Please see: L692-726. Also, you are correct that the CM does not provide quantitative evaluations of the strength of the relations. We do acknowledge this and suggest that the CM could provide a basis for developing quantitative assessments in L749-754 and have added language that describes how the CMs could be the basis for Structural Decision Making and Adaptive Management processes (see L727-741).

2)The conceptual model lacks a cost analysis to evaluate the most efficient way of filling knowledge gaps. Some of the gaps might be more difficult/ costly or even feasible to fill. Without such analysis we can only identify the gaps but cannot prioritise where to focus monitoring on a cost-effective way and just confirm where gaps exist.

Response: Thank you for this comment. In addition to addressing the benefit:cost issue above, we have provided language in the discussion that describes how the CM could be used to identify critical target variables in a multispecies context but that there are critical uncertainties that need to be considered. See: L650-668. We agree with you about prioritizing based on cost effectiveness but respectfully suggest that the CM could provide information that would suggest where to focus monitoring effort.

Powered by Editorial Manager® and ProduXion Manager® from Aries Systems Corporation

	3)Three of the case studies present conceptual models for individual species. While I see the value of developing these conceptual models for charismatic endangered species, I wonder how feasible/ useful it would be this method when facing management needs for many species simultaneously. One of the case studies does present a conceptual model for the full fish community, but focused on diversity, rather than individual species, so no information of particular species issues are addressed. Would it be feasible to elaborate a conceptual model that addressed all individual species needs/ issues simultaneously? This would allow identifying knowledge gaps common to multiple species simultaneously.<br><br>Response: Thank you for this comment. We have added text in the discussion that describes how the CM could contribute to our understanding of the need for a multi-species context in management activities. Please see: Please see: L645-691<br><br>4)Minor comments:<br>- L304 & 369. What does ATV stand for?<br><br>Response: Thank you for bringing this to our attention. ATV stands for all-terrain vehicle. We have removed the acronym from the revision.<br><br>- The manuscript is quite long, especially because of the description of each case study. It would be good to present the information of these case studies in a more synthetic way (maybe on a table?).<br><br>Response: Thank you for the comment. Per your and the Associate Editor's recommendation we have pulled out some of the contextual information from the case studies and moved the information to an Appendix in Supplemental Information. We have also moved three figures and associated captions to the supplemental information section.<br>____
Additional Information:	
Question	Response
**Financial Disclosure**<br><br>Enter a financial disclosure statement that describes the sources of funding for the work included in this submission. Review the submission guidelines for detailed requirements. View published research articles from *PLOS ONE* for specific examples.<br><br>This statement is required for submission and **will appear in the published article** if the submission is accepted. Please make sure it is accurate.	This work was funded in part by U.S. Geological Survey's Core Science Systems Mission Area. This research also was conducted using in-kind contributions of the Ball State University, Illinois Natural History Survey, Oregon Department of Fish and Wildlife, Pacific Northwest Aquatic Monitoring Partnership, the Oklahoma and Alabama Cooperative Fish and Wildlife Research Units, and the U.S. Geological Survey Columbia Environmental Research Center, Grand Canyon Monitoring and Research Center, Oregon Water Science Center, Upper Midwest Environmental Sciences Center, and Western Fisheries Research Center.

Powered by Editorial Manager® and ProduXion Manager® from Aries Systems Corporation

**Unfunded studies**<br>Enter: *The author(s) received no specific funding for this work.*<br><br>**Funded studies**<br>Enter a statement with the following details:<br>• Initials of the authors who received each award<br>• Grant numbers awarded to each author<br>• The full name of each funder<br>• URL of each funder website<br>• Did the sponsors or funders play any role in the study design, data collection and analysis, decision to publish, or preparation of the manuscript?<br>• **NO** - Include this sentence at the end of your statement: *The funders had no role in study design, data collection and analysis, decision to publish, or preparation of the manuscript.*<br>• **YES** - Specify the role(s) played.<br><br>* typeset	
**Competing Interests**<br><br>Use the instructions below to enter a competing interest statement for this submission. On behalf of all authors, disclose any competing interests that could be perceived to bias this work—acknowledging all financial support and any other relevant financial or non-financial competing interests.<br><br>This statement is required for submission and **will appear in the published article** if the submission is accepted. Please make sure it is accurate and that any funding sources listed in your Funding Information later in the submission form are also declared in your Financial Disclosure statement.<br><br>View published research articles from *PLOS ONE* for specific examples.	The authors have declared that no competing interests exist.

**NO authors have competing interests**<br><br>Enter: *The authors have declared that no competing interests exist.*<br><br>**Authors with competing interests**<br><br>Enter competing interest details beginning with this statement:<br><br>*I have read the journal's policy and the authors of this manuscript have the following competing interests: [insert competing interests here]*<br><br>* typeset	
**Ethics Statement**<br><br>Enter an ethics statement for this submission. This statement is required if the study involved:<br><br>• Human participants<br>• Human specimens or tissue<br>• Vertebrate animals or cephalopods<br>• Vertebrate embryos or tissues<br>• Field research<br><br>Write "N/A" if the submission does not require an ethics statement.<br><br>General guidance is provided below. Consult the submission guidelines for detailed instructions. **Make sure that all information entered here is included in the Methods section of the manuscript.**	This research involved the conduct of a workshop where the participants came together as colleagues and subject matter experts to explore the research topic. There was no experimentation done on any of the humans involved in this collaborative effort.

Powered by Editorial Manager® and ProduXion Manager® from Aries Systems Corporation

**Format for specific study types**<br><br>**Human Subject Research (involving human participants and/or tissue)**<br>• Give the name of the institutional review board or ethics committee that approved the study<br>• Include the approval number and/or a statement indicating approval of this research<br>• Indicate the form of consent obtained (written/oral) or the reason that consent was not obtained (e.g. the data were analyzed anonymously)<br><br>**Animal Research (involving vertebrate animals, embryos or tissues)**<br>• Provide the name of the Institutional Animal Care and Use Committee (IACUC) or other relevant ethics board that reviewed the study protocol, and indicate whether they approved this research or granted a formal waiver of ethical approval<br>• Include an approval number if one was obtained<br>• If the study involved *non-human primates*, add *additional details* about animal welfare and steps taken to ameliorate suffering<br>• If anesthesia, euthanasia, or any kind of animal sacrifice is part of the study, include briefly which substances and/or methods were applied<br><br>**Field Research**<br><br>Include the following details if this study involves the collection of plant, animal, or other materials from a natural setting:<br>• Field permit number<br>• Name of the institution or relevant body that granted permission	
**Data Availability**<br><br>Authors are required to make all data underlying the findings described fully available, without restriction, and from the time of publication. PLOS allows rare exceptions to address legal and ethical concerns. See the PLOS Data Policy and FAQ for detailed information.	Yes - all data are fully available without restriction

A Data Availability Statement describing where the data can be found is required at submission. Your answers to this question constitute the Data Availability Statement and **will be published in the article**, if accepted.<br><br>**Important:** Stating 'data available on request from the author' is not sufficient. If your data are only available upon request, select 'No' for the first question and explain your exceptional situation in the text box.<br><br>Do the authors confirm that all data underlying the findings described in their manuscript are fully available without restriction?	
**Describe where the data may be found in full sentences. If you are copying our sample text, replace any instances of XXX with the appropriate details.**<br><br>• If the data are **held or will be held in a public repository**, include URLs, accession numbers or DOIs. If this information will only be available after acceptance, indicate this by ticking the box below. For example: *All XXX files are available from the XXX database (accession number(s) XXX, XXX.*).<br>• If the data are all contained **within the manuscript and/or Supporting Information files**, enter the following: *All relevant data are within the manuscript and its Supporting Information files.*<br>• If neither of these applies but you are able to provide **details of access elsewhere**, with or without limitations, please do so. For example:<br><br>*Data cannot be shared publicly because of [XXX]. Data are available from the XXX Institutional Data Access / Ethics Committee (contact via XXX) for researchers who meet the criteria for access to confidential data.*<br><br>*The data underlying the results presented in the study are available from (include the name of the third party*	All relevant data are within the manuscript and its Supporting Information files.

and contact information or URL).<br>• This text is appropriate if the data are owned by a third party and authors do not have permission to share the data.<br><br>* typeset	
Additional data availability information:	

Powered by Editorial Manager® and ProduXion Manager® from Aries Systems Corporation

Manuscript
Click here to access/download;Manuscript;Counihan_et al. LR monitoring information needs.docx

Title: Identifying monitoring information needs that support the management of fish in large rivers

Timothy D. Counihan, U.S. Geological Survey, Western Fisheries Research Center, Columbia River Research Laboratory, 5501A Cook-Underwood Road, Cook, WA 98605, email: tcounihan@usgs.gov, Phone:509-538-2299, Fax:509-538-2843, ORCID: 0000-0003-4967-6514

Kristen L. Bouska, U.S. Geological Survey, Upper Midwest Environmental Sciences Center, La Crosse, WI 54603, ORCID: 0000-0002-4115-2313

Shannon K. Brewer, U.S. Geological Survey, Alabama Cooperative Fish and Wildlife Research Unit, Auburn, AL 36849, ORCID: 0000-0002-1537-3921

Robert Jacobson, U.S. Geological Survey, Columbia Environmental Research Center, Columbia, MO 65201, ORCID: 0000-0002-8368-2064

Andrew F. Casper, Illinois Natural History Survey, Illinois River Biological Station, Havana, IL, 62644, ORCID: 0000-0003-3450-0209

Colin G. Chapman, Oregon Department of Fish and Wildlife, Ocean Salmon and Columbia River Program, Clackamas, OR 97015, ORCID: 0000-0002-6173-250X

Ian R. Waite, U.S. Geological Survey, Oregon Water Science Center, Portland, OR 97201, ORCID: 0000-0003-1681-6955

Kenneth R. Sheehan, U.S. Geological Survey, Grand Canyon Monitoring and Research Center, Flagstaff, AZ 86001, ORCID: 0000-0003-0362-0793

Mark Pyron, Ball State University, Muncie, IN 47306, ORCID: 0000-0003-0451-7827

Elise R. Irwin, U.S. Geological Survey, Alabama Cooperative Fish and Wildlife Research Unit, Auburn, AL 36849, ORCID: 0000-0002-6866-4976

Karen Riva-Murray, U.S. Geological Survey, Northeast Region, Troy, NY 12180, ORCID: 0000-0001-6683-2238

Alexa J. McKerrow, U.S. Geological Survey, Science Analytics and Synthesis, Core Science Systems, Raleigh, NC 27695, ORCID: 0000-0002-8312-2905

Jennifer M. Bayer, U.S. Geological Survey, Northwest-Pacific Island Region, Cook, WA 98605, ORCID: 0000-0001-9564-3110

Abstract – Management actions intended to benefit fish in large rivers can directly or indirectly affect multiple ecosystem components. Without consideration of the effects of management on non-target ecosystem components, unintended consequences may limit management efficacy. Monitoring can help clarify the effects of management actions, including on non-target ecosystem components, but only if data are collected to characterize key ecosystem processes that could affect the outcome. Scientists from across the U.S. convened to develop a conceptual model that would help identify monitoring information needed to better understand how natural and anthropogenic factors affect large river fishes. We applied the conceptual model to case studies in four large U.S. rivers. The application of the conceptual model indicates the model is flexible and relevant to large rivers in different geographic settings and with different management challenges. By visualizing how natural and anthropogenic drivers directly or indirectly affect cascading ecosystem tiers, our model identified critical information gaps and uncertainties that, if resolved, could inform how to best meet management objectives. Despite large differences in the physical and ecological contexts of the river systems, the case studies also demonstrated substantial commonalities in the data needed to better understand how stressors affect fish in these systems. For example, in most systems information on river discharge and water temperature were needed and available. Conversely, information regarding trophic relationships and the habitat requirements of larval fishes were generally lacking. This result suggests that there may be a common need for a better understanding of certain factors across large-river systems.

Introduction

Long-term monitoring has benefited a variety of marine and freshwater ecosystems, including large rivers like the Ohio [1, 2] and Illinois rivers [3]. Large-river systems are complex, making the development of effective monitoring programs especially difficult. Large rivers are dynamic systems with high variability in spatio-temporal physicochemical characteristics and biotic assemblages [4]. The inherent complexity of large rivers makes biotic assemblages logistically difficult to sample [5] and the mechanisms of change difficult to understand. Large rivers represent the culmination of vast stream networks and, thus, integrate and accumulate the effects of multiple stressors at varying spatial scales [6]. The spatial and temporal complexity associated with large rivers has hindered the identification of mechanisms driving declining populations of aquatic species [7-10]. To exacerbate the complexity, large rivers commonly have within-channel structural alterations (e.g., dams, river training structures, [11]) and often exhibit legacy effects from historical land uses [12]. To deal with the complexity, some areas of aquatic science recommend monitoring be used to test the linkages developed first through conceptual models (e.g., environmental flows, [13-15]).

Conceptual models are useful tools to help guide the design of monitoring programs [16]. The identification of questions relevant to conservation and management efforts requires some foresight and knowledge of the complexities of the system being monitored. For example, it is generally well accepted that the native range of the federally-listed Arkansas River Shiner (*Notropis girardi)* is truncated [17], though there is uncertainty surrounding the multiple threats affecting the species [18]. Reducing the uncertainty associated with the decline of the Arkansas River Shiner through the implementation of a hypothesis-driven monitoring program would

facilitate confidence in moving forward with a recovery plan. This is where conceptual models are quite useful; they can serve as the foundation to guide hypothesis-driven monitoring programs [14, 16] and identify key ecosystem processes and factors that may directly or indirectly affect management outcomes [19-22].

Understanding factors affecting the status and trends of fishes is of interest to ~~multiple~~ stakeholder groups across multiple jurisdictions. Fishes provide economic benefits to businesses that serve recreational interests, commercial and recreational fishers, tribal members for whom fish are an integral part of their cultural identity [23], and to local and state governments who derive revenue from these activities. Fish populations are affected by the integration of physical habitat, water quality, environmental contamination, habitat fragmentation, and overall ecosystem productivity [24-27]. Consequently, fish are often the focus of management and monitoring programs (e.g., [28]). However, because fish integrate the effects of so many components of the ecosystem, the success of efforts to manage fishes can be affected by unintended consequences of mitigation on factors not directly targeted by the actions. Without consideration of the effects of management on non-target ecosystem components, unintended consequences may limit management efficacy.

Our goal is to demonstrate how a structured, yet flexible, conceptual model (CM) can be used to identify the types of monitoring information needed to understand the range of factors affecting large-river fishes. Our CM includes a hierarchically structured conceptualization of ecosystem characteristics based on CMs originally developed by Harwell, Myers (29) and elaborated by Jacobson and Berkley (30). We chose to incorporate the tiered conceptualization of ecosystem characteristics proposed by Jacobson and Berkley (30) in part because it allows users to define their own biotic or abiotic interests. In this paper, we discuss the structure and

development of the CM. We apply the CM to case studies to illustrate the flexibility and applicability of this approach and use it to identify monitoring information needs specific to disparate management goals. More specifically, for each case study, we use the CM to hypothesize how human activities affect fish populations and then identify information needs required to evaluate the hypothesized relationships. We then posit the spatial and temporal scales of the management goal addressed in the conceptual model, inferences needed to inform the management goal, and data collection requirements needed to make the inferences.

Conceptual Model

Overview of Approach

Since 2012, scientists working on large rivers across the United States have participated in a forum intended to improve our understanding of large-river ecosystems. The collaborative forum has worked to identify best practices of long-term monitoring programs [31] and evaluate trends in fish assemblages across rivers [32]. As this group of scientists moved toward linking changes in fish populations and assemblages to human activities, there was a need to develop a process to help identify and prioritize the information needed to assess trends in large river fishes. To that end, a workshop was convened in Hood River, Oregon in May 2017, to jointly adapt, apply, and qualitatively evaluate a conceptual model for developing hypotheses that detail stressors affecting fishes arising from natural and anthropogenic sources [33].

Our general approach was to first identify human activities that affect large-river fishes and then hypothesize how the activities related to physical and chemical factors and biological communities. Prior to the workshop, we elicited opinion from experts that comprise a U.S.

Geological Survey (USGS) led forum on large river monitoring about important anthropogenic activities that could affect fish populations or communities in the river basins they work in. (Fig 1). We summarized the information from this exercise and grouped the anthropogenic activities into driver categories (Fig 2) and proposed a general form of the CM. We then disseminated the information to the experts prior to the workshop.

Fig 1. Map of rivers and watersheds represented by scientists that convened to develop a conceptual model that depicts how natural and anthropogenic drivers interact with habitats, biological systems, and fish in large rivers. River segments where we conducted case studies that applied the conceptual model to identify monitoring information needs associated with management goals are highlighted in red.

Fig 2. The results of a query to scientists from the Canadian, Colorado, Columbia, Hudson, Illinois, Ohio, Missouri, Red, Tallapoosa, Upper Mississippi, and Wabash Rivers, U.S to identify anthropogenic activities that affect large-river fishes in the river systems they represent. Anthropogenic activities were classified into five driver categories.

During the workshop we discussed and refined the CM form (Fig 3). We then had representatives from each river system represented at the workshop choose a management goal to address. Then, through a facilitated discussion led primarily by the representative of the river system being addressed, we 1) elaborated tiered conceptualizations of ecosystem characteristics to reflect the large-river systems and management goals being examined, 2) used knowledge of the fish species' life history and population bottlenecks to relate biological ecosystem characteristics to habitat requirements, 3) hypothesized pathways describing how anthropogenic

and natural drivers affect large-river fish populations either indirectly (e.g., effects on flow regime, habitat, trophic resources, etc.) or directly (e.g., competition with invasive species), and 4) hypothesized interactions within ecosystem characteristic tiers that could affect the management goal. Based on this exercise, we chose four case studies to refine for use in this manuscript (Fig 1).

Fig 3. Tiered hierarchical conceptualization of how anthropogenic and natural drivers relate to physical and biological components of large-river ecosystems. Essential ecosystem characteristics (EECs) are groupings of ecosystem components. Tier 1 EECs represent physical and chemical effects; fundamental measures of process that are directly affected by anthropogenic and natural drivers. Tier 2 EECs represent a broad habitat category that is intended to encompass the physical, chemical, and biological components of the riverine habitats that influence reproduction, growth, and survival of biotic communities. The Tier 3 EEC represent components of the biological systems that respond to changes in the hierarchical components of the conceptual model.

After the workshop, we held a series of conference calls with workshop participants with expertise in the selected case studies to refine all aspects of the CMs and associated information. During the calls, we started with the CM from the workshop and discussed and clarified CM components, pathways, and inter-tier interactions. We then characterized whether, based on the expert knowledge of workshop participants, there was a strong, moderate, or weak understanding of the pathways and interactions. A list of the information needed to understand the relationships described in the CM was developed. We then had the case study leader classify whether the data

required to understand the information needs were available, insufficient, or not available. For information needs that were classified as insufficient or not available, we characterized the spatial and temporal scales at which data should be collected to make inferences that support the evaluation of management goals.

We then encouraged representatives from each basin to share the CM and the case study narrative with other experts familiar with the river system and management goal. This outreach took several forms including sharing the CM with working groups tasked with implementing the management goal, discussions with peers familiar with the management goal, and presenting the CM at regional conferences. The intent was to garner opinions from outside the workshop participants. If needed, the CMs incorporated the feedback received.

Hierarchical structure of Conceptual Model

Our CM is a hierarchical conceptualization of how anthropogenic and natural drivers relate to multiple tiers representing the physical and biological components of large-river ecosystems (Fig 3). Natural drivers included in the CM were physiographic, climatic, and biogeographic factors that control fluxes of water, mass, energy, and genetic information in a watershed [30]. The physiographic factors, such as lithology, soils, and watershed topography, exert control on water, sediment, and geochemical fluxes (e.g., nutrients) into the river corridor. Physiography is generally static over time frames of decades to centuries. Climate controls fluxes of atmospheric energy and moisture into the watershed. Unlike physiography, climate is more likely to vary over relatively shorter temporal scales. Biogeography describes the native organism assemblage in the watershed (e.g., [34]) and the natural flux of genetic information due

to immigrations, emigrations, mutations, and extinctions. Changes arising from the biogeography driver includes altered spatial distribution of organisms within the watershed, which, in turn, may alter the effects of natural system regimes on the river corridor. For example, natural variation of the type and distribution of vegetation can affect the time series and magnitude of runoff events [30].

We created five categories of anthropogenic drivers to characterize a range of human activities that affect large rivers: land use, commercial use, biological community alteration, water use, and recreation (Fig 2). The land-use category is intended to reflect different ways humans use landscape resources that affect large rivers. We defined commercial use as the use of river resources for marketable enterprises that did not involve water removal or transfer. We included biological community alteration to represent the intentional or non-intentional human alteration or manipulation of the river's biological community (e.g., introductions of non-native fish). Recreational use was defined as the use of river resources for leisure activities (e.g., fishing, boating). We considered water use a direct commercial or non-commercial use of river water that involved the removal or transfer of water.

Our CM includes hierarchically structured essential ecosystem characteristics (EEC), originally developed by Harwell, Myers (29) and described in detail by Jacobson and Berkley (30). Briefly, EECs are characteristics that can be classified into similar groups based on the way they link to biological endpoints [30]. Tier 1 EECs are measurable characteristics that describe processes that can significantly alter the morphological or chemical characteristics within a river channel. The Tier 1 categories we considered were 1) hydrology, 2) channel morphology/hydraulics, 3) sediment transport, and 4) biogeochemistry/thermodynamics. Tier 2 EECs are broadly described as physicochemical or biological components of "habitat" that are

hypothesized to affect (e.g., growth, survival, reproduction, [35]) fish populations or assemblages. Lastly, Tier 3 EECs represent components of the hypothesized biological system that are affected by the cascading (e.g., degradation of egg quality caused by increases in sediment deposition) or direct (e.g., predation by invasive species) effects of anthropogenic and natural drivers. Tier 2 characteristics are particularly important because these are the factors that can be examined at scales most often sampled by fisheries managers [36]. The specific components that comprise Tier 2 and 3 EECs are flexible and can be adapted and elaborated depending on the river system and specific management goal being addressed.

We retained aspects of the approach taken by Jacobson and Berkley (30) with respect to how our model represents interactions between drivers and EECs, but with key differences. Since we were interested in representing how human activities affect large river ecosystems, our approach acknowledges that anthropogenic and natural drivers interact and alter the expected characteristics of Tier 1 EECs. Similar to Jacobson and Berkley (30), our model depicts a stress associated with a natural or anthropogenic driver to Tier 1 EECs as fluxes in natural system regimes that alter the frequency, magnitude, duration, timing, or rate of change in natural systems or by the imposition of a hard-structural constraint on channel form. The natural system regimes considered in our CM were hydraulic, hydrologic, sediment, temperature, light, and biogeochemistry. Graphically, the natural system fluxes were represented by arrows connecting anthropogenic and natural drivers to Tier 1 EECs. Similarly, hypothesized pathways between EECs, that depict the expression of the cascading effects of anthropogenic and natural drivers, and interaction within EECs were depicted as arrows. For example, fragmentation of river systems resulting from altered hydrologic and/or hydraulic regimes caused by dams, weirs, levees, and other factors are frequently cited sources of stress to large-river fishes [37].

Fragmentation can prevent fish from migrating and/or dispersing through their natural reproductive ranges and from accessing critical habitats [38]. To depict a scenario where the presence of a dam is altering hydrologic and/or hydraulic regimes resulting in habitat fragmentation, the CM would show an arrow from an anthropogenic stress (i.e., dam as a commercial activity) to a Tier 1 EEC (e.g., channel morphology/hydraulics) that would depict a natural system flux (e.g., altered hydrologic and/or hydraulic regime) that would then manifest as a stress caused by habitat fragmentation depicted by an arrow between the Tier 1 EEC and a Tier 2 EEC (e.g. habitat) that would then manifest as an effect on a Tier 3 component, shown by an arrow between Tier 2 and Tier 3. All stress pathways and interactions were classified with respect to the strength of understanding of the relationships based on expert opinion. Arrows with solid blue lines depict a strong understanding of the relationship, dotted-dashed blue lines represent a moderate understanding of the relationship, and with a black dashed line represent a weak understanding of the relationship.

Spatial and temporal context

The successful characterization of how human activities influence large-river fishes is dependent upon integrated concepts of scale. Fish distributions in rivers can vary spatially within river basins in relation to naturally occurring and human induced landscape characteristics [32]. Fish distributions can also vary seasonally, annually, and over longer times in response to changing environmental conditions [39]. Consequently, the spatial and temporal scope of fish management goals often varies within and between large-river systems and agencies. For data collected by monitoring programs to have the highest relevance, the spatial and temporal scales

appropriate for scientific investigation and management must also be time and geographic-context specific. For example, the management of White Sturgeon (*Acipenser transmontanus*) in the Columbia River varies by reservoir or river segment and season [40, 41]. The effects of hydropower development on White Sturgeon vary spatially and temporally as well, so the spatial and temporal context of the data needed to understand the effects needs to be considered. For instance, hydropower peaking operations, that can vary by dam and season, affect river discharge in a river reach on a diel and even hourly basis [42], whereas water storage and other management actions can affect seasonal discharges over a broader geographic scale [40]. Understanding the spatial and temporal context needed to inform management will help ensure relevant information is collected.

We considered spatial and temporal resolution in our CMs. We defined spatial extent as : local network – synonymous with Hydrologic Unit Code (HUC) 2 basins [43, 44]; segment – the portion of a river between two major tributary confluences [45] or other hydrogeomorphic features [46]; reach - the length of river occurring between breaks in channel slope caused by man-made dams or other hydrogeomorphic features [45]; patch – an area used by an organism (e.g., for reproduction or resource attainment) that can vary both spatially and temporally depending on the species of interest [47, 48]. For our purposes, the spatial scales considered are nested such that segments occur within local networks, reaches occur within segments, and patches occur at the sub-reach scale. Temporal units considered were daily, seasonal, annual, and decadal. The temporal units were used to denote both the scale of inferences needed to support the management goal and the scale at which data should be collected to inform the inferences.

Case Studies

We applied the CM to four case studies. For each, we followed the pathways of stress from Tier 1 EECs to the biological endpoint associated with the management goal to identify information needs. We then characterized the spatial and temporal scales of the management goal, the scientific inferences needed to inform the management goal, and that data collection needs to occur to support the inferences for monitoring information needs identified as requiring additional data for each of the case studies. To summarize similarities across case studies, we generalized the stressors and inter- tier interactions identified in the case studies and then summarize the similarities by EEC tier. More context for the river systems characterized in the case studies can be found in Appendix S1.

South Canadian River

Native populations of the federally-threatened Arkansas River Shiner are believed to be restricted to two fragmented portions of the South Canadian River [49]. The Arkansas River Shiner is hypothesized to be affected by several anthropogenic activities that primarily affect water quality and quantity (Fig 4). Three reservoirs on the South Canadian River have altered discharge patterns (Fig S1), and fragmented river habitats. Two known native populations of Arkansas River Shiner occupy the two remaining river segments of sufficient length and complexity to allow eggs to drift the time required to successfully complete their early life history. Small impoundments for agriculture use, road crossings, groundwater pumping and other local water extractions (e.g., oil and gas) threaten to further fragment existing habitat. Fragmentation could also be problematic for upstream fish migrations; there is some evidence that Arkansas River Shiners migrate upstream to spawn to achieve adequate drift distances for

their offspring [50]. It has also been speculated that this species might benefit from access to floodplain habitats [51], but we are unaware of efforts to examine that hypothesis. Changes in the flow patterns may also relate to the expansion of salt cedar *Tamarix* spp. and other non-native riparian species that constrain the channel and inhibit channel habitat complexity [52, 53]. Changes to the riparian corridor can also alter the availability of drifting invertebrates for Arkansas River Shiner feeding (i.e., Coleoptera, Hymenoptera; [54]). Channel complexity acts to slow the transport of eggs [49] and may prevent eggs from being washed into downstream reservoirs where survival is hypothesized to be extremely low. Climate change is expected to increase the intensity and frequency of drought events within this region [55, 56], which may exacerbate habitat fragmentation, promote all-terrain vehicle traffic within the river channel causing direct mortality on stranded fish (Gene Wilde, Texas Tech University, Personal Comm.), and concentrate contaminants and salinity [57]. The tolerances of Arkansas River Shiner to salinity concentrations and many other contaminants are unknown (see Table S1; [18]). Lastly, introductions of non-native fishes have occurred within the basin. The primary concern is the presence of Red River Shiner (*Notropis bairdi*) because it is suspected to reproduce in a similar manner and be a possible competitor to the Arkansas River Shiner [57].

Fig 4. Conceptual model describing the relationship of natural and anthropogenic drivers to essential ecosystem characteristics (EECs) affecting the recruitment of the Arkansas River Shiner in the South Canadian River in New Mexico, Texas, and Oklahoma. Essential ecosystem characteristics are groupings of ecosystem components. Tier 1 EECs represent physical and chemical effects; fundamental measures of process that are directly affected by anthropogenic and natural drivers. Tier 2 EECs represent a broad habitat category that is intended to encompass

the physical, chemical, and biological components of the riverine habitats that influence reproduction, growth, and survival of biotic communities. The Tier 3 EECs represent components of the biological systems that respond to changes in the hierarchical components of the conceptual model. The strength of our understanding of how natural and anthropogenic drivers interact with habitats, biological systems, and fish in large rivers is represented by the different types of lines in the figure. Solid blue lines depict a strong understanding of the relationship, the dotted-dashed blue line represents a moderate understanding of the relationship, and the black dashed line represents a weak understanding of the relationship. The different types of lines also represent the strength of our understanding of within EEC-tier relationships.

The results of the CM exercise that characterized factors affecting the Arkansas River Shiner in the South Canadian River suggested the critical life-history bottlenecks for the Arkansas River Shiner are successful spawning and recruitment to the first year. Impediments that limit our understanding of factors that lead to successful spawning and recruitment included the effects of channel morphology and hydraulics on the quality and quantity of larval rearing habitat, and subsequent effects on larval production (Table S1). Water use and other drivers occurring at relatively coarse spatial and temporal scales are the hypothesized drivers related to degradation of reproductive habitat for the Arkansas River Shiner (Fig 5). A temporal lag in responses at finer scales (i.e., improved habitat) would be anticipated with management actions at these coarser spatial scales (e.g., water releases from dams); though, providing connectivity via minimal water releases would occur relatively quickly. Although there are gages on the South Canadian River, the spacing of the gages is not sufficient to have a full understanding of flow patterns between the gages given the semi-arid nature of the basin and potential for reaches

to be affected by water withdrawals such as groundwater pumping. Our understanding of the species life history is well established; however, the effects of human pressures on the species and associated habitat has not been well studied (i.e., production, survival). As stressors propagate through Tier 1 to Tier 2 and Tier 3, the level of uncertainty increased such that it is not possible to define a preferred hypothesis for Arkansas River Shiner recruitment failure. The status of information needed to understand the hypothesized stress pathways and interactions was mostly characterized as insufficient or not available (Table S1).

Fig 5. The spatial and temporal scales of the management goal, the scientific inferences needed to inform the management goal, and that data collection needs to occur to support the inferences for monitoring information needs identified as requiring additional data in the case study addressing the recruitment of the Arkansas River Shiner in the Canadian River, Oklahoma (see Table S1 for additional detail). A:Tier 1 EEC=channel morphology/hydraulics; Stressor=altered hydraulic regime; B:Tier 1 EEC=biogeochemistry/thermodynamics; Stressor=altered water temperature regime; C:Tier 1 EEC=biogeochemistry/thermodynamics; Stressor=altered biogeochemical regime; D:Tier 2 EEC=Arkansas River Shiner spawning habitat; Stressors=contaminants, water temperature, habitat fragmentation; E:Tier 2 EEC=larval Arkansas River Shiner habitat, Stressors=water temperature, habitat fragmentation and Tier 2 EEC=invertebrate habitat, Stressors=altered riparian plant community, discharge, sediment deposition; F:Tier 3 EEC=primary production, Stressor = nutrient flux; G:Tier 3 EEC=invertebrate production; Stressor=invertebrate habitat quantity and quality and Tier 3 EEC=Arkansas River Shiner larvae production, Stressor=predation by invasive species; H:Tier 1 EEC= biogeochemistry/thermodynamics; Inter-tier interaction= Sediment adsorption of

contaminants and nutrients and Tier 3 EEC=Arkansas River Shiner larvae production, Stressors=Arkansas River Shiner larvae habitat quantity and quality and Tier 3 EEC=Arkansas River Shiner age-0 recruitment; Inter-tier interaction=Arkansas River Shiner larvae mortality and Tier 3 EEC=Arkansas River Shiner age-1+ recruitment, Inter-tier interaction= Arkansas River Shiner age-0 mortality and Tier 3 EEC=all, Inter-tier interaction=trophic level interactions; I:Arkansas River Shiner larvae production; Stressor= direct mortality from recreational use (i.e., all-terrain vehicle and in-river traffic); J:Tier 3 EEC=Arkansas River Shiner egg quality and production; Stressor=Arkansas River Shiner spawning habitat quantity and quality; K:Tier 3 EEC=Arkansas River Shiner larvae production; Inter-tier interaction=Arkansas River Shiner egg mortality.

Colorado River

The Humpback Chub (*Gila cypha)*, a fish native to the Colorado River, was listed as endangered by the U.S. Fish and Wildlife Service in 1967 and given full protection under the Endangered Species Act of 1973 (ESA). To mitigate the effects of anthropogenic changes to the river on Humpback Chub, an understanding of the mechanisms by which Glen Canyon Dam and non-native species affect Humpback Chub is needed. A critical life-history bottleneck for Humpback Chub is recruitment into the first year class (Fig 6). Temperature, light, and seasonal high river discharge from snowmelt are thought to cue spawning behavior [58]. Hydropower development has dampened the range of river discharges of the Lower Colorado River within Grand Canyon. Historically, river discharge varied between 15 and 3400 m^3/s, however discharge was greater in large flood events; current dam operations limit flows to a range of 140

to 1000 m^3/s (Fig S2). Resulting changes in turbidity and water temperature create risks to endangered Humpback Chub, and other endemic fish. For example, the quantity and quality of habitat is reduced through changes in turbidity, biogeochemistry, and the temperature regime. Hypolimnetic water releases from Lake Powell maintain cold temperatures in the Colorado River downstream from Glen Canyon Dam; currently, spawning is limited to a single tributary, the Little Colorado River. As embryos survive into the larval stage, nursery habitats to support growth and foraging are essential [59-61]. A secondary risk to juvenile survival post-larval stage is predation by non-native species including Rainbow Trout (*Oncorhynchus mykiss*) and Channel Catfish (*Ictalurus punctatus*) [62]. Temperatures for well over 100 km downstream of Lake Powell are excellent for non-native, cold water species, including a closely managed world-class Rainbow Trout fishery at Lees Ferry. Rainbow and Brown Trout (*Salma trutta*) are currently managed as an invasive species downstream of the confluence of the Colorado and Little Colorado River, approximately 97 km downstream of Glen Canyon Dam, to mitigate predation upon native fishes including the endangered Humpback Chub.

Fig 6. Conceptual model describing the relationship of anthropogenic drivers to essential ecosystem characteristics (EECs) affecting the recruitment of Humpback Chub in the Colorado River between Glen Canyon Dam and Lake Mead, Arizona. Essential ecosystem characteristics (EECs) are groupings of ecosystem components. Tier 1 EECs represent physical and chemical effects; fundamental measures of process that are directly affected by anthropogenic and natural drivers. Tier 2 EECs represent a broad habitat category that is intended to encompass the physical, chemical, and biological components of the riverine habitats that influence reproduction, growth, and survival of biotic communities. The Tier 3 EECs represent

components of the biological systems that respond to changes in the hierarchical components of the conceptual model. The strength of our understanding of how natural and anthropogenic drivers interact with habitats, biological systems, and fish in large rivers is represented by the different types of lines in the figure. Solid blue lines depict a strong understanding of the relationship, the dotted-dashed blue line represents a moderate understanding of the relationship, and the black dashed line represents a weak understanding of the relationship. The different types of lines also represent the strength of our understanding of within EEC-tier relationships.

The CM exercise documented a high understanding of the relationships of anthropogenic drivers to Tier 1 EECs, cascading to multiple, less-understood hypotheses about how these factors would combine to affect habitats at Tier 2 (Fig 6). High confidence in the linkages from Tier 2 invertebrate habitat to Tier 3 insect production is followed by a lesser understanding of how insect production is linked to larval production. The uncertainty of the relations between food resources stands in contrast to high certainty that was ascribed to the linkages from larval chub habitat to larval chub production, and from spawning habitat to larval chub production and then to recruitment. Since 1997, the Glen Canyon Dam Adaptive Management Program has supported extensive monitoring and research across the spatial and temporal landscape of the Colorado River. As a result, the information needed to characterize some of the stressors is readily available (Table S2). However, the status of some existing information was characterized as insufficient or not available. For the information needs characterized as being insufficient or not available, we identified the spatial and temporal scales at which data collection would facilitate the inferences needed to inform the management goal (Fig S3). Understanding how

these knowledge gaps affect Humpback Chub recruitment could identify strategies that will help achieve the management goal of improving Humpback Chub recruitment in the Colorado River.

Columbia River

White Sturgeon is the largest freshwater fish in North America [63]. Like other sturgeon species, anthropogenic stressors have negatively affected White Sturgeon productivity. Our knowledge of factors affecting White Sturgeon productivity remain poorly understood [40]. Therefore, we used the CM to identify knowledge gaps associated with the hypotheses that dam construction and operation, land-use practices, and invasive species, in some combination, affect the recruitment of age-0 White Sturgeon (Fig 7). Within the basin, development of hydroelectric and water-storage dams have changed the magnitude and seasonality of the natural river discharge (Fig S4) and thermal regimes [64], reduced the quantity and quality of spawning habitats [65, 66], and disrupted historical migration patterns [67]. Prior to hydropower development, White Sturgeon experienced a hydrograph that peaked during June-July due to snowmelt [64]. However, from 1949 to 1993 the average discharge in June decreased from 14,000 m^3/s to 6,000 m^3/s and the maximum water temperature has increased by 1.8°C [64]. White Sturgeon likely used the natural hydrograph and thermal regime as cues to seek out optimal spawning habitats and initiate spawning [65, 68].

Fig 7. Conceptual model describing the relationship of anthropogenic drivers to essential ecosystem characteristics (EECs) affecting the recruitment of White Sturgeon in the Columbia River, U.S. Essential ecosystem characteristics (EECs) are groupings of ecosystem components. Tier 1 EECs represent physical and chemical effects; fundamental measures of process that are

directly affected by anthropogenic and natural drivers. Tier 2 EECs represent a broad habitat category that is intended to encompass the physical, chemical, and biological components of the riverine habitats that influence reproduction, growth, and survival of biotic communities. The Tier 3 EECs represent components of the biological systems that respond to changes in the hierarchical components of the conceptual model. The strength of our understanding of how natural and anthropogenic drivers interact with habitats, biological systems, and fish in large rivers is represented by the different types of lines in the figure. Solid blue lines depict a strong understanding of the relationship, the dotted-dashed blue line represents a moderate understanding of the relationship, and the black dashed line represents a weak understanding of the relationship. The different types of lines also represent the strength of our understanding of within EEC-tier relationships.

Factors other than river discharge and water temperature may also be affecting age-0 White Sturgeon recruitment [40]. In areas of the Columbia and Snake Rivers with hydropower development, White Sturgeon populations are functionally isolated by dams. Consequently, White Sturgeon depend on conditions within restricted reaches to sustain production. In some reaches, suitable rearing habitat exists, and individual growth rates are high, but spawning habitat is limited and recruitment of fish is poor [65]. In other reaches, favorable spawning conditions exist but growth of young fish may be density limited [69]. How the availability of food resources for larval and juvenile White Sturgeon varies among reservoirs may affect age-0 White Sturgeon recruitment. Research has also suggested that contaminants may affect White Sturgeon reproductive biology [70]. The introduction of non-native fishes has clearly affected the native fish assemblage in the Columbia River [32, 71]. Channel Catfish, Smallmouth Bass *Micropterus*

dolomieu, and Walleye (*Sander vitreus*) that have been introduced into the Columbia River have all been shown to prey upon or compete with native fish species [71, 72] and may also affect White Sturgeon.

The CM (Fig 7) provides structure to the multiple competing hypotheses and indicates how anthropogenic drivers may be affecting Tier 1, 2, and 3 EEC's. Not surprisingly, the strength of our understanding of the effects of stressors on White Sturgeon was greater for relationships between life stages that are more easily sampled (e.g., adults) and that rely on data that are readily accessible as part of monitoring associated with hydropower development (e.g., discharge and water temperature) or metrics that are from combinations of these variables (e.g., estimates of White Sturgeon spawning habitat; Table S3). However, for relationships between harder to sample White Sturgeon life stages (e.g., larvae), biota that require expertise and equipment atypical of traditional fisheries assessments in large rivers (e.g., benthic macroinvertebrates), or stressors that are described by metrics that require specialized modeling expertise (e.g., sediment transport dynamics), the existing information was insufficient or not available. For example, we identified the need to better understand the effects of channel morphology and hydraulics on benthic macroinvertebrate habitat, invertebrate production, and subsequent larval White Sturgeon production. The most certain pathways connected changes in hydrology, hydraulics, and temperature regimes to reduced spawning habitat in Tier 2, then to decreased egg quality and production at Tier 3.

Our results suggest there are stressors that can affect the management goal of increasing age-0 White Sturgeon recruitment that are poorly understood and that could confound efforts to manage White Sturgeon in the Columbia River. Our characterization of the spatial and temporal scales that data should be collected at could help guide future efforts to fill data gaps to support

the inferences needed to address the goal of improving recruitment of age-0 White Sturgeon (Fig S5).

Upper Mississippi and Illinois Rivers

In the agricultural Midwest, basin-wide land uses affect the delivery of sediments, nutrients, and runoff to the Upper Mississippi and Illinois rivers [73-76]. Within the floodplain of these two large rivers, agriculture and residential land uses often rely upon the use of levees to isolate productive or developed lands during seasonal high-flow events. Within the channel, these rivers support commercial navigation with locks and dams and river-training structures, which have dramatically altered channel morphology and hydraulics throughout the system. Together, the cumulative effects of these modifications to the basin, floodplain, and river have implications for habitat diversity and native fish biodiversity [77]. Additionally, recent invasion and expansion of non-native species, namely Silver Carp (*Hypophthalmichthys molitrix*) and Bighead Carp (*H. nobilis)*, have direct and indirect effects on native fishes that likely compound or confound stress pathways on native fish biodiversity [78, 79]. Therefore, we used the CM to explore how these primary anthropogenic drivers have likely influenced fish habitats and associated life stages (Fig 8).

Fig 8. Conceptual model of how anthropogenic drivers in the upper Mississippi and Illinois Rivers influence native fish habitats and recruitment. Essential ecosystem characteristics (EECs) are groupings of ecosystem components. Tier 1 EECs represent physical and chemical effects; fundamental measures of process that are directly affected by anthropogenic and natural drivers. Tier 2 EECs represent a broad habitat category that is intended to encompass the physical,

chemical, and biological components of the riverine habitats that influence reproduction, growth, and survival of biotic communities. The Tier 3 EECs represent components of the biological systems that respond to changes in the hierarchical components of the conceptual model. The strength of our understanding of the relationships of how natural and anthropogenic drivers interact with habitats, biological systems, and fish in large rivers is represented by the different types of lines in the figure. Solid blue lines depict a strong understanding of the relationship, the dotted-dashed blue line represents a moderate understanding of the relationship, and the black dashed line represents a weak understanding of the relationship. The different types of lines also represent the strength of our understanding of within EEC-tier relationships.

Increased sediment loads in combination with altered hydraulics and morphology have resulted in high rates of sedimentation, homogeneity of depth, and loss of low-velocity, off-channel areas [80, 81]. A diversity of off-channel habitat conditions (i.e., increased residence time, low velocity, warm temperatures, availability of food resources) support growth and development of larval and juvenile fishes [82, 83] and often provide important food resources for adult fishes [84-88]. Further, deep, low-velocity off-channel habitats are recognized as important refugia for a wide range of fishes during high-flow events and seasonal periods of low temperatures [89-92]. Loss of floodplain connectivity has eliminated the seasonal exchange of nutrients, organisms and organic matter between river and floodplain environments that support biological diversity and productivity [93, 94]. Reduced availability of spawning, nursery, foraging, or overwintering habitat conditions can serve as bottlenecks to fish populations through limited larval production, reduced growth, and increased overwinter mortality. For example, high sedimentation rates have been filling backwaters in the Illinois River for decades, thus limiting

the availability of overwintering conditions for fishes that bioenergetically need a deep refuge with slow water velocities. Missing year-classes in this reach, represented by truncated size structure in the Largemouth Bass (*Micropterus salmoides*) population are hypothesized to be a result of periodic winter mortality (Fig S6).

The application of our CM makes clear that while the general effects of anthropogenic drivers on hydrology, sediment transport, biogeochemistry ~~and~~ hydraulics and morphology are well understood, there is much less known about how those effects influence the quality and availability of required habitat conditions (Tier 2, Fig 8). Although there is likely overlap of habitat requirements among species with similar life histories, the diversity of habitat conditions necessary to support a native and diverse fish community has not been explored. Consequently, the existing information needed to assess the relationship between habitat quality and quantity, and egg production, juvenile recruitment, and adult survival of fish populations within the Upper Mississippi and Illinois rivers was categorized as insufficient to not available (Tier 3 Inter-tier interaction, Fig 8; Table S4). Addressing these knowledge gaps could improve the effectiveness of habitat restoration efforts focused on maintaining a diverse native fish community. The spatial and temporal scales of data collection that would support needed inferences to address restoring and maintaining native fish biodiversity and habitat quantity and quality are characterized in Fig S7.

Similarities across case studies

We observed similarities in the stressors and interactions within EEC tiers across the four case studies. For Tier 1, an altered hydrologic regime was identified as a stressor to the

hydrology EEC in all four rivers (Table 1). Presumably this is due to the ubiquitous effects of dams on the systems examined. However, in some rivers the altered hydrologic regime originated from other anthropogenic (e.g., water use, land use, biological community alteration) and natural (e.g., climate) drivers. Similarly, all four case studies listed an altered water temperature regime as a stressor to the biogeochemistry/thermodynamics EEC with linkages to several anthropogenic drivers (Fig 4, 6-8; Tables S1-S4). There were also similarities across case studies with respect to the identification of interactions between Tier 1 EECs with all four case studies noting interactions between Tier 1 EEC components.

Table 1. Stressors or inter-tier interactions affecting Tier 1 Essential Ecosystem Characteristics (EEC) identified as an information need in the application of the conceptual model to case studies in the Canadian River (1), Colorado River (2), Columbia River (3), and Upper Mississippi and Illinois rivers (4). Tier 1 EECs are measurable characteristics that describe processes that can significantly alter the morphological or chemical characteristics within a river channel.

	Tier 1 EEC			
Stressor or inter-tier interaction	Hydrology	Channel Morphology/Hydraulics	Sediment Transport	Biogeochemistry/ Thermodynamics
Altered Hydrologic Regime	1, 2, 3, 4	1	1, 2	
Altered Hydraulic Regime		1, 3, 4	3	
Altered Sediment Regime			3, 4	
Altered Water Temperature Regime				1, 2, 3, 4
Altered Biogeochemical Regime				1, 3, 4
Channel forming processes		1, 2, 3, 4		
Sediment transport dynamics		1, 2, 3, 4	1, 2, 3, 4	
Sediment adsorption of contaminants and nutrients				1, 2, 3, 4

For Tier 2, there were similarities across case studies; however, the adaptation and elaboration of the components to the management goal in the case studies was apparent (Table 2). The management goal associated with the case study for the Upper Mississippi and Illinois Rivers resulted in Tier 2 EEC components (e.g., overwintering habitat) and stressors (e.g., dissolved oxygen) that were unique. Spawning habitat was identified as a Tier 2 EEC component in all the case studies and multiple stressors were identified as affecting this component in two or more of the case studies. Larval fish and invertebrate habitat were noted as Tier 2 EEC components with some similarities in stressors across case studies. Habitat fragmentation, sediment deposition, and water temperature were listed as stressors to Tier 2 EECs in all four case studies. No interactions between Tier 2 EEC components were listed for the case studies.

Table 2. Stressors affecting Tier 2 Essential Ecosystem Characteristics (EEC) identified in the application of the conceptual model to case studies in the Canadian River (1), Colorado River (2), Columbia River (3), and Upper Mississippi and Illinois rivers (4). Tier 2 EECs are broadly described as physical, chemical, or biological components of "habitat" that are hypothesized to have overall fitness consequences.

	Tier 2 EEC (habitat)				
Stressor	Spawning	Overwintering	Juvenile fish	Larval fish	Invertebrate
Altered riparian community					1
Channel stability					2, 3
Contaminants	1, 3, 4		4		
Discharge					1
Dissolved oxygen	4	4	4		
Habitat fragmentation	1, 3, 4			1, 2, 3	2, 3
Sediment deposition	3, 4	4	4	3	1, 2, 3
Turbidity				2	
Water Depth	4		4		
Water temperature	1, 2, 3, 4	4	4	1, 3	
Water velocity	3, 4	4	4		

The adaptation of Tier 3 EECs and elaboration of the biological system related to the management goal addressed by the case studies resulted in EECs that were comprised of fish life stages ranging from eggs to adult fish, primary and invertebrate production, and biodiversity (Table 3). All Tier 3 EEC components, except biodiversity, were present in the four case studies. Not surprisingly, habitat quantity and quality were listed as stressors to all the EECs related to fish and invertebrates. Six of eight stressors or inter-tier interactions were listed as affecting fish larvae and five of eight were noted as affecting egg quantity and quality. In contrast to Tier 2, interactions were extensively noted between Tier 3 EECs and trophic level interspecific interactions were listed in all four case studies.

Table 3. Stressors or inter-tier interactions affecting Tier 3 Essential Ecosystem Characteristics (EEC) identified in the application of the conceptual model to case studies in the Canadian River (1), Colorado River (2), Columbia River (3), and Upper Mississippi and Illinois rivers (4). Tier 3 EECs represent components of the hypothesized biological system upon which the cascading effects of anthropogenic and natural drivers act, and interactions occur.

	Tier 3 EEC						
Stressor or inter-tier interaction	Adult fish recruitment	Juvenile fish recruitment	Larval fish production	Fish egg quality/ production	Invertebrate production	Primary production	Biodiversity
Direct mortality			1				
Predation/competition by invasive species			1, 2, 3	3			4
Habitat quantity/quality	4	4	1, 2, 3	1, 2, 3, 4	1, 2, 3		
Nutrient flux						1, 2, 3	
Trophic level interspecific interactions	1, 2, 3, 4	1, 2, 3, 4	1, 2, 3, 4	1, 2, 3, 4	1, 2, 3, 4	1, 2, 3, 4	4
Predation			3	3			
Mortality	1, 3, 4	1, 2, 3, 4	1, 2, 3				
Fish condition				1, 3, 4			

Discussion

The main objective of our exploration of CMs was to impose some structure on the complex ecosystems found in large rivers and from that structure, identify gaps in monitoring information that could inform the management of fish. Comparison across our four case studies provides some insights into large rivers and the utility of the CM to identify gaps in our understanding of factors affecting fish in large rivers.

Despite large differences in the physical and ecological contexts of the river systems, the case studies demonstrated substantial commonalities in the data needed to better understand how human activities affect these systems and ~~in the application of~~ the CM. The general tiered structure of drivers and cascading responses through EECs worked well with the four examples. Each of the four rivers could be placed in the tiered CM to illustrate current perceptions about drivers and responses. The hierarchical CM generally increased in complexity from top to bottom. Among all rivers, there tended to be greater understanding of links from drivers to Tier 1 and Tier 2 EECs, and less understanding about linkages to Tier 3.

The strength of understanding of interactions between anthropogenic and natural drivers and EECs, and between and within EECs, varied considerably among river systems, however, resulting from both variable complexity and existing knowledge. For example, linkages from drivers to Tier 1 EECs were considered strong in the case of the Humpback Chub in the Grand Canyon, but between Tier 1 and Tier 2 only moderate. This is probably indicative of the substantial research investments in examination of physical processes in this river system [95].

Although we did not prescribe a specific approach to the CM process, the case studies employed similar strategies. Our modelling exercises started with the definition of a management

goal. In all our case studies, the management goals pertained to a desired biological endpoint represented in Tier 3. After the definition of the management goal, we conceptualized interactions between drivers and EECs and between EECs with a combination of top-down and bottom-up approaches. A top-to-bottom approach to working with these models is generally consistent with a management perspective wherein anthropogenic drivers that are most directly managed in a large-river system (e.g., land and water use, etc.) cascade from top to bottom through fluxes to physical and chemical habitats, and then to biological responses. While this is generally true for anthropogenic drivers, a notable exception to the top-to-bottom management approach would be that in the U.S., there are few actions currently directed at reducing emissions affecting climate [96] which is a natural driver in our CM. Climate was hypothesized to be a stressor in the case study application of the CM to Arkansas River Shiner management in the South Canadian River and is hypothesized to be affecting hydrologic regimes elsewhere [97, 98], but was not specifically mentioned in other case studies. The CMs can readily be modified to incorporate other factors or pathways (e.g., climate effects) as new information or perspectives become available. A bottom to top approach is equally or more valuable as it starts with the foundation of understanding about the species or community, and then seeks to identify which stressors affect population or community responses. A bottom-up approach can readily identify information gaps in linkages from ecological processes to demographic parameters [99].

The top-to-bottom and bottom-up approaches meet in the middle in Tier 2 in the concept of habitat: the resources and conditions present in an area that produce occupancy [100]. Tier 2 is critical as it has little value if it is not defined based on biological requirements or if managers lack understanding on how habitat is formed. Among our examples, the Upper Mississippi River is notable for asserting strong understanding of the linkages from land-use stressors to sediment

regime to diminished overwintering habitat for native adult fishes. After that, interactions with other processes and life stages combine to increase uncertainty about whether overwintering habitat is a limiting factor in biodiversity. In contrast, the high confidence in understanding how White Sturgeon egg quality and production are linked to spawning habitat in the Columbia River Basin, provides a strong linkage upward through Tier 1 EECs and potential management actions (Fig 7). Although at times elusive, the concept of habitat is critical for linking management to biotic endpoints [101].

Large rivers are typically managed for multiple objectives, including fisheries, multi-species, or ecosystem objectives. Management decisions typically require an understanding of how management actions propagate through a river ecosystem. Although the emphasis may be on a biological endpoint (among other objectives), understanding the intermediate steps and the processes linking them, and potential interactions between processes or EEC components, can help formulate effective management strategies; especially as multiple objectives compete. In a multi-species context, the conceptual models can help identify commonalities and differences in in how stressors propagate to biota and therefore provide a basis for prioritizing monitoring efforts. In the case where species or guilds have similar habitat affinities and life histories, a dominant anthropogenic stress pathway may be hypothesized and focus on a single or few monitoring components may be justified. An example may be multiple large-river species that are known to be cued to spawn by spring flow pulses. In such a case, the characteristics of the annual hydrograph would be a dominant physical monitoring variable and biological monitoring could focus on reproductive success of one or more of the species. In the case where multiple species of concern have different reproductive strategies – for example, rheophilic species like sturgeon that may require in-channel dispersion of young to flowing habitats compared to

invasive carp whose young thrive when they can disperse to lentic floodplain pools – pathways and monitoring strategies will diverge. In the latter case, it would probably not be sufficient to monitor and assess the characteristics of the annual hydrograph; instead, hydrologic metrics would need to be integrated with hydraulic and geomorphic metrics to assess where and when the different habitats would be available and could be targeted for young-of-the-year sampling. Effectively addressing multiple species would rely on detailed knowledge of life histories and how they play out on the landscape – such information is missing for many species and may need to be developed for effective design of monitoring and management actions.

Management actions intended to benefit fish in large rivers can directly or indirectly affect multiple ecosystem components. Without consideration of the effects of management on non-target ecosystem components, unintended consequences may limit management efficacy. Hypothesizing inter-tier interactions in the Tier 3 EEC (e.g., see Fig 7), can provide insight on the potential interactions among fish species and other biological components in the context of the hierarchical CM. In all our case studies, the lumping of multiple biological interactions in Tier 3 resulted in a simplification of complex trophic interactions. For example, as Tier 3 encompasses all biological responses, it includes multiple life stages of many interacting species at varying trophic levels. Because of this, the four CMs diverged significantly at Tier 3 as components were expanded to accommodate existing understanding. Even as the Tier 3 components were expanded in complexity, they remained highly simplified views of the ecosystem. Simplification was based, in part, on the importance of key species in management goals and the experts' existing knowledge. Even though the hypothesized Tier 3 interactions in our case studies conveyed a simplification of the trophic interactions, the hypothesized interactions do suggest the need for information that clarifies the trophic interactions and effects

of Tier 3 EEC components on the biological endpoint. If desired or warranted, the Tier 3 EEC could be elaborated to capture more complexity. For example, in Fig 7, the Tier 3 inter-tier interaction between anadromous and resident fishes and white sturgeon larvae could expanded to include interactions with specific fish species. Monitoring can help clarify the effects of management actions, including on non-target ecosystem components, but only if data are collected to characterize key ecosystem processes that could affect the outcome. The process of considering and elucidating Tier 3 EEC interactions can help identify the non-target ecosystem components that could be affected when managing for a specific biological endpoint.

The CMs explored here also provide a framework for considering return on science investments. The knowledge needed for effective management of large rivers can be gained by monitoring intermediate endpoints along the cascade, but the type of information and costs vary widely. Costs for monitoring Tier 1 EECs can be high but some programs are already in place. For example, large rivers are likely to have monitoring infrastructure installed for Tier 1 monitoring of discharge and temperature regimes, with varying potential for monitoring sediment transport and water quality. Investment at Tier 2 may emphasize physical processes and habitats that can be measured at relatively low cost, assuming that habitats are adequately defined based on biological criteria. In larger rivers, Tier 2 habitat assessments can be more cost effective compared to smaller rivers because they can rely on automated data collection through hydroacoustics and remote sensing [101]. As discussed above, habitat assessments have value only to the extent that they are based on well-defined biological requirements; it is notable that some large-river management efforts have found that relatively simple habitat models are useful to predict biological responses [102]. At Tier 3, costs can increase substantially because of structural uncertainties (i.e., which life stages, which species are most important to monitor) and

because of the inherent uncertainties of monitoring fish in large river systems where detection probability can be low and highly variable [36, 103]. Generally, the cost of monitoring increases from Tier 1 to Tier 3 in the CM hierarchy; at the same time, the relevance of information to decision making is typically greater for biological responses depicted in Tier 3 [104].

Because both costs and information benefits increase from Tier 1 to Tier 3 in the CM hierarchy, it is difficult to generalize about where the benefit:cost ratio would be optimized. Indeed, as discussed by Jacobson and Berkley [30], the decision about where in the hierarchy monitoring resources would get the highest return on investment may depend more directly on managers' and stakeholders' perceptions about risks of acting with incomplete information. For example, the details of how a fish's reproductive strategy depends on the nuances of a seasonal hydrograph may not be known, but stakeholders may believe strongly that the natural hydrograph was functional for the species and therefore monitoring of the flow regime will have the highest return on investment and, by extension, restoration of the flow regime is likely to have the most positive effects. On the other hand, in systems where stakeholders opinions are divided or socio-economic values would be compromised by a return to a natural flow regime, managers may be required to demonstrate more precisely how elements of the flow regime propagate to species' benefits [105]. Thus, once information needs are identified and there is an assessment of the availability of data identified as information needs, there needs to be a process whereby the costs of collecting the information need to be placed in a socioeconomic context (e.g., see [30]).

The development of the CMs described in this manuscript can be a first step in application of structured decision-making (SDM) and its iterative form-adaptive management (AM) processes [106, 107]. Structured decision-making is a stakeholder driven process by which

a problem can be defined with conceptual models and decomposed into decision components that include the problem context, stakeholder objectives, potential management actions, consequences of those actions on the objectives, and trade-offs related to different decisions (actions) [107-109]. One primary focus of SDM is the identification of uncertainties such as those identified in the CMs for the case studies in this paper [110]. Quantification of the influence of decision relevant uncertainties can be modeled using sensitivity analysis and other techniques and ranked [107, 109, 111]. In addition, the quantitative techniques available to assist in solving complex ecological problems are robust and range in complexity from consequences tables to Bayesian models to dynamic optimization models [107-109, 112, 113]. The SDM process is often used as the set-up phase for adaptive management which includes monitoring over time to reduce uncertainty related to how management will influence important outcomes (e.g. fish population status; [109, 112].

The CM may also help to identify which processes or components are amenable to a field monitoring effort and which are more aptly addressed through laboratory or mesocosm experiments. For example, if it is hypothesized that the condition of age 1+ Arkansas Shiners is a critical determining factor in egg quality or production (Fig 4), it could be determined that the best approach to developing a quantitative relation between condition and eggs is through a controlled laboratory experiment rather than field-based monitoring. The CM helps to visualize where different types of information may be applied within a decision-making framework.

A large-river CM may also serve as a precursor to computational ecological or population models [30]. Similar questions about how monitoring and other science efforts should be distributed among EECs and processes can be addressed iteratively by carrying out sensitivity analyses in a modeling framework. Indeed, given substantial uncertainties associated with

monitoring data, computation modeling can be considered a necessary component of large-river monitoring and evaluation systems [99, 114].

Conclusions

We found the process of conceptualizing the relationships between and within EECs fostered a critical assessment of what we know about factors affecting the management endpoint. By visualizing how EEC drivers directly and indirectly affect management endpoints, our CM identified critical information gaps and uncertainties that, if resolved, could improve our understanding of how to best meet management objectives. The process of conceptualizing the EEC relationships affecting fish in large rivers could help to structure, or restructure, monitoring programs around scientifically sound monitoring questions, promote the selection of relevant ecological indicators that characterize resource condition or management outcomes, and facilitate communication and information sharing within and between organizations managing or researching management endpoints. Ultimately, understanding the mechanisms by which EECs influence large-river fishes will improve the effectiveness of restoration and management actions.

As shown with our case studies, our CM is flexible and applicable to a wide range of river systems with different anthropogenic drivers and management objectives. We feel our CM provides a generic structure that scientists can adapt to their management goals and needs. By not being overly prescriptive, for example, with respect to the components of the Tier 2 and 3 EEC components, scientists can adapt the CM to different biological communities and

management endpoints. By doing so, we feel that users have the flexibility to place their management questions in the context of EECs that are specific to their large-river system.

Although the case studies addressed management issues that were river or basin specific, there were similarities relative to information needs and data availability. For example, in most systems information on river discharge and water temperature were needed and available. Conversely, information regarding trophic relationships and the habitat requirements of larval fishes were generally lacking. This result suggests that there may be a common need for a better understanding of certain factors across large-river systems.

Acknowledgements

We thank Megan Dethloff of the Pacific Northwest Aquatic Monitoring Partnership for her role in organizing the logistics associated with the workshop and conference calls needed to develop this manuscript. David Ward from the U.S. Geological Survey's Grand Canyon Monitoring and Research Center provided many valuable comments and suggestions that improved the manuscript. This work was funded in part by U.S. Geological Survey, Science Synthesis, Analysis, and Research Program. This research is a contribution of the Ball State University, Illinois Natural History Survey, Oregon Department of Fish and Wildlife, Pacific Northwest Aquatic Monitoring Partnership, the Alabama Cooperative Fish and Wildlife Research Unit, and the U.S. Geological Survey Columbia Environmental Research Center, Grand Canyon Monitoring and Research Center, Oregon Water Science Center, Upper Midwest Environmental Sciences Center, and Western Fisheries Research Center. Any use of trade, firm, or product

names is for descriptive purposes only and does not imply endorsement by the U.S. Government. An animal care and use protocol was not required for this research.

References

1. Thomas JA, Emery EB, McCormick FH. Detection of temporal trends in Ohio River fish assemblages based on lockchamber surveys (1957–2001). In: J.N. Rinne, R.M. Hughes, Calamusso B, editors. Historical changes in large river fish assemblages of the Americas. 45. Bethesda, MD: American Fisheries Society; 2005. p. 431-49.
2. Lohner TW, Dixon DA. The value of long-term environmental monitoring programs: an Ohio River case study. Environ Monit Assess. 2013;185(11):9385-96.
3. McClelland MA, Sass GG, Cook TR, Irons KS, Michaels NN, O'Hara TM, et al. The long-term Illinois River fish population monitoring program. Fisheries. 2012;37(8):340-50. doi: 10.1080/03632415.2012.704815. .
4. Ward JV. Riverine landscapes: Biodiversity patterns, disturbance regimes, and aquatic conservation. Biol Conserv. 1998;83(3):269-78. PubMed PMID: WOS:A1998YJ19500005.
5. Lee LS, Garnett JA, Bright EG, Sharitz RR, Batzer DP. Vegetation, invertebrate, and fish community response to past and current flow regulation in floodplains of the Savannah River, Southeastern USA. Wetlands Ecol Manage. 2016;24:443-55. doi: 10.1007/s11273-015-9470-y.
6. Lowe WH, Likens GE, Power ME. Linking scales in stream ecology. Bioscience. 2006;56(7):591-7. PubMed PMID: WOS:000239017800010.
7. Fausch KD, Torgersen CE, Baxter CV, Li HW. Landscapes to riverscapes: Bridging the gap between research and conservation of stream fishes. Bioscience. 2002;52(6):483-98. PubMed PMID: WOS:000176233800009.
8. Allan JD. Landscapes and riverscapes: The influence of land use on stream ecosystems. Annu Rev Ecol Evol S. 2004;35:257-84. PubMed PMID: WOS:000226244100010.
9. Steel EA, Hughes RM, Fullerton AH, Schmutz S, Young JA, Fukushima M, et al. Are we meeting the challenges of landscape-scale riverine research? A review. Living Reviews in Landscape Research. 2010;4.
10. Thoms M, Sheldon F. Large rivers as complex adaptive ecosystems. River Res Appl. 2019;35(5):451-8. doi: 10.1002/rra.3448. PubMed PMID: WOS:000470931600001.
11. Nilsson C, Reidy CA, Dynesius M, Revenga C. Fragmentation and Flow Regulation of the World's Large River Systems. Science. 2005;15:405-8.
12. Harding JS, Benfield EF, Bolstad PV, Helfman GS, Jones EBD. Stream biodiversity: The ghost of land use past. P Natl Acad Sci USA. 1998;95(25):14843-7. PubMed PMID: WOS:000077436700047.
13. King AJ, Gawne B, Beesley L, Koehn JD, Nielsen DL, Price A. Improving Ecological Response Monitoring of Environmental Flows Environmental Management. 2015;55:991-1005.
14. Gawne B, Hale J, Stewardson MJ, Webb JA, Ryder DS, Brooks SS, et al. Monitoring of environmental flow outcomes in a large river basin: The Commonwealth Environmental Water Holder's long-term intervention in the Murray-Darling Basin, Australia. River Res Appl. 2020;36(4):630-44. doi: 10.1002/rra.3504. PubMed PMID: WOS:000484205300001.
15. Schletterer M, Shaporenko SI, Kuzovlev VV, Minin AE, Van Geest GJ, Middelkoop H, et al. The Volga: Management issues in the largest river basin in Europe. River Res Appl. 2019;35(5):510-9. doi: 10.1002/rra.3268. PubMed PMID: WOS:000470931600007.
16. Lindenmayer DB, Likens GE. The science and application of ecological monitoring. Biol Conserv. 2010;143(6):1317-28. PubMed PMID: WOS:000278572300002.

17. Worthington TA, Brewer SK, Grabowski TB, Mueller J. Backcasting the decline of a vulnerable Great Plains reproductive ecotype: Identifying threats and conservation priorities. Global Change Biology. 2014;20:89-102.

18. Worthington TA, Echelle AA, Perkin JS, Mollenhauer R, Farless N, Dyer JJ, et al. The emblematic minnows of the North American Great Plains: A synthesis of threats and conservation opportunities. Fish and Fisheries. 2018;19(2):271-307. doi: 10.1111/faf.12254. PubMed PMID: WOS:000426503000006.

19. Noon BR, Spies TA, Raphael MG. Conceptual basis for designing an effectiveness monitoring plan. The strategy and designing of the effectiveness program for the Northwest Forest Plan1999. p. 21-48.

20. Fancy SG, Gross JE, Carter SL. Monitoring the condition of natural resources in US national parks. Environ Monit Assess. 2009;151(1-4):161-74. PubMed PMID: WOS:000263832800014.

21. Irvine KM, Miller SW, Al-Chokhachy RK, Archer EK, Roper BB, Kershner JL. Empirical evaluation of the conceptual model underpinning a regional aquatic long-term monitoring program using causal modelling. Ecol Indic. 2015;50:8-23. PubMed PMID: WOS:000348265200002.

22. Hein T, Funk A, Pletterbauer F, Graf W, Zsuffa I, Haidvogl G, et al. Management challenges related to long-term ecological impacts, complex stressor interactions, and different assessment approaches in the Danube River Basin. River Res Appl. 2019;35(5):500-9. doi: 10.1002/rra.3243. PubMed PMID: WOS:000470931600006.

23. U.S. Columbia Basin Tribes and Canadian First Nations. Fish passage and reintroduction into the US and Canadian upper Columbia River. *An Interim Joint Paper of the US Columbia Basin Tribes and Canadian First Nations February*. 2014.

24. Carlisle DM, Meador MR, Short TM, Tate CM, Gurtz ME, Bryant Jr WL, et al. The quality of our Nation’s waters--ecological health in the Nation's streams, 1993-2005. US Geological Survey Circular 1391. 2013.

25. Sedeño-Díaz JE, López-López E. Freshwater fish as sentinel organisms: From the molecular to the population level, a review. In: Turker H, editor. New Advances and Contributions to Fish Biology: IntechOpen; 2012.

26. Agency USEP. Rivers and streams assessment 2008-2009 A collaborative survey DRAFT (EPA/841/D-13/001). Washington, DC Office of Wetlands, Oceans and Watersheds Office of Research and Development.; 2013.

27. DeBoer JA, Thoms MC, Casper AF, Delong MD. The Response of Fish Diversity in a Highly Modified Large River System to Multiple Anthropogenic Stressors. J Geophys Res-Biogeo. 2019;124(2):384-404. doi: 10.1029/2018jg004930. PubMed PMID: WOS:000460859100013.

28. Collier KJ, Baker C, David BO, Gorski K, Pingram MA. Linking ecological science with management outcomes on New Zealand's longest river. River Res Appl. 2019;35(5):476-88. doi: 10.1002/rra.3181. PubMed PMID: WOS:000470931600004.

29. Harwell MA, Myers V, Young T, Bartuska A, Gassman N, Gentile JH, et al. A framework for an ecosystem integrity report card. Bioscience. 1999;49(7):543-56. PubMed PMID: WOS:000081211600008.

30. Jacobson RB, Berkley J. Conceptualizing and communicating ecological river restoration. In: Simon A, Bennett SJ, Castro JM, editors. Stream Restoration in Dynamic Fluvial

Systems. Geophysical Monograph Series. Washington, D.C.: American Geophysical Union; 2011. p. 9-27.

31. Ward DL, Casper AF, Counihan TD, Bayer JM, Waite IR, Kosovich JJ, et al. Long-Term Fish Monitoring in Large Rivers: Utility of "Benchmarking" across Basins. Fisheries. 2017;42(2):100-14. PubMed PMID: WOS:000394751100007.

32. Counihan TD, Waite IR, Casper AF, Ward DL, Sauer JS, Irwin ER, et al. Can data from disparate long-term fish monitoring programs be used to increase our understanding of regional and continental trends in large river assemblages? Plos One. 2018;13(1). PubMed PMID: WOS:000423412500050.

33. Carpenter SR, Bennett EM, Peterson GD. Special feature on scenarios for ecosystem services. Ecol Soc. 2006;11(2). PubMed PMID: WOS:000243280800002.

34. Tonn WM. Climate Change and Fish Communities - a Conceptual-Framework. Transactions of the American Fisheries Society. 1990;119(2):337-52. PubMed PMID: WOS:A1990DV01100015.

35. Rosenfeld J. Assessing the habitat requirements of stream fishes: An overview and evaluation of different approaches. Transactions of the American Fisheries Society. 2003;132(5):953-68. PubMed PMID: WOS:000185446500011.

36. Mollenhauer R, Brewer SK. Multinomial N-Mixture Models Improve the Applicability of Electrofishing for Developing Population Estimates of Stream-Dwelling Smallmouth Bass. North American Journal of Fisheries Management. 2017;37(1):211-24. PubMed PMID: WOS:000394648700021.

37. Grill G, Lehner B, Lumsdon AE, MacDonald GK, Zarfl C, Liermann CR. An index-based framework for assessing patterns and trends in river fragmentation and flow regulation by global dams at multiple scales. Environmental Research Letters. 2015;10(1):015001.

38. van Puijenbroek PJ, Buijse AD, Kraak MH, Verdonschot PF. Species and river specific effects of river fragmentation on European anadromous fish species. River Res Appl. 2019;35(1):68-77.

39. Wolter C, Buijse AD, Parasiewicz P. Temporal and Spatial Patterns of Fish Response to Hydromorphological Processes. River Res Appl. 2016;32(2):190-201. doi: 10.1002/rra.2980. PubMed PMID: WOS:000369979000005.

40. Counihan TD, Chapman CG. Relating river discharge and water temperature to the recruitment of age-0 White Sturgeon (Acipenser transmontanus Richardson, 1836) in the Columbia River using over-dispersed catch data. J Appl Ichthyol. 2018;34(2):279-89. PubMed PMID: WOS:000428797200004.

41. Halvorson LJ, Cady BJ, Kappenman KM, James BW, Webb MAH. Observations of handling trauma of Columbia River adult white sturgeon, Acipenser transmontanus Richardson, 1836, to assess spawning sanctuary success. J Appl Ichthyol. 2018;34(2):390-7.

42. Jay DA, Naik PK. Distinguishing human and climate influences on hydrological disturbance processes in the Columbia River, USA. Hydrolog Sci J. 2011;56(7):1186-209. doi: 10.1080/02626667.2011.604324. PubMed PMID: WOS:000297289600008.

43. Omernik JM, Griffith GE, Hughes RM, Glover JB, Weber MH. How Misapplication of the Hydrologic Unit Framework Diminishes the Meaning of Watersheds. Environmental Management. 2017;60(1):1-11. doi: 10.1007/s00267-017-0854-z. PubMed PMID: WOS:000402792800001.

44. U.S. Geological Survey, U.S. Department of Agriculture - Natural Resources Conservation Service. Federal Standards and Procedures for the National Watershed Boundary Dataset (WBD) (4 ed.). U.S. Geological Survey Techniques and Methods 11–A3; 2013. p. 63.

45. Frissell CA, Liss WJ, Warren CE, Hurley MD. A Hierarchical Framework for Stream Habitat Classification - Viewing Streams in a Watershed Context. Environmental Management. 1986;10(2):199-214. doi: Doi 10.1007/Bf01867358. PubMed PMID: WOS:A1986A793400006.

46. Simenstad CA, Burke JL, O'Connor JE, Cannon C, Heatwole DW, Ramirez MF, et al. Columbia River Estuary Ecosystem Classification—Concept and Application. U.S. Geological Survey Open-File Report 2011-1228; 2011. p. 54.

47. Levin SA, Paine RT. Disturbance, patch formation, and community structure. P Natl Acad Sci USA. 1974;71(7):2744-7.

48. Pringle CM, Naiman RJ, Bretschko G, Karr JR, Oswood MW, Webster JR, et al. Patch Dynamics in Lotic Systems - the Stream as a Mosaic. J N Am Benthol Soc. 1988;7(4):503-24. doi: Doi 10.2307/1467303. PubMed PMID: WOS:A1988R878400011.

49. Worthington TA, Brewer SK, Farless N, Grabowski TB, Gregory MS. Interacting Effects of Discharge and Channel Morphology on Transport of Semibuoyant Fish Eggs in Large, Altered River Systems. Plos One. 2014;9(5). doi: 10.1371/journal.pone.0096599. PubMed PMID: WOS:000338029800091.

50. Wilde GR. Migration of Arkansas River Shiner and other Broadcast Spawning Fishes in the Canadian River, New Mexico-Texas. Albuquerque, New Mexico2016.

51. Hoagstrom CW, Turner TF. Recruitment ecology of pelagic-broadcast spawning minnows: paradigms from the ocean advance science and conservation of an imperilled freshwater fauna. Fish and Fisheries. 2015;16(2):282-99. doi: 10.1111/faf.12054. PubMed PMID: WOS:000352536200005.

52. Birken AS, Cooper DJ. Processes of Tamarix invasion and floodplain development along the lower Green River, Utah. Ecol Appl. 2006;16(3):1103-20. doi: Doi 10.1890/1051-0761(2006)016[1103:Potiaf]2.0.Co;2. PubMed PMID: WOS:000238451500023.

53. Nagler PL, Glenn EP, Jarnevich CS, Shafroth PB. Distribution and Abundance of Saltcedar and Russian Olive in the Western United States. Crit Rev Plant Sci. 2011;30(6):508-23. doi: 10.1080/07352689.2011.615689. PubMed PMID: WOS:000298323800002.

54. Wilde GR, Bonner TH, Zwank PJ. Diets of the Arkansas River shiner and peppered chub in the Canadian River, new Mexico and Texas. J Freshwater Ecol. 2001;16(3):403-10. doi: Doi 10.1080/02705060.2001.9663829. PubMed PMID: WOS:000171102500011.

55. Shafer M, Ojima D, Antle JM, Kluck D, McPherson RA, Petersen S, et al. Ch. 19: Great Plains. In: Melillo JM, Richmond TC, Yohe GW, editors. Climate Change Impacts in the United States: The Third National Climate Assessment: U.S. Global Change Research Program; 2014. p. 441-61.

56. Otkin JA, Anderson MC, Hain C, Svoboda M, Johnson D, Mueller R, et al. Assessing the evolution of soil moisture and vegetation conditions during the 2012 United States flash drought. Agr Forest Meteorol. 2016;218:230-42. doi: 10.1016/j.agrformet.2015.12.065. PubMed PMID: WOS:000370905100022.

57. Worthington TA, Zhang TJ, Logue DR, Mittelstet AR, Brewer SK. Landscape and flow metrics affecting the distribution of a federally-threatened fish: Improving management, model fit, and model transferability. Ecol Model. 2016;342:1-18. doi: 10.1016/j.ecolmodel.2016.09.016. PubMed PMID: WOS:000388154100001.

58. Gorman OT, Stone DM. Ecology of spawning humpback chub, Gila cypha, in the Little Colorado River near Grand Canyon, Arizona. Environ Biol Fish. 1999;55(1-2):115-33. PubMed PMID: WOS:000081503900011.
59. Childs MR, Clarkson RW, Robinson AT. Resource use by larval and early juvenile native fishes in the Little Colorado River, Grand Canyon, Arizona. Transactions of the American Fisheries Society. 1998;127(4):620-9. PubMed PMID: WOS:000075350600009.
60. Converse YK, Hawkins CP, Valdez RA. Habitat relationships of subadult humpback chub in the Colorado River through Grand Canyon: Spatial variability and implications of flow regulation. Regul River. 1998;14(3):267-84. PubMed PMID: WOS:000074199700004.
61. Petersen JH, Paukert CP. Development of a bioenergetics model for humpback chub and evaluation of water temperature changes in the Grand Canyon, Colorado River. Transactions of the American Fisheries Society. 2005;134(4):960-74. PubMed PMID: WOS:000230411700015.
62. Marsh PC, Douglas ME. Predation by introduced fishes on endangered humpback chub and other native species in the Little Colorado River, Arizona. Transactions of the American Fisheries Society. 1997;126(2):343-6. PubMed PMID: WOS:A1997WW21700018.
63. Page LM, Burr BM. A field guide to freshwater fishes of North America north of Mexico. Boston, MA: Houghton Mifflin Harcourt; 2011.
64. Quinn TP, Adams DJ. Environmental changes affecting the migratory timing of American shad and sockeye salmon. Ecology. 1996;77(4):1151-62. PubMed PMID: WOS:A1996UN89400018.
65. Parsley MJ, Beckman LG. White sturgeon spawning and rearing habitat in the lower Columbia River. North American Journal of Fisheries Management. 1994;14:812-27.
66. Chapman CG, Jones TA. First Documented Spawning of White Sturgeon in the Lower Willamette River, Oregon. Northwest Sci. 2010;84(4):327-35. PubMed PMID: WOS:000285797800002.
67. North JA, Beamesderfer RC, Rien TA. Distribution and Movements of White Sturgeon in 3 Lower Columbia River Reservoirs. Northwest Sci. 1993;67(2):105-11. PubMed PMID: WOS:A1993LE90500006.
68. Perrin CJ, Rempel LL, Rosenau ML. White sturgeon spawning habitat in an unregulated river: Fraser River, Canada. Transactions of the American Fisheries Society. 2003;132(1):154-65. PubMed PMID: WOS:000180362000016.
69. Beamesderfer RCP, Rien TA, Nigro AA. Differences in the Dynamics and Potential Production of Impounded and Unimpounded White Sturgeon Populations in the Lower Columbia River. Transactions of the American Fisheries Society. 1995;124(6):857-72. PubMed PMID: WOS:A1995TF91800007.
70. Feist GW, Webb MAH, Gundersen DT, Foster EP, Schreck CB, Maule AG, et al. Evidence of detrimental effects of environmental contaminants on growth and reproductive physiology of white sturgeon in impounded areas of the Columbia River. Environ Health Persp. 2005;113(12):1675-82. PubMed PMID: WOS:000233713200035.
71. Sanderson BL, Barnas KA, Rub AMW. Nonindigenous Species of the Pacific Northwest: An Overlooked Risk to Endangered Salmon? Bioscience. 2009;59(3):245-56. PubMed PMID: WOS:000263863400009.
72. Zimmerman MP. Food habits of smallmouth bass, walleyes, and northern pikeminnow in the lower Columbia River Basin during outmigration of juvenile anadromous salmonids.

Transactions of the American Fisheries Society. 1999;128(6):1036-54. PubMed PMID: WOS:000089184000005.
73. Knox JC. Agricultural influence on landscape sensitivity in the Upper Mississippi River Valley. Catena. 2001;42(2-4):193-224.
74. Turner RE, Rabalais NN. Linking landscape and water quality in the Mississippi River Basin for 200 years. Bioscience. 2003;53(6):563-72.
75. Houser JN, Richardson WB. Nitrogen and phosphorus in the Upper Mississippi River: transport, processing, and effects on the river ecosystem. Hydrobiologia. 2010;640(1):71-88.
76. Schilling KE, Chan KS, Liu H, Zhang YK. Quantifying the effect of land use land cover change on increasing discharge in the Upper Mississippi River. Journal of Hydrology. 2010;387(3-4):343-5.
77. Bouska KL. Discontinuities and functional resilience of large river fish assemblages. Ecosphere. 2018;9(7). doi: 10.1002/ecs2.2351. PubMed PMID: WOS:000441526900003.
78. Pendleton RM, Schwinghamer C, Solomon LE, Casper AF. Competition among river planktivores: are native planktivores still fewer and skinnier in response to the Silver Carp invasion? Environ Biol Fish. 2017;100(10):1213-22. doi: 10.1007/s10641-017-0637-7. PubMed PMID: WOS:000412840700005.
79. Solomon LE, Pendleton RM, Chick JH, Casper AF. Long-term changes in fish community structure in relation to the establishment of Asian carps in a large floodplain river. Biol Invasions. 2016;18(10):2883-95. doi: 10.1007/s10530-016-1180-8. PubMed PMID: WOS:000382847100012.
80. Bhowmik NG, Demissie M. Sedimentation in the Illinois River valley and backwater lakes. Journal of Hydrology. 1989;105(1-2):187-95.
81. WEST Consultants Inc. Upper Mississippi River and Illinois Waterway Cumulative Effects Study Rock Island, Illinois, USA: Department of the Army, Corps of Engineers, 2000.
82. Nannini MA, Goodrich J, Dettmers JM, Soluk DA, Wahl DH. Larval and early juvenile fish dynamics in main channel and backwater lake habitats of the Illinois River ecosystem. Ecol Freshw Fish. 2012;21(4):499-509. PubMed PMID: WOS:000308290000004.
83. Love SA, Phelps QE, Tripp SJ, Herzog DP. The Importance of Shallow-Low Velocity Habitats to Juvenile Fish in the Middle Mississippi River. River Res Appl. 2017;33(3):321-7. PubMed PMID: WOS:000397876900002.
84. Eckblad JW, Volden CS, Weilgart LS. Allochthonous drift from backwaters to the main channel of the Mississippi River. The American Midland Naturalist. 1984;111(1):16-22.
85. Sheaffer WA, Nickum JG. Relative abundance of macroinvertebrates found in habitats associated with backwater area confluences in Pool 13 of the Upper Mississippi River. Hydrobiologia. 1986;136:113-20.
86. Wahl DH, Goodrich J, Nannini MA, Dettmers JM, Soluk DA. Exploring riverine zooplankton in three habitats of the Illinois River ecosystem: Where do they come from? Limnology and Oceanography. 2008;53(6):2583-93.
87. Burdis RM, Hoxmeier RJH. Seasonal zooplankton dynamics in main channel and backwater habitats of the Upper Mississippi River. Hydrobiologia. 2011;667:69-87.
88. Ochs CA, Pongruktham O, Zimba PV. Darkness at the break of noon: Phytoplankton production in the Lower Mississippi River. Limnology and Oceanography. 2013;58(2):555-68.

89. Sheehan RJ, Lewis WM, Bodensteiner LR. Winter habitat requirements and overwintering of riverine fishes. Online: http://opensiuc.lib.siu.edu/fiaq_reports/8: Southern Illinois University, 1994.
90. Raibley PT, Irons KS, O'Hara TM, Blodgett KD, Sparks RE. Winter habitats used by largemouth bass in the Illinois River, a large river-floodplain ecosystem. North American Journal of Fisheries Management. 1997;17(2):401-12.
91. Hurley KL, Sheehan RJ, Heidinger RC, Wills PS, Clevenstine B. Habitat use by Middle Mississippi River pallid sturgeon. Transactions of the American Fisheries Society. 2004;133:1033-41.
92. Schwartz JS, Herricks EE. Fish use of stage-specific fluvial habitats as refuge patches during a flood in a low-gradient Illinois stream. Canadian Journal of Fisheries and Aquatic Sciences. 2005;62:1540-52.
93. Junk WJ, Bayley PB, Sparks RE. The flood pulse concept in river-floodplain systems. Canadian special publication of fisheries and aquatic sciences. 1989;106(1):110-27.
94. Sparks RE. Need for ecosystem management of large rivers and their floodplains. Bioscience. 1995;45(3):168-82. PubMed PMID: WOS:A1995QH75600010.
95. Melis TS, Walters CJ, Korman J. Surprise and Opportunity for Learning in Grand Canyon: the Glen Canyon Dam Adaptive Management Program. Ecol Soc. 2015;20(3). doi: Artn 2210.5751/Es-07621-200322. PubMed PMID: WOS:000362913100013.
96. Millar RJ, Fuglestvedt JS, Friedlingstein P, Rogelj J, Grubb MJ, Matthews HD, et al. Emission budgets and pathways consistent with limiting warming to 1.5 C. Nature Geoscience. 2017;10(10):741-7.
97. Shkolnik I, Pavlova T, Efimov S, Zhuravlev S. Future changes in peak river flows across northern Eurasia as inferred from an ensemble of regional climate projections under the IPCC RCP8. 5 scenario. Climate dynamics. 2018;50:215-30.
98. Fonseca AR, Santos JA. Predicting hydrologic flows under climate change: The Tâmega Basin as an analog for the Mediterranean region. Science of the total environment. 2019;668:1013-24.
99. Jacobson RB, Annis ML, Colvin ME, James DA, Welker TL, Parsley MJ. Missouri River *Scaphirhynchus albus* (pallid sturgeon) effects analysis—Integrative report 2016. U.S. Geological Survey Scientific Investigations Report 2016-5064. Reston, VA.2016. p. 154.
100. Hall LS, Krausman PR, Morrison ML. The habitat concept and a plea for standard terminology. Wildlife Soc B. 1997;25(1):173-82. PubMed PMID: WOS:A1997XA82200031.
101. Fisher WL, Bozek MA, Vokoun JC, Jacobson RB. Freshwater aquatic habitat measurements. In: A. V. Zale, D. L. Parrish, Sutton T, editors. Fisheries Techniques, 3rd edition. Bethesda, Maryland: American Fisheries Society; 2012. p. 101-61.
102. Lamouroux N, Gore JA, Lepori F, Statzner B. The ecological restoration of large rivers needs science-based, predictive tools meeting public expectations: an overview of the Rhone project. Freshwater Biol. 2015;60(6):1069-84. doi: 10.1111/fwb.12553. PubMed PMID: WOS:000354254800001.
103. Schloesser JT, Paukert CP, Doyle WJ, Hill TD, Steffensene KD, Travnichek VH. Fish Assemblages at Engineered and Natural Channel Structures in the Lower Missouri River: Implications for Modified Dike Structures. River Res Appl. 2012;28(10):1695-707. doi: 10.1002/rra.1578. PubMed PMID: WOS:000312651000008.

104. Jacobson RB, Berkley J. Conceptualizing and communicating ecological river restoration. In: Simon A, Bennet S, Castro J, editors. Stream Restoration in Dynamic Fluvial Systems: Scientific Approaches, Analyses and Tools: AGU Geophysical Monograph 194; 2011. p. 9-28.

105. Jacobson RB, Galat DL. Design of a naturalized flow regime-an example from the Lower Missouri River, USA. Ecohydrology: Ecosystems, Land and Water Process Interactions, Ecohydrogeomorphology. 2008;1(2):81-104. PubMed PMID: WOS:000260000500001.

106. Rudnick DT, Ortner PB, Browder JA, Davis SM. A conceptual ecological model of Florida Bay. Wetlands Ecol Manage. 2005;25:870-83.

107. Conroy MJ, Peterson JT. Decision making in natural resources—A structured, adaptive approach. Oxford, United Kingdom: Wiley-Blackwell; 2013.

108. Gregory R, Failing L, Harstone M, Long G, McDaniels T, Ohlson D. Structured decision making—A practical guide to environmental management choices. Chichester, West Sussex, United Kingdom: John Wiley & Sons; 2012.

109. Irwin ER, Freeman M, Peterson J, Kennedy KDM, Lloyd MC, Ouellette Coffman KM, et al. Adaptive management of flows from R.L. Harris Dam (Tallapoosa River, Alabama)—Stakeholder process and use of biological monitoring data for decision making: U.S. Geological Survey Open-File Report 2019–1026. Reston, VA: U.S. Geological Survey; 2019. p. 93.

110. Martin J, Runge MC, Nichols JD, Lubow BC, Kendall WL. Structured decision making as a conceptual framework to identify thresholds for conservation and management. Ecol Appl. 2009;19(5):1079-90.

111. Chan TU, Hart BT, Kennard MJ, Pusey BJ, Shenton W, Douglas MM, et al. Bayesian network models for environmental flow decision making in the Daly River, Northern Territory, Australia. River Res Appl. 2012;28(3):283-301.

112. Williams BK, Szaro RC, Shapiro CD. Adaptive management—The U.S. Department of the Interior technical guide. In: Interior USDot, editor. Washington, D.C.2007. p. 72.

113. McGowan CP, Smith DR, Nichols JD, Lyons JE, Sweka J, Kalasz K, et al. Implementation of a framework for multi-species, multi-objective adaptive management in Delaware Bay. Biol Conserv. 2015;191:759-69.

114. Walters C, Korman J, Stevens LE, Gold B. Ecosystem modeling for evaluation of adaptive management policies in the Grand Canyon. Conserv Ecol. 2000;4(2):art. no.-1. PubMed PMID: WOS:000167492900002.

115. U.S. Geological Survey. Graphical Fisheries Database Browser - Stratified Random Sampling 2015 [cited 2018 September 9]. Available from: https://umesc.usgs.gov/data_library/fisheries/graphical/fish_front.html.

Supporting Information Captions

Fig S1. Discharge (m^3/s) patterns over time in the Canadian River, Oklahoma near Canadian, TX (USGS 2019; USGS Gage 07228000).

Fig S2. Hydrograph showing pre- and post-Glen Canyon Dam closure in 1964 (dashed line) mean monthly discharge (m^3/s), which transitions from seasonally stochastic to a more homogeneous regime focusing on anthropogenic interests.

Fig S3. The spatial and temporal scales of the management goal, the scientific inferences needed to inform the management goal, and that data collection needs to occur to support the inferences for monitoring information needs identified as requiring additional data in the case study addressing Humpback Chub recruitment in the Colorado River between Glen Canyon Dam and Lake Mead, Arizona (see Table S2 for additional detail). A:Tier 2 EEC= larval Humpback Chub habitat; Stressors=habitat fragmentation, turbidity and Tier 2 EEC= Humpback Chub spawning habitat; Stressor= water temperature and Tier 3 EEC=insect production; Stressor=benthic macroinvertebrate habitat quantity and quality and Tier 3 EECs=all; Inter-tier interaction=trophic level interactions; B:Tier 3 EEC=larval Humpback Chub production; Stressor= larval Humpback chub habitat quantity and quality; C: Tier 3 EEC=Humpback Chub egg quality and production; Stressors= Humpback Chub spawning habitat quantity and quality; D:Larval Humpback Chub production; Inter-tier interaction=mortality of Humpback Chub eggs; E:Tier 3 EEC= larval Humpback Chub production; Stressor=predation by invasive species and Tier 3 EEC=Primary production; Stressor=nutrient flux; F:Tier 1 EEC= biogeochemistry/thermodynamics; Inter-tier interaction=sediment adsorption of contaminants and nutrients; G:Tier 3 EEC=Humpback Chub age-0 recruitment; Inter-tier interaction=mortality of larval Humpback Chub.

Fig S4. Proportion of total annual Columbia River discharge at The Dalles, OR occurring in the month of June from 1879 to 2015.

Fig S5. The spatial and temporal scales of the management goal, the scientific inferences needed to inform the management goal, and that data collection needs to occur to support the inferences for monitoring information needs identified as requiring additional data in the case study addressing White Sturgeon recruitment in the Columbia River (see Table S3 for additional detail). A:Tier 1 EEC= biogeochemistry/thermodynamic; Stressor= altered biogeochemical regime and Tier 2 EEC= benthic macroinvertebrate habitat; Stressors=channel stability, sediment deposition, fragmentation and Tier 2 EEC=Larval White Sturgeon habitat; Stressors=habitat fragmentation sediment deposition, water temperature and Tier 2 EEC=White Sturgeon spawning habitat; Stressors=contaminants, sediment deposition and Tier 3 EEC=White Sturgeon egg quality and production; Stressor=predation by invasive species and Tier 3 EEC=White Sturgeon larvae production; Stressor=predation by invasive species and Tier 3 EEC=benthic macroinvertebrate production; Stressor=benthic macroinvertebrate habitat quantity and quality; B:Tier 1 EEC=sediment transport; Stressors=altered sediment regime, altered hydraulic regime and Tier 1 EECs=channel morphology/hydraulics, sediment transport; Stressor=altered hydraulic regime and Tier 3 EEC=primary production; Stressor=nutrient fluxes; C:Tier 1 EEC=channel morphology/hydraulics, sediment transport; Stressor=altered hydraulic regime and Tier 1 EEC = channel morphology/hydraulics, sediment transport; Inter-tier interaction=sediment transport dynamics; D:Tier 1 EEC=channel morphology/hydraulics, sediment transport; Stressor=altered hydraulic regime and Tier 1 EEC=channel morphology/hydraulics, sediment transport; Inter-tier interaction=sediment transport dynamics; E:Tier 1 EEC=biogeochemistry/thermodynamics; Inter-tier interaction=sediment adsorption of contaminants and nutrients; F: Tier 3 EEC=White

Sturgeon larvae production; Stressors=larval White Sturgeon habitat quantity and quality and Tier 3 EEC=White Sturgeon larvae production, age-0 White Sturgeon recruitment; Inter-tier interaction=mortality and White Sturgeon egg quality and production; Inter-tier interaction=predation of White Sturgeon eggs by native fish and Tier 3 EECs=White Sturgeon larvae production; Inter-tier interaction=predation of White Sturgeon larvae by native fish and Tier 3 EECs=all; Inter-tier interactions=trophic level interactions.

Fig S6. Largemouth bass (*Micropterus salmoides*) data from the Pool 13 of the Upper Mississippi River (A, B) and the La Grange Pool of the Illinois River (C, D). The two river reaches are roughly the same latitude, but the La Grange Pool is more limited in overwintering habitat. Population abundance is presented in panels A and C where each point is an individual fish cumulatively caught with standardized day time electrofishing (Ratcliff et al. 2014) in a specific year. The dashed triangle highlights ‘missing’ >400 mm size classes since 2000 in the La Grange Pool. Population size structure is indexed by proportional stock density (PSD) is presented in panels B and D with the dashed line showing trends in the largest size classes over time. Data and methodology were downloaded from the publicly available databases via the Upper Mississippi River Restoration’s Long Term Resource Monitoring Graphical Fish Browser [115].

Fig S7. The spatial and temporal scales of the management goal, the scientific inferences needed to inform the management goal, and that data collection needs to occur to support the inferences for monitoring information needs identified as requiring additional data in the case study addressing native fish biodiversity and habitat diversity in the Mississippi and Illinois rivers (see Table S4 for additional detail). A:Tier 1 EEC=sediment transport; Stressor=altered hydraulic regime and Tier 1 EEC=biogeochemistry/thermodynamics; Stressor=altered biogeochemical

regime and Tier 1 EEC=biogeochemistry/thermodynamics; Inter-tier interaction=sediment adsorption of contaminants and nutrients; B: Tier 2 EEC=adult native fish overwintering habitat; Stressors=water velocity, water temperature, dissolved oxygen, sediment deposition and Tier 2 EEC=juvenile native fish habitat; Stressors=water depth, water velocity, water temperature, dissolved oxygen, contaminants, sediment deposition and Tier 2 EEC=native fish spawning habitat; Stressors=water depth, water velocity, habitat fragmentation, sediment deposition, water temperature, dissolved oxygen, contaminants and Tier 3 EEC=adult and juvenile native fish recruitment; Inter-tier interaction=mortality and Tier 3 EEC=all; Stressors=invasive species and Tier 3 EEC=all; Inter-tier interaction=trophic level interactions; C:Tier 1 EEC=channel morphology/hydraulics; Inter-tier interaction=channel forming processes; D:Tier 1 EEC=channel morphology/hydraulics, sediment transport; Inter-tier interaction=sediment transport dynamics; E:Tier 3 EEC=adult native fish recruitment; Stressor=adult native fish overwintering habitat quantity and quality and Tier 3 EEC=juvenile native fish recruitment; Stressor=juvenile native fish habitat quantity and quality and Tier 3 EEC=native fish egg quality and production; Stressor=spawning habitat quantity and quality.

Table S1. Summary of information needs identified in the Conceptual Model describing factors affecting the recruitment of the Arkansas River Shiner in the South Canadian River, OK (Fig 4; this publication) by Essential Ecosystem Characteristic (EEC) Tier, EEC, and stressor or within Tier interactions and an assessment of the status of existing information that could be used to address the information needs.

Table S2. Summary of information needs identified in the Conceptual Model describing factors affecting the recruitment of the Humpback Chub in the Colorado River, Arizona (Fig 6; this publication) by Essential Ecosystem Characteristic (EEC) Tier, EEC, and stressor or inter-tier

interactions and an assessment of the status of existing information that could be used to address the information needs.

Table S3. Summary of information needs identified in the Conceptual Model describing factors affecting the recruitment of age-0 White Sturgeon in the Columbia River (Fig 7; this publication), by Essential Ecosystem Characteristic (EEC) Tier, EEC, and stressor or inter-tier interactions and an assessment of the status of existing information that could be used to address the information needs.

Table S4. Summary of information needs identified in the Conceptual Model describing factors affecting the restoration and maintenance of native fish biodiversity and habitat quantity and quality in the Upper Mississippi and Illinois rivers (Fig 8; this publication), by Essential Ecosystem Characteristic (EEC) Tier, EEC, and stressor or inter-tier interactions and an assessment of the status of existing information that could be used to address the information needs.

Figure

Click here to access/download;Figure;Fig 1.tif

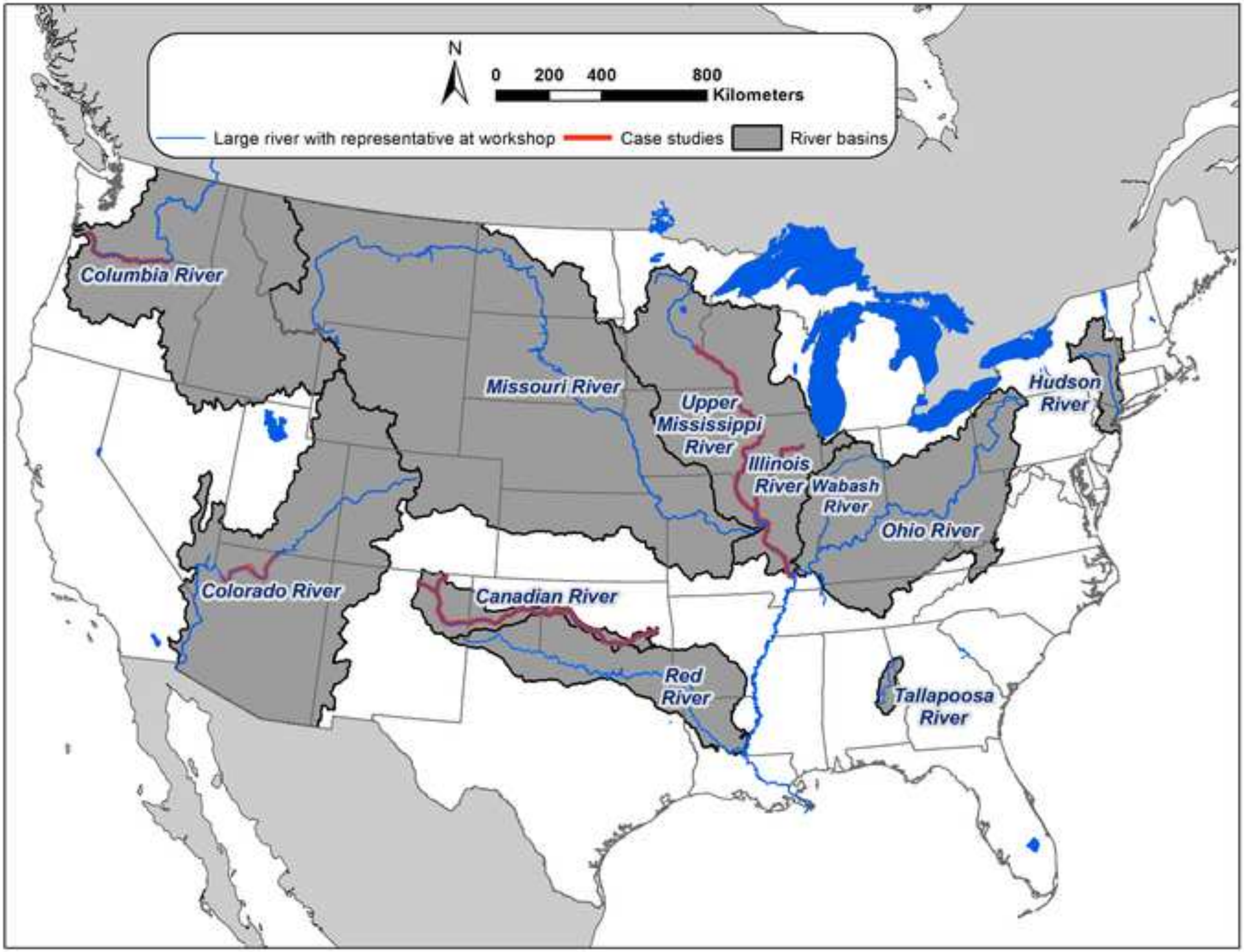

Figure

Click here to access/download;Figure;Fig 2.tif

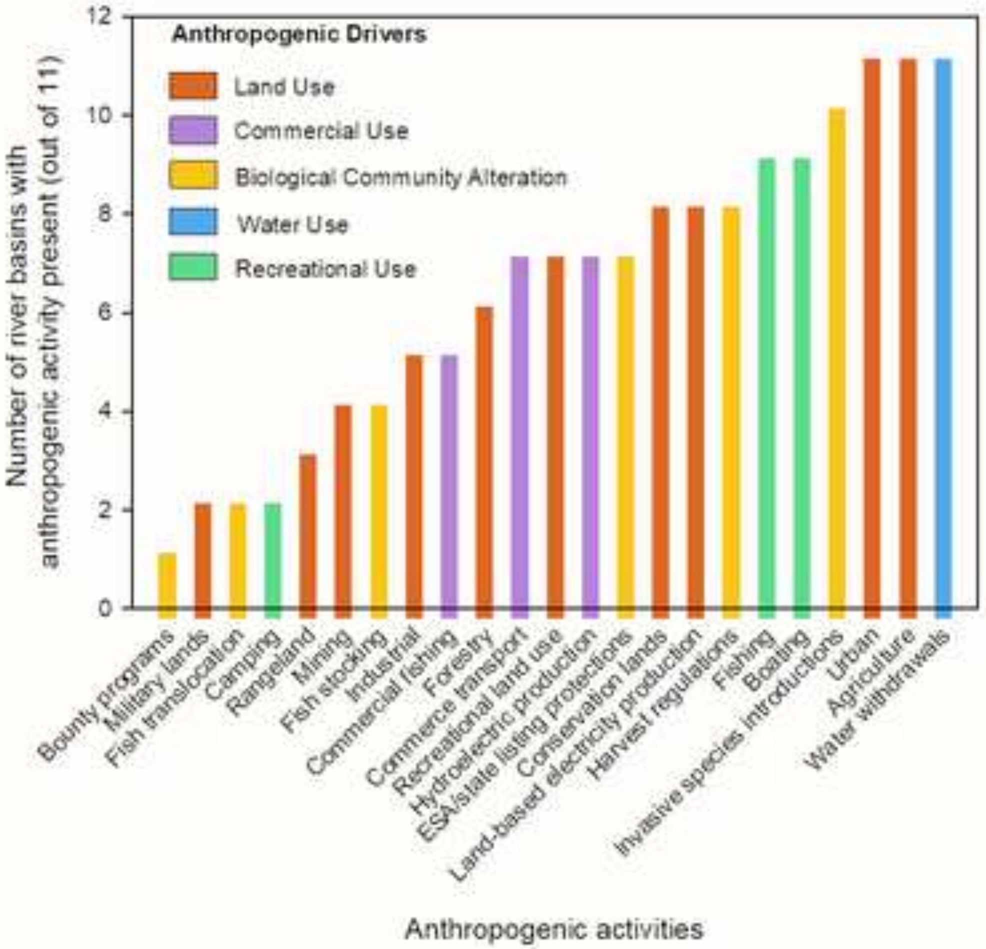

Figure

Click here to access/download;Figure;Fig 3.tif

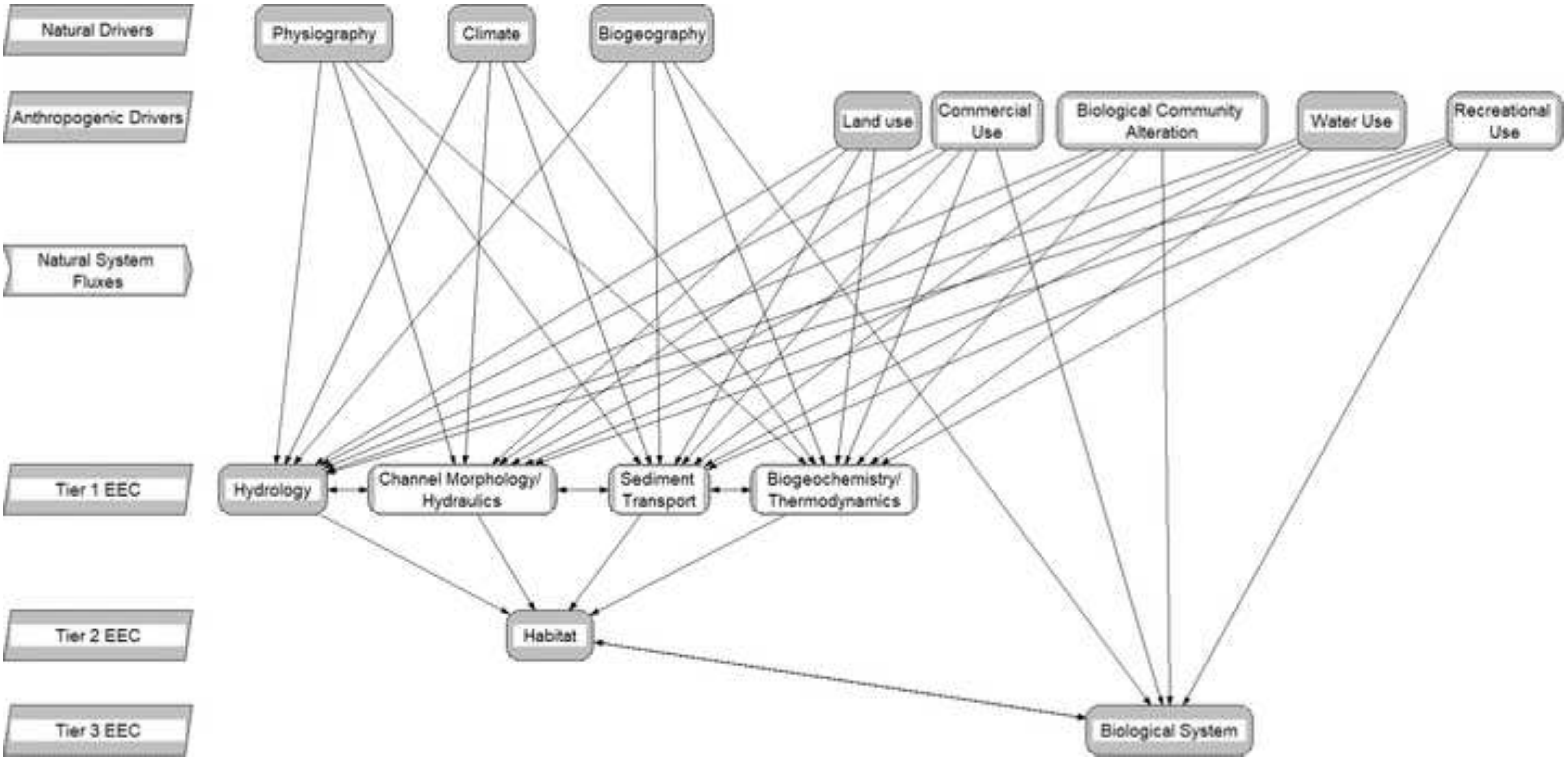

Figure

Click here to access/download;Figure;Fig 4.tif

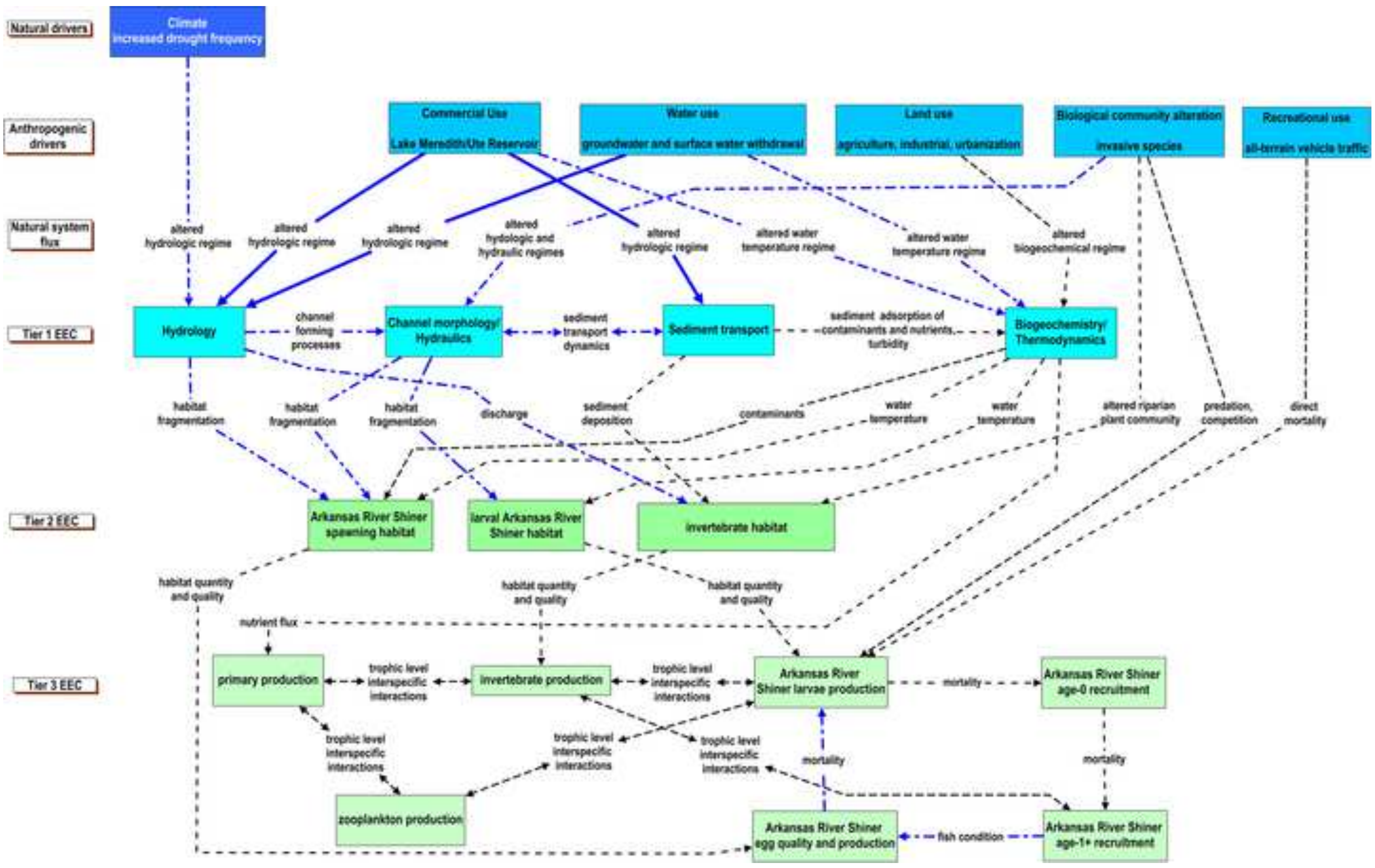

 Click here to access/download;Figure;Fig 5.TIF

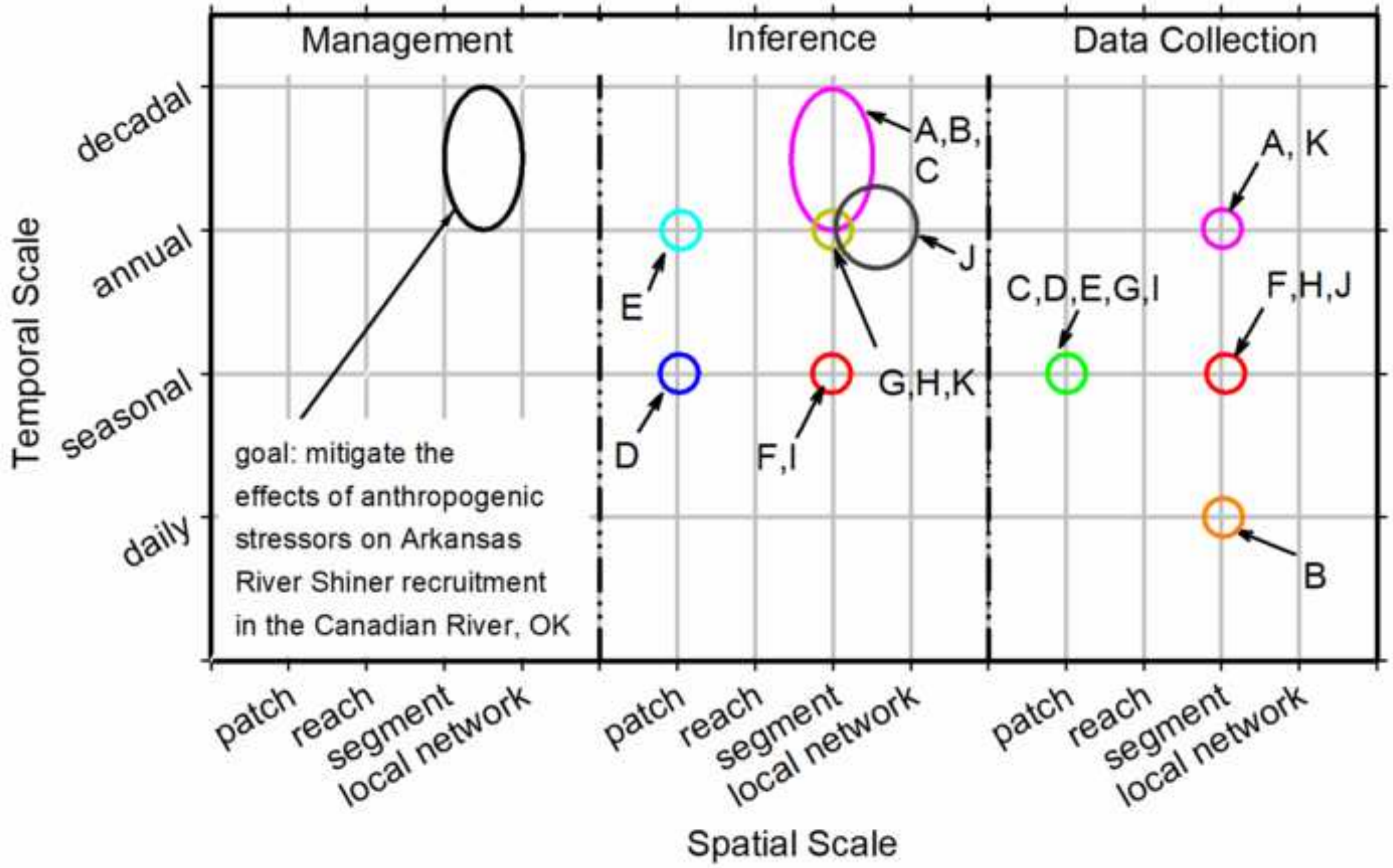

Click here to access/download;Figure;Fig 6.tif

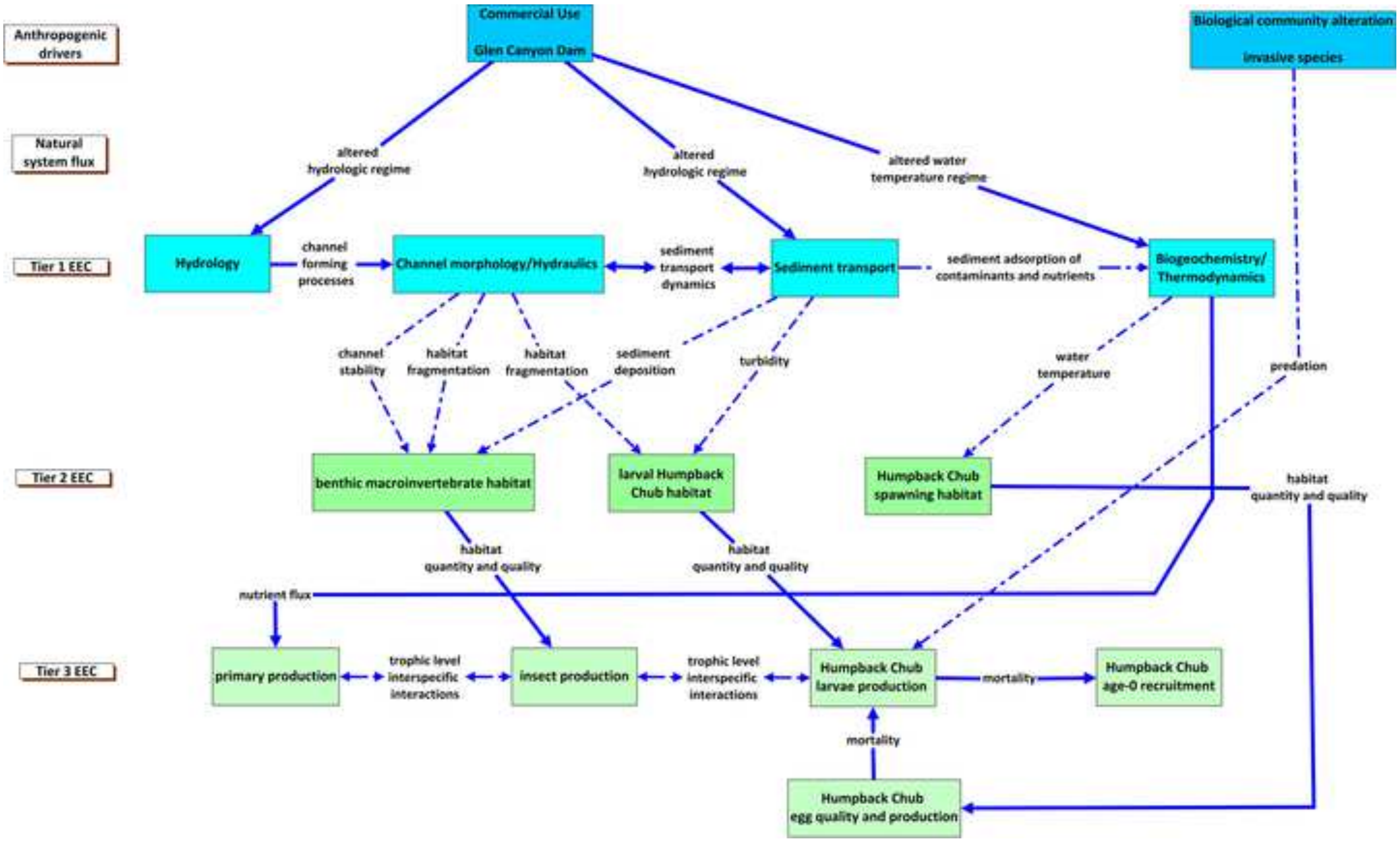

Click here to access/download;Figure;Fig 7.tif

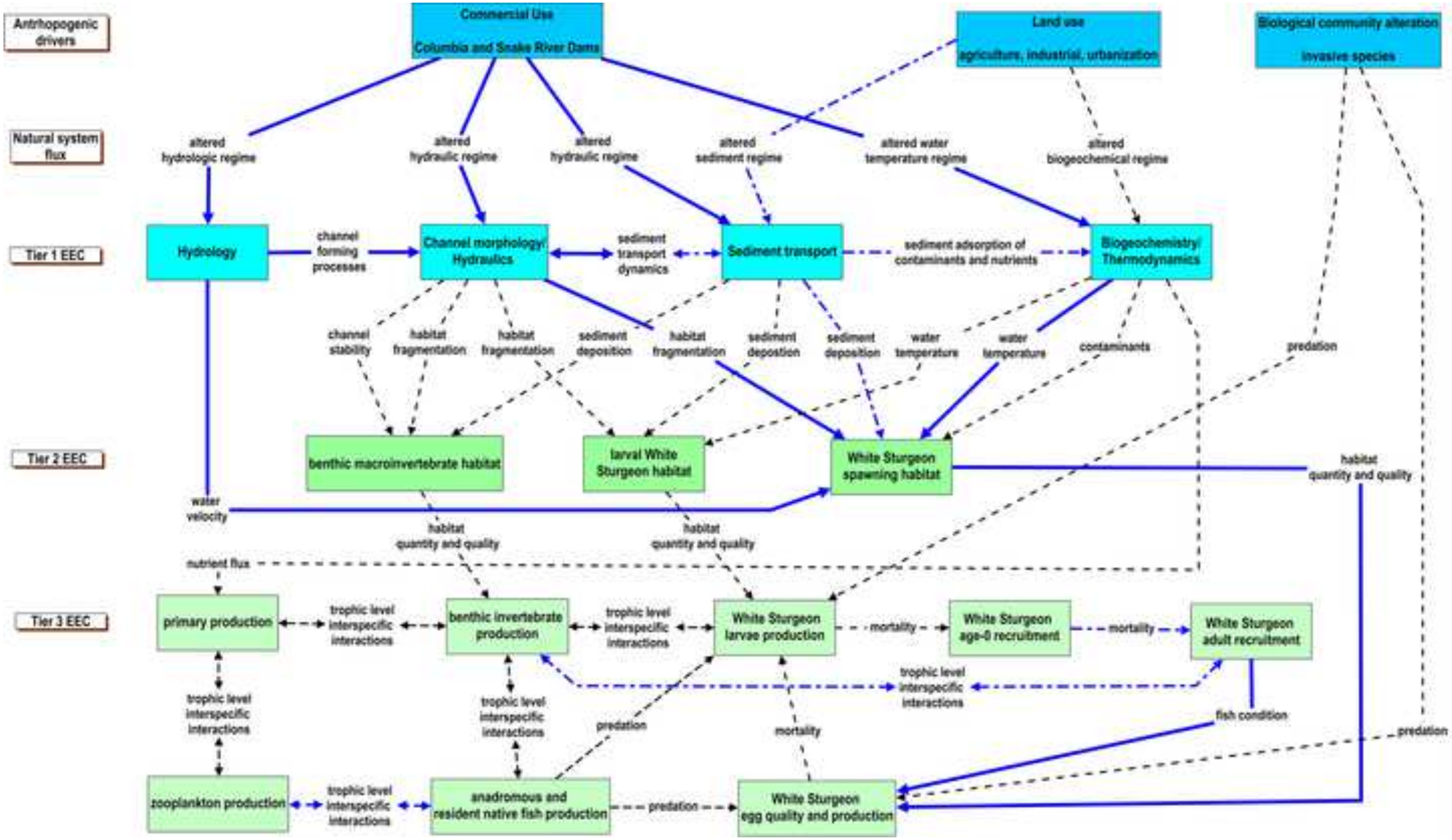

Figure

Click here to access/download;Figure;Fig 8.tif

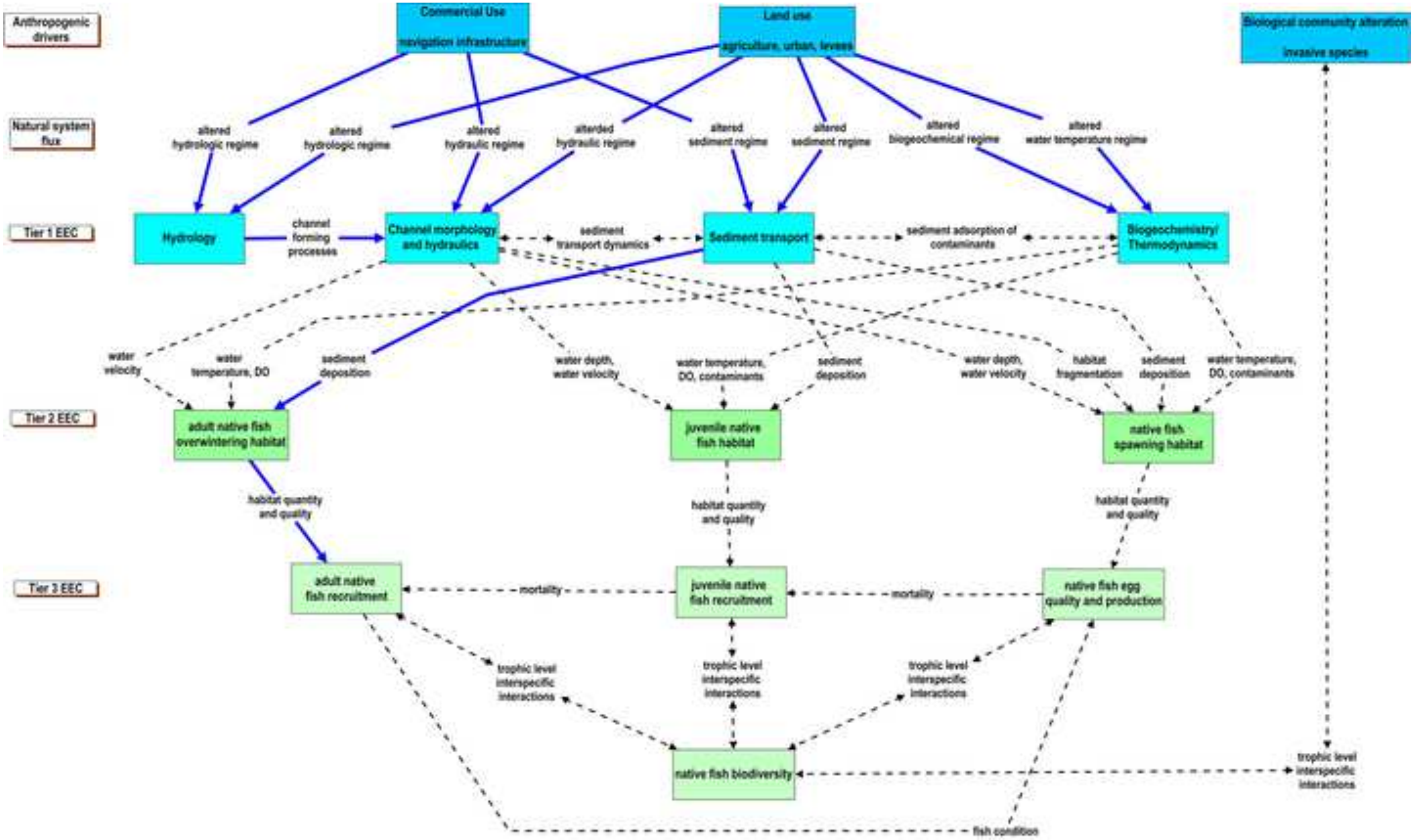

Click here to access/download
Supporting Information
Appendix S1.docx

Click here to access/download
Supporting Information
Fig S1_Counihan_et al. LR CM.docx

Click here to access/download
Supporting Information
Fig S2_Counihan_et al. LR CM.docx

Click here to access/download
Supporting Information
Fig S3_Counihan_et_al. LR CM.docx

Click here to access/download
Supporting Information
Fig S4_Counihan_et al. LR CM.docx

Click here to access/download
Supporting Information
Fig S5_Counihan_et_al. LR CM.docx

Click here to access/download
Supporting Information
Fig S6_Counihan_et al. LR CM.docx

Click here to access/download
Supporting Information
Fig S7_Counihan_et al. LR CM.docx

Click here to access/download
Supporting Information
Table S1_Counihan_et al. LR CM.docx

Click here to access/download
Supporting Information
Table S2_Counihan_et al. LR CM.docx

Click here to access/download
Supporting Information
Table S3_Counihan_et al. LR CM.docx

Click here to access/download
Supporting Information
Table S4_Counihan_et al. LR CM.docx

Title: Identifying monitoring information needs that support the management of fish in large rivers

Timothy D. Counihan, U.S. Geological Survey, Western Fisheries Research Center, Columbia River Research Laboratory, 5501A Cook-Underwood Road, Cook, WA 98605, email: tcounihan@usgs.gov, Phone:509-538-2299, Fax:509-538-2843, ORCID: 0000-0003-4967-6514

Kristen L. Bouska, U.S. Geological Survey, Upper Midwest Environmental Sciences Center, La Crosse, WI 54603, ORCID: 0000-0002-4115-2313

Shannon K. Brewer, U.S. Geological Survey, ~~Oklahoma~~Alabama Cooperative Fish and Wildlife Research Unit, ~~Stillwater, OK 74078~~Auburn, AL 36849, ORCID: 0000-0002-1537-3921

Robert Jacobson, U.S. Geological Survey, Columbia Environmental Research Center, Columbia, MO 65201, ORCID: 0000-0002-8368-2064

Andrew F. Casper, Illinois Natural History Survey, Illinois River Biological Station, Havana, IL, 62644, ORCID: 0000-0003-3450-0209

Colin G. Chapman, Oregon Department of Fish and Wildlife, Ocean Salmon and Columbia River Program, Clackamas, OR 97015, ORCID: 0000-0002-6173-250X

Ian R. Waite, U.S. Geological Survey, Oregon Water Science Center, Portland, OR 97201, ORCID: 0000-0003-1681-6955

Kenneth R. Sheehan, U.S. Geological Survey, Grand Canyon Monitoring and Research Center, Flagstaff, AZ 86001, ORCID: 0000-0003-0362-0793

Mark Pyron, Ball State University, Muncie, IN 47306, ORCID: 0000-0003-0451-7827

Elise R. Irwin, U.S. Geological Survey, Alabama Cooperative Fish and Wildlife Research Unit, Auburn, AL 36849, ORCID: 0000-0002-6866-4976

Karen Riva-Murray, U.S. Geological Survey, Northeast Region, Troy, NY 12180, ORCID: 0000-0001-6683-2238

Alexa J. McKerrow, U.S. Geological Survey, Science Analytics and Synthesis, Core Science Systems~~, David Clark Labs, North Carolina State University~~, Raleigh, NC 27695, ORCID: 0000-0002-8312-2905

Jennifer M. Bayer, U.S. Geological Survey, Northwest-Pacific Island Region, Cook, WA 98605, ORCID: 0000-0001-9564-3110

Formatted: Font color: Auto, Pattern: Clear

Abstract – Management actions intended to benefit fish in large rivers can directly or indirectly affect multiple ecosystem components. Without consideration of the effects of management on non-target ecosystem components, unintended consequences may limit management efficacy. Monitoring can help clarify the effects of management actions, including on non-target ecosystem components, but only if data are collected to characterize key ecosystem processes that could affect the outcome. Scientists from across the U.S. convened to develop a conceptual model that would help identify monitoring information needed to better understand how natural and anthropogenic factors affect large river fishes. We applied the conceptual model to case studies in four large U.S. rivers. The application of the conceptual model indicates the model is flexible and relevant to large rivers in different geographic settings and with different management challenges. By visualizing how natural and anthropogenic drivers directly or indirectly affect cascading ecosystem tiers, our model identified critical information gaps and uncertainties that, if resolved, could inform how to best meet management objectives. Despite large differences in the physical and ecological contexts of the river systems, the case studies also demonstrated substantial commonalities in the data needed to better understand how stressors affect fish in these systems. For example, in most systems information on river discharge and water temperature were needed and available. Conversely, information regarding trophic relationships and the habitat requirements of larval fishes were generally lacking. This result suggests that there may be a common need for a better understanding of certain factors across large-river systems.

Introduction

~~Long-term monitoring has benefited a variety of marine and freshwater ecosystems, including large rivers like the Ohio [1, 2] and Illinois rivers [3]. Large-river systems are complex, making the development of effective monitoring programs especially difficult. Large rivers are dynamic systems with high variability in spatio-temporal physicochemical characteristics and biotic assemblages [4]. The inherent complexity of large rivers makes biotic assemblages logistically difficult to sample [5] and the mechanisms of change difficult to understand. Large rivers represent the culmination of vast stream networks and, thus, integrate and accumulate the effects of multiple stressors at varying spatial scales [6]. The spatial and temporal complexity associated with large rivers has hindered the identification of mechanisms driving declining populations of aquatic species~~ Long-term monitoring has benefited a variety of marine and freshwater ecosystems, including large rivers like the Ohio [1, 2] and Illinois rivers [3]. Large-river systems are complex, making the development of effective monitoring programs especially difficult. Large rivers are dynamic systems with high variability in spatio-temporal physicochemical characteristics and biotic assemblages [4]. The inherent complexity of large rivers makes biotic assemblages logistically difficult to sample [5] and the mechanisms of change difficult to understand. Large rivers represent the culmination of vast stream networks and, thus, integrate and accumulate the effects of multiple stressors at varying spatial scales [6]. The spatial and temporal complexity associated with large rivers has hindered the identification of mechanisms driving declining populations of aquatic species [7-10]~~. To exacerbate the complexity, large rivers commonly have within-channel structural alterations (e.g., dams, river training structures, [11]) and often exhibit legacy effects from historical land uses [12]. To deal~~

with the complexity, some areas of aquatic science recommend monitoring be used to test the linkages developed first through conceptual models (e.g., environmental flows, . To exacerbate the complexity, large rivers commonly have within-channel structural alterations (e.g., dams, river training structures, [11]) and often exhibit legacy effects from historical land uses [12]. To deal with the complexity, some areas of aquatic science recommend monitoring be used to test the linkages developed first through conceptual models (e.g., environmental flows, [13-15]).

Conceptual models are useful tools to help guide the design of monitoring programs [16][16]. The identification of questions relevant to conservation and management efforts requires some foresight and knowledge of the complexities of the system being monitored. For example, it is generally well accepted that the native range of the federally-listed Arkansas River Shiner (*Notropis girardi)* is truncated [17][17], though there is uncertainty surrounding the multiple threats affecting the species [18]. Reducing the uncertainty associated with the decline of the Arkansas River Shiner through the implementation of a hypothesis-driven monitoring program would facilitate confidence in moving forward with a recovery plan. This is where conceptual models are quite useful; they can serve as the foundation to guide hypothesis-driven monitoring programs [14, 16] and identify key ecosystem processes and factors that may directly or indirectly affect management outcomes [19-22].

Understanding factors affecting the status and trends of fishes is of interest to multiple stakeholder groups across multiple jurisdictions. Fishes provide economic benefits to businesses that serve recreational interests, commercial and recreational fishers, tribal members for whom fish are an integral part of their cultural identity [23][23], and to local and state governments who derive revenue from these activities. Fish populations are affected by the integration of physical habitat, water quality, environmental contamination, habitat fragmentation, and overall

ecosystem productivity [24-27]. Consequently, fish are often the focus of management and monitoring programs (e.g., [28]). However, because fish integrate the effects of so many components of the ecosystem, the success of efforts to manage fishes can be affected by unintended consequences of mitigation on factors not directly targeted by the actions. Without consideration of the effects of management on non-target ecosystem components, unintended consequences may limit management efficacy.

Our goal is to demonstrate how a structured, yet flexible, conceptual model (CM) can be used to identify the types of monitoring information needed to understand the range of factors affecting large-river fishes. Our CM includes a hierarchically structured conceptualization of ecosystem characteristics based on CMs originally developed by Harwell, Myers (29) and elaborated by ~~Jacobson and Berkley (30).~~Jacobson and Berkley (30). We chose to incorporate the tiered conceptualization of ecosystem characteristics proposed by ~~Jacobson and Berkley (30)~~Jacobson and Berkley (30) in part because it allows users to define their own biotic or abiotic interests. In this paper, we discuss the structure and development of the CM. We apply the CM to case studies to illustrate the flexibility and applicability of this approach and use it to identify monitoring information needs specific to disparate management goals. More specifically, for each case study, we use the CM to hypothesize how human activities affect fish populations and then identify information needs required to evaluate the hypothesized relationships. We then posit the spatial and temporal scales of the management goal addressed in the conceptual model, inferences needed to inform the management goal, and data collection requirements needed to make the inferences.

Formatted: Space After: Auto, Add space between paragraphs of the same style

Conceptual Model

Overview of Approach

Since 2012, scientists working on large rivers across the United States have participated in a forum intended to improve our understanding of large-river ecosystems. The collaborative forum has worked to identify best practices of long-term monitoring programs [31] and evaluate trends in fish assemblages across rivers [32]. As this group of scientists moved toward linking changes in fish populations and assemblages to human activities, there was a need to develop a process to help identify and prioritize the information needed to assess trends in large river fishes. To that end, a workshop was convened in Hood River, Oregon in May 2017, to jointly adapt, apply, and qualitatively evaluate a conceptual model for developing hypotheses that detail stressors affecting fishes arising from natural and anthropogenic sources ~~[33]~~[33].

Our general approach was to first identify human activities that affect large-river fishes and then hypothesize how the activities related to physical and chemical factors and biological communities. Prior to the workshop, we elicited opinion from experts that comprise a U.S. Geological Survey (USGS) led forum on large river monitoring about important anthropogenic activities that could affect fish populations or communities in the river basins they work in. (Fig 1). We summarized the information from this exercise and grouped the anthropogenic activities into driver categories (Fig 2) and proposed a general form of the CM. We then disseminated the information to the experts prior to the workshop.

Fig 1. Map of rivers and watersheds represented by scientists that convened to develop a conceptual model that ~~would depict~~depicts how natural and anthropogenic drivers interact with

habitats, biological systems, and fish in large rivers. River segments where we conducted case studies that applied the conceptual model to identify monitoring information needs associated with management goals are highlighted in red.

~~Fig 2. The types of anthropogenic activities affecting large-river fishes across 11 river systems as identified from a query to expert participants of a workshop convened to develop a conceptual model that would depict how natural and anthropogenic drivers interact with habitats, biological systems, and fish in large rivers. Meeting participants included research~~Fig 2. The results of a query to scientists from the Canadian, Colorado, Columbia, Hudson, Illinois, Ohio, Missouri, Red, Tallapoosa, Upper Mississippi, and Wabash Rivers, U.S~~.~~ to identify anthropogenic activities that affect large-river fishes in the river systems they represent. Anthropogenic activities were classified into five ~~anthropogenic~~ driver categories.

During the workshop we discussed and refined the CM form (Fig 3). We then had representatives from each river system represented at the workshop choose a management goal to address. Then, through a facilitated discussion led primarily by the representative of the river system being addressed, we 1) elaborated tiered conceptualizations of ecosystem characteristics to reflect the large-river systems and management goals being examined, 2) used knowledge of the fish species' life history and population bottlenecks to relate biological ecosystem characteristics to habitat requirements, 3) hypothesized pathways describing how anthropogenic and natural drivers affect large-river fish populations either indirectly (~~i.~~e.g., effects on flow regime, habitat, trophic resources, etc.) or directly (e.g., competition with invasive species), and 4) hypothesized interactions within ecosystem characteristic tiers that could affect the

Formatted: List Paragraph

Formatted: Font: Calibri, 11 pt

Formatted: Add space between paragraphs of the same style

management goal. Based on this exercise, we chose four case studies to refine for use in this manuscript (Fig 1).

Fig 3. Tiered hierarchical conceptualization of how anthropogenic and natural drivers relate to physical and biological components of large-river ecosystems. Essential ecosystem characteristics (EECs) are groupings of ecosystem components. Tier 1 EECs represent physical and chemical effects; fundamental measures of process that are directly affected by anthropogenic and natural drivers. Tier 2 EECs represent a broad habitat category that is intended to encompass the physical, chemical, and biological components of the riverine habitats that influence reproduction, growth, and survival of biotic communities. The Tier 3 EEC represent components of the biological systems that respond to changes in the hierarchical components of the conceptual model.

After the workshop, we held a series of conference calls with workshop participants with expertise in the selected case studies to refine all aspects of the CMs and associated information. During the calls, we started with the CM from the workshop and discussed and clarified CM components, pathways, and inter-tier interactions. We then characterized whether, based on the expert knowledge of workshop participants, there was a strong, moderate, or weak understanding of the pathways and interactions. ~~We then developed a~~A list of the information needed to understand the relationships described in the CM was developed. We then had the case study leader classify whether the data required to understand the information needs were available, insufficient, or not available. For information needs that were classified as insufficient or not

available, we ~~then~~ characterized the spatial and temporal scales at which data should be collected to make inferences that support the evaluation of management goals.

We then encouraged representatives from each basin to share the CM and the case study narrative with other experts familiar with the river system and management goal. This outreach took several forms including sharing the CM with working groups tasked with implementing the management goal, discussions with peers familiar with the management goal, and presenting the CM at regional conferences. The intent was to garner opinions from outside the workshop participants. If needed, the CMs incorporated the feedback received.

Hierarchical structure of Conceptual Model

Our CM is a hierarchical conceptualization of how anthropogenic and natural drivers relate to multiple tiers representing the physical and biological components of large-river ecosystems (Fig 3). Natural drivers included in the CM were physiographic, climatic, and biogeographic factors that control fluxes of water, mass, energy, and genetic information in a watershed ~~[30]~~[30]. The physiographic factors, such as lithology, soils, and watershed topography, exert control on water, sediment, and geochemical fluxes (e.g., nutrients) into the river corridor. Physiography is generally static over time frames of decades to centuries. Climate controls fluxes of atmospheric energy and moisture into the watershed. Unlike physiography, climate is more likely ~~variable~~to vary over relatively shorter temporal scales. Biogeography describes the native organism assemblage in the watershed (e.g., ~~[34]~~[34]) and the natural flux of genetic information due to immigrations, emigrations, mutations, and extinctions. Changes arising from the biogeography driver includes altered spatial distribution of organisms within the

watershed, which, in turn, may alter the effects of natural system regimes on the river corridor. For example, natural variation of the type and distribution of vegetation can affect the time series and magnitude of runoff events [30][30].

We created five categories of anthropogenic drivers to characterize a range of human activities that affect large rivers: land use, commercial use, biological community manipulationalteration, water use, and recreation (Fig 2). The land-use category is intended to reflect different ways humans use landscape resources that affect large rivers. We defined commercial use as the use of river resources for marketable enterprises that did not involve water removal or transfer. We included biological community alteration to represent the intentional or non-intentional human alteration or manipulation of the river's biological community (e.g., introductions of non-native fish). Recreational use was defined as the use of river resources for leisure activities (e.g., fishing, boating). We considered water use a direct commercial or non-commercial use of river water that involved the removal or transfer of water.

Our CM includes hierarchically structured essential ecosystem characteristics (EEC), originally developed by Harwell, Myers (29) and described in detail by Jacobson and Berkley (30).Jacobson and Berkley (30). Briefly, EECs are characteristics that can be classified into similar groups based on the way they link to biological endpoints [30][30]. Tier 1 EECs are measurable characteristics that describe processes that can significantly alter the morphological or chemical characteristics within a river channel. The Tier 1 categories we considered were 1) Hydrologyhydrology, 2) Channel Morphology/Hydraulicschannel morphology/hydraulics, 3) Sedimentsediment transport, and 4) Biogeochemistry/Thermodynamicsbiogeochemistry/thermodynamics. Tier 2 EECs are broadly described as physicochemical or biological components of "habitat" that are hypothesized to

affect (e.g., growth, survival, reproduction, ~~[35]~~[35]) fish populations or assemblages. Lastly, Tier 3 EECs represent components of the hypothesized biological system that are affected by the cascading (e.g., degradation of egg quality caused by increases in sediment deposition) or direct (e.g., predation by invasive species) effects of anthropogenic and natural drivers. Tier 2 characteristics are particularly important because these are the factors that can be examined at scales most often sampled by fisheries managers ~~(e.g., Smallmouth Bass *Micropterus dolomieu*, [36]).~~[36]. The specific components that comprise Tier 2 and 3 EECs are flexible and can be adapted and elaborated depending on the river system and specific management goal being addressed.

We retained aspects of the approach taken by ~~Jacobson and Berkley (30)~~Jacobson and Berkley (30) with respect to how our model represents interactions between drivers and EECs, but with key differences. Since we were interested in representing how human activities affect large river ecosystems, our approach acknowledges that anthropogenic and natural drivers interact and alter the expected characteristics of Tier 1 EECs. ~~Similar to Jacobson and Berkley (30)~~Similar to Jacobson and Berkley (30), our model depicts a stress associated with a natural or anthropogenic driver to Tier 1 EECs as fluxes in natural system regimes that alter the frequency, magnitude, duration, timing, or rate of change in natural systems or by the imposition of a hard-structural constraint on channel form. The natural system regimes considered in our CM were hydraulic, hydrologic, sediment, temperature, light, and biogeochemistry. Graphically, the natural system fluxes were represented by arrows connecting anthropogenic and natural drivers to Tier 1 EECs. Similarly, hypothesized pathways between EECs, that depict the expression of the cascading effects of anthropogenic and natural drivers, and interaction within EECs were depicted as arrows. ~~All~~For example, fragmentation of river systems resulting from altered

hydrologic and/or hydraulic regimes caused by dams, weirs, levees, and other factors are frequently cited sources of stress to large-river fishes [37]. Fragmentation can prevent fish from migrating and/or dispersing through their natural reproductive ranges and from accessing critical habitats [38]. To depict a scenario where the presence of a dam is altering hydrologic and/or hydraulic regimes resulting in habitat fragmentation, the CM would show an arrow from an anthropogenic stress (i.e., dam as a commercial activity) to a Tier 1 EEC (e.g., channel morphology/hydraulics) that would depict a natural system flux (e.g., altered hydrologic and/or hydraulic regime) that would then manifest as a stress caused by habitat fragmentation depicted by an arrow between the Tier 1 EEC and a Tier 2 EEC (e.g. habitat) that would then manifest as an effect on a Tier 3 component, shown by an arrow between Tier 2 and Tier 3. All stress pathways and interactions were classified with respect to the strength of understanding of the relationships based on expert opinion. Arrows with solid blue lines depict a strong understanding of the relationship, dotted-dashed blue lines represent a moderate understanding of the relationship, and with a black dashed line represent a weak understanding of the relationship.

Spatial and temporal context

The successful characterization of how human activities influence large-river fishes is dependent upon integrated concepts of scale. Fish distributions in rivers can vary spatially within river basins in relation to naturally occurring and human induced landscape characteristics [32]. Fish distributions can also vary seasonally, annually, and over longer times in response to changing environmental conditions [~~37~~39]. Consequently, the spatial and temporal scope of fish management goals often varies within and between large-river systems and agencies. For data

collected by monitoring programs to have the highest relevance, the spatial and temporal scales appropriate for scientific investigation and management must also be time and geographic-context specific. For example, the management of White Sturgeon (*Acipenser transmontanus*) in the Columbia River varies by reservoir or river segment and season [~~38, 39~~40, 41]. The effects of hydropower development on White Sturgeon vary spatially and temporally as well, so the spatial and temporal context of the data needed to understand the effects needs to be considered. For instance, hydropower peaking operations, that can vary by dam and season, affect river discharge in a river reach on a diel and even hourly basis [~~40~~42], whereas water storage and other management actions can affect seasonal discharges over a broader geographic scale ~~[38].~~[40]. Understanding the spatial and temporal context needed to inform management will help ensure relevant information is collected.

We considered spatial and temporal resolution in our CMs. We defined spatial extent as : ~~Local Network~~local network – synonymous with Hydrologic Unit Code (HUC) 2 basins [~~41, 42~~43, 44]; ~~Segment~~segment – the portion of a river between two major tributary confluences ~~[43]~~[45] or other hydrogeomorphic features ~~[44]; Reach – the length of river occurring between breaks in channel slope caused by man-made dams or other hydrogeomorphic features [43]; Patch~~[46]; reach - the length of river occurring between breaks in channel slope caused by man-made dams or other hydrogeomorphic features [45]; patch – an area used by an organism (e.g., for reproduction or resource attainment) that can vary both spatially and temporally depending on the species of interest [~~45, 46~~47, 48]. For our purposes, the spatial scales considered are nested such that segments occur within local networks, reaches occur within segments, and patches occur at the sub-reach scale. Temporal units considered were daily, seasonal, annual, and

decadal. The temporal units were used to denote both the scale of inferences needed to support the management goal and the scale at which data should be collected to inform the inferences.

Case Studies

We applied the CM to four case studies. For each, we followed the pathways of stress from Tier 1 EECs to the biological endpoint associated with the management goal to identify information needs. We then characterized the spatial and temporal scales of the management goal, the scientific inferences needed to inform the management goal, and that data collection needs to occur to support the inferences for monitoring information needs identified as requiring additional data for each of the case studies. To summarize similarities across case studies, we generalized the stressors and inter- tier interactions identified in the case studies and then summarize the similarities by EEC tier. More context for the river systems characterized in the case studies can be found in Appendix S1.

South Canadian River

~~The South Canadian River is the mainstem river of the Canadian River basin (south-central U.S., Fig 1). The basin occupies a significant west-east climate gradient with precipitation ranging from 40 to 145 cm/yr [47]. Land use in the basin is primarily cropland or pasture that transitions to the urban area near Oklahoma City. Three major reservoirs on the South Canadian River have significantly altered the river flow patterns, particularly the frequency and magnitude of high-flow events, and the number of zero flow days (Fig S1). Multiyear to decadal droughts are not uncommon in the region [48], and groundwater pumping,~~

particularly in the upper basin, has severed groundwater connections in many areas transforming the fish assemblage [49]. Further, groundwater pumping in the alluvial aquifer has decreased stream baseflows within this region [50, 51]. The rivers of the Great Plains are characterized by extreme physicochemical conditions (i.e., water temperatures 4 - 40°C; salinities greater than ocean water) and extensive flooding and extended periods of drought, and native fishes are well adapted to those extremes. Pelagic broadcast spawning minnows (i.e., pelagophils) belong to a reproductive guild of diminutive fishes that are emblematic of these stream systems. Of the approximately 20 species in this reproductive guild, 13 are of conservation concern; the status of the remaining seven species is poorly understood [18, 52]. Native populations of the federally-threatened Arkansas River Shiner are believed to be restricted to two fragmented portions of the South Canadian River [5349].

The Arkansas River Shiner is hypothesized to be affected by several anthropogenic activities that primarily affect water quality and quantity (Fig 4). Three reservoirs on the South Canadian River have altered discharge patterns (Fig S1), and fragmented river habitats. Two known native populations of Arkansas River Shiner occupy the two remaining river segments of sufficient length and complexity to allow eggs to drift the time required to successfully complete their early life history. Small impoundments for agriculture use, road crossings, groundwater pumping and other local water extractions (e.g., oil and gas) threaten to further fragment existing habitat. Fragmentation could also be problematic for upstream fish migrations; there is some evidence that Arkansas River Shiners migrate upstream to spawn to achieve adequate drift distances for their offspring [54].[50]. It has also been speculated that this species might benefit from access to floodplain habitats [55][51], but we are unaware of efforts to examine that hypothesis. Changes in the flow patterns may also relate to the expansion of salt cedar *Tamarix*

Formatted: Add space between paragraphs of the same style

spp. and other non-native riparian species that constrain the channel and inhibit channel habitat complexity [~~56, 57~~52, 53]. Changes to the riparian corridor can also alter the availability of drifting invertebrates~~ available~~ for Arkansas River Shiner feeding (i.e., Coleoptera, Hymenoptera; ~~[58]).~~[54]). Channel complexity acts to slow the transport of eggs [~~53~~49] and may prevent eggs from being washed into downstream reservoirs where survival is hypothesized to be extremely low. Climate change is expected to increase the intensity and frequency of drought events within this region [~~59, 60~~55, 56], which may exacerbate habitat fragmentation, promote ~~ATV~~all-terrain vehicle traffic within the river channel causing direct mortality on stranded fish (Gene Wilde, Texas Tech University, Personal Comm.), and concentrate contaminants and salinity [~~61~~57]. The tolerances of Arkansas River Shiner to salinity concentrations and many other contaminants are unknown (see Table ~~3~~S1; [18]). Lastly, introductions of non-native fishes ~~via bait buckets ~~have occurred within the basin. The primary concern ~~has related to~~is the presence of Red River Shiner (*Notropis bairdi*) because it is suspected to reproduce in a similar manner and be a possible competitor to the Arkansas River Shiner [~~61~~57].

Fig 4. Conceptual model describing the relationship of natural and anthropogenic drivers to essential ecosystem characteristics (EECs) affecting the recruitment of the Arkansas River Shiner in the South Canadian River in New Mexico, Texas, and Oklahoma. Essential ecosystem characteristics are groupings of ecosystem components. Tier 1 EECs represent physical and chemical effects; fundamental measures of process that are directly affected by anthropogenic and natural drivers. Tier 2 EECs represent a broad habitat category that is intended to encompass the physical, chemical, and biological components of the riverine habitats that influence reproduction, growth, and survival of biotic communities. The Tier 3 EECs represent

components of the biological systems that respond to changes in the hierarchical components of the conceptual model. The strength of our understanding of how natural and anthropogenic drivers interact with habitats, biological systems, and fish in large rivers is represented by the different types of lines in the figure. Solid blue lines depict a strong understanding of the relationship, the dotted-dashed blue line represents a moderate understanding of the relationship, and the black dashed line represents a weak understanding of the relationship. The different types of lines also represent the strength of our understanding of within EEC-tier relationships.

The results of the CM exercise that characterized factors affecting the Arkansas River Shiner in the South Canadian River suggested the critical life-history bottlenecks for the Arkansas River Shiner are successful spawning and recruitment to the first year. Impediments that limit our understanding of factors that lead to successful spawning and recruitment included the effects of channel morphology and hydraulics on the quality and quantity of larval rearing habitat, and subsequent effects on larval production (Table S1). Water use and other drivers occurring at relatively coarse spatial and temporal scales are the hypothesized drivers related to degradation of reproductive habitat for the Arkansas River Shiner (Fig 5). A temporal lag in responses at finer scales (i.e., improved habitat) would be anticipated with management actions at these coarser spatial scales (~~i.~~e.g., water releases from dams); though, providing connectivity via minimal water releases would occur relatively quickly. Although there are gages on the South Canadian River, the spacing of the gages is not sufficient to have a full understanding of flow patterns between the gages given the semi-arid nature of the basin and potential for reaches to be affected by water withdrawals such as groundwater pumping. Our understanding of the species life history is well established; however, the effects of human pressures on the species

and associated habitat has not been well studied (i.e., production, survival). ~~As indicated in Fig 4, the only stress pathway that was characterized as being well understood was from land-use stressors to Tier 1 processes. As those~~As stressors propagate through Tier 1 to Tier 2 and Tier 3, the level of uncertainty ~~increases~~increased such that it is not possible to define a preferred hypothesis for Arkansas River Shiner recruitment failure. The status of ~~much existing~~ information ~~was~~needed to understand the hypothesized stress pathways and interactions was mostly characterized as insufficient or not available (Table S1).

Fig 5. The spatial and temporal scales of the management goal, the scientific inferences needed to inform the management goal, and that data collection needs to occur to support the inferences for monitoring information needs identified as requiring additional data in the case study addressing the recruitment of the Arkansas River Shiner in the Canadian River, Oklahoma (see Table S1 for additional detail). A:Tier 1 EEC=~~Channel~~channel morphology/~~Hydraulics~~hydraulics; Stressor=altered hydraulic regime; B:Tier 1 EEC=~~Biogeochemistry/Thermodynamics~~biogeochemistry/thermodynamics; Stressor=altered water temperature regime; C:Tier 1 EEC=~~Biogeochemistry/Thermodynamics~~biogeochemistry/thermodynamics; Stressor=altered biogeochemical regime; D:Tier 2 EEC=Arkansas River Shiner spawning habitat; Stressors=contaminants, water temperature, habitat fragmentation; E:Tier 2 EEC=larval Arkansas River Shiner habitat, Stressors=water temperature, habitat fragmentation and Tier 2 EEC=invertebrate habitat, Stressors=altered riparian plant community, discharge, sediment deposition; F:Tier 3 EEC=primary production, Stressor = nutrient flux; G:Tier 3 EEC=invertebrate production; Stressor=invertebrate habitat quantity and quality and Tier 3

EEC=Arkansas River Shiner larvae production, Stressor=predation by invasive species; H:Tier 1 EEC= ~~Biogeochemistry/Thermodynamics~~biogeochemistry/thermodynamics; Inter-tier interaction= Sediment adsorption of contaminants and nutrients and Tier 3 EEC=Arkansas River Shiner larvae production, Stressors=Arkansas River Shiner larvae habitat quantity and quality and Tier 3 EEC=Arkansas River Shiner age-0 recruitment; Inter-tier interaction=Arkansas River Shiner larvae mortality and Tier 3 EEC=Arkansas River Shiner age-1+ recruitment, Inter-tier interaction= Arkansas River Shiner age-0 mortality and Tier 3 EEC=all, Inter-tier interaction=trophic level interactions; I:Arkansas River Shiner larvae production; Stressor= direct mortality from recreational use (i.e., ~~ATV~~all-terrain vehicle and in-river traffic); J:Tier 3 EEC=Arkansas River Shiner egg quality and production; Stressor=Arkansas River Shiner spawning habitat quantity and quality; K:Tier 3 EEC=~~Larval~~ Arkansas River Shiner larvae production; Inter-tier interaction=Arkansas River Shiner egg mortality.

Colorado River

~~The Colorado River flows 2,330 km from its origin in Colorado to its confluence with the Gulf of California in Mexico draining parts of seven U.S. and two Mexican states; our assessment focused on the portion of the Colorado River that flows through the Grand Canyon in Arizona (Fig 1). Water storage associated with the allocation of water in the Colorado River for human consumption and hydroelectric power generation has altered the hydrologic regime (Fig S2) in the Colorado River between Glen Canyon Dam and Lake Mead. Most fish species native to the Colorado River have declined in abundance and distribution while numerous non-native species have become established [62]. The Humpback Chub (*Gila cypha*), a fish native to the~~

Colorado River, was listed as endangered byThe Humpback Chub (*Gila cypha*), a fish native to the Colorado River, was listed as endangered by the U.S. Fish and Wildlife Service in 1967 and given full protection under the Endangered Species Act of 1973 (ESA). To mitigate the effects of anthropogenic changes to the river on Humpback Chub, an understanding of the mechanisms by which Glen Canyon Dam and non-native species affect Humpback Chub is needed.

A critical life-history bottleneck for Humpback Chub is recruitment into the first year class. (Fig 6). Temperature, light, and seasonal high river discharge from snowmelt are thought to cue spawning behavior [63].[58]. Hydropower development has dampened the range of river discharges of the Lower Colorado River within Grand Canyon. Historically, river discharge varied between 15 and 3400 m^3/s, however discharge was greater in large flood events; current dam operations limit flows to a range of 140 to 1000 m^3/s (Fig S2). Resulting changes in turbidity and water temperature create risks to endangered Humpback Chub, and other endemic fish. For example, the quantity and quality of habitat is reduced through changes in turbidity, biogeochemistry, and the temperature regime. Hypolimnetic water releases from Lake Powell maintain cold temperatures in the Colorado River downstream from Glen Canyon Dam; currently, spawning is limited to a single tributary, the Little Colorado River. As embryos survive into the larval stage, nursery habitats to support growth and foraging are essential [64-6659-61]. A secondary risk to juvenile survival post-larval stage is piscivory frompredation by non-native species including Rainbow Trout (*Oncorhynchus mykiss*) and Channel Catfish (*Ictalurus punctatus*) [67].[62]. Temperatures for well over 100 km downstream of Lake Powell are excellent for non-native, cold water species, including a closely managed world-class Rainbow Trout fishery at Lees Ferry. Rainbow and Brown Trout (*Salma trutta*) are currently managed as an invasive species downstream of the confluence of the Colorado and Little

Formatted: Add space between paragraphs of the same style

Colorado River, approximately 97 km downstream of Glen Canyon Dam, to mitigate predation upon native fishes including the endangered Humpback Chub.

~~The CM exercise documented a generally high understanding of the relationships of anthropogenic drivers to Tier 1 EECs, cascading to multiple, less-understood hypotheses about how these factors would combine to affect habitats at Tier 2 (Fig 6). High confidence in the linkages from Tier 2 invertebrate habitat to Tier 3 insect population is followed by a lesser understanding of how insect populations are linked to larval production. The uncertainty of the relations between food resources stands in contrast to high certainty that was ascribed to the linkages from larval chub habitat to larval chub production, and from spawning habitat to larval chub production and thence to recruitment. Since 1997, the Glen Canyon Dam Adaptive Management Program has supported extensive monitoring and research across the spatial and temporal landscape of the Colorado River. As a result, the information needed to characterize some of the stressors is readily available (Table S2). However, the status of some existing information was characterized as insufficient or not available. For the information needs characterized as being insufficient or not available, we identified the spatial and temporal scales at which data collection would facilitate the inferences needed to inform the management goal (Fig 7). Understanding how these knowledge gaps affect Humpback Chub recruitment could identify strategies that will help achieve the management goal of improving Humpback Chub recruitment in the Colorado River.~~

Fig 6. Conceptual model describing the relationship of anthropogenic drivers to essential ecosystem characteristics (EECs) affecting the recruitment of Humpback Chub in the Colorado

River between Glen Canyon Dam and Lake Mead, Arizona. Essential ecosystem characteristics (EECs) are groupings of ecosystem components. Tier 1 EECs represent physical and chemical effects; fundamental measures of process that are directly affected by anthropogenic and natural drivers. Tier 2 EECs represent a broad habitat category that is intended to encompass the physical, chemical, and biological components of the riverine habitats that influence reproduction, growth, and survival of biotic communities. The Tier 3 EECs represent components of the biological systems that respond to changes in the hierarchical components of the conceptual model. The strength of our understanding of how natural and anthropogenic drivers interact with habitats, biological systems, and fish in large rivers is represented by the different types of lines in the figure. Solid blue lines depict a strong understanding of the relationship, the dotted-dashed blue line represents a moderate understanding of the relationship, and the black dashed line represents a weak understanding of the relationship. The different types of lines also represent the strength of our understanding of within EEC-tier relationships.

~~Fig 7.~~ The CM exercise documented a high understanding of the relationships of anthropogenic drivers to Tier 1 EECs, cascading to multiple, less-understood hypotheses about how these factors would combine to affect habitats at Tier 2 (Fig 6). High confidence in the linkages from Tier 2 invertebrate habitat to Tier 3 insect production is followed by a lesser understanding of how insect production is linked to larval production. The uncertainty of the relations between food resources stands in contrast to high certainty that was ascribed to the linkages from larval chub habitat to larval chub production, and from spawning habitat to larval chub production and then to recruitment. Since 1997, the Glen Canyon Dam Adaptive Management Program has supported extensive monitoring and research across the spatial and

temporal landscape of the Colorado River. As a result, the information needed to characterize some of the stressors is readily available (Table S2). However, the status of some existing information was characterized as insufficient or not available. For the information needs characterized as being insufficient or not available, we identified the spatial and temporal scales at which data collection would facilitate the inferences needed to inform the management goal (Fig S3). Understanding how these knowledge gaps affect Humpback Chub recruitment could identify strategies that will help achieve the management goal of improving Humpback Chub recruitment in the Colorado River.

~~The spatial and temporal scales of the management goal, the scientific inferences needed to inform the management goal, and that data collection needs to occur to support the inferences for monitoring information needs identified as requiring additional data in the case study addressing Humpback Chub recruitment in the Colorado River between Glen Canyon Dam and Lake Mead, Arizona (see Table S2 for additional detail). A:Tier 2 EEC= larval Humpback Chub habitat; Stressors=habitat fragmentation, turbidity and Tier 2 EEC= Humpback Chub spawning habitat; Stressor= water temperature and Tier 3 EEC=insect production; Stressor=benthic macroinvertebrate habitat quantity and quality and Tier 3 EECs=all; Inter-tier interaction=trophic level interactions; B:Tier 3 EEC=larval Humpback Chub production; Stressor= larval Humpback chub habitat quantity and quality; C: Tier 3 EEC=Humpback Chub egg quality and production; Stressors= Humpback Chub spawning habitat quantity and quality; D:Larval Humpback Chub production; Inter-tier interaction=mortality of Humpback Chub eggs; E:Tier 3 EEC= larval Humpback Chub production; Stressor=predation by invasive species and Tier 3 EEC=Primary production; Stressor=nutrient flux; F:Tier 1 EEC= Biogeochemistry/thermodynamics; Inter-tier~~

interaction=sediment adsorption of contaminants and nutrients; G:Tier 3 EEC=Age-0 Humpback Chub recruitment; Inter-tier interaction=mortality of larval Humpback Chub

Columbia River

White Sturgeon is the largest freshwater fish in North America [63]. Like other sturgeon species, anthropogenic stressors have negatively affected White Sturgeon productivity. Our knowledge of factors affecting White Sturgeon productivity remain poorly understood [40]. Therefore, we used the CM to identify knowledge gaps associated with the hypotheses that dam construction and operation, land-use practices, and invasive species, in some combination, affect the recruitment of age-0 White Sturgeon (Fig 7). Within the basin, development of hydroelectric and water-storage dams have changed the magnitude and seasonality of the natural river discharge (Fig S4) and thermal regimes [64], reduced the quantity and quality of spawning habitats [65, 66], and disrupted historical migration patterns [67]. Prior to hydropower development, White Sturgeon experienced a hydrograph that peaked during June-July due to snowmelt [64]. However, from 1949 to 1993 the average discharge in June decreased from 14,000 m^3/s to 6,000 m^3/s and the maximum water temperature has increased by 1.8°C [64]. White Sturgeon likely used the natural hydrograph and thermal regime as cues to seek out optimal spawning habitats and initiate spawning [65, 68].

Fig 7. Conceptual model describing the relationship of anthropogenic drivers to essential ecosystem characteristics (EECs) affecting the recruitment of White Sturgeon in the Columbia River, U.S. Essential ecosystem characteristics (EECs) are groupings of ecosystem components. Tier 1 EECs represent physical and chemical effects; fundamental measures of process that are

Formatted: Add space between paragraphs of the same style

directly affected by anthropogenic and natural drivers. Tier 2 EECs represent a broad habitat category that is intended to encompass the physical, chemical, and biological components of the riverine habitats that influence reproduction, growth, and survival of biotic communities. The Tier 3 EECs represent components of the biological systems that respond to changes in the hierarchical components of the conceptual model. The strength of our understanding of how natural and anthropogenic drivers interact with habitats, biological systems, and fish in large rivers is represented by the different types of lines in the figure. Solid blue lines depict a strong understanding of the relationship, the dotted-dashed blue line represents a moderate understanding of the relationship, and the black dashed line represents a weak understanding of the relationship. The different types of lines also represent the strength of our understanding of within EEC-tier relationships.

Factors other than river discharge and water temperature may also be affecting age-0 White Sturgeon recruitment [40]. In areas of the Columbia and Snake Rivers with hydropower development, White Sturgeon populations are functionally isolated by dams. Consequently, White Sturgeon depend on conditions within restricted reaches to sustain production. In some reaches, suitable rearing habitat exists, and individual growth rates are high, but spawning habitat is limited and recruitment of fish is poor [65]. In other reaches, favorable spawning conditions exist but growth of young fish may be density limited [69]. How the availability of food resources for larval and juvenile White Sturgeon varies among reservoirs may affect age-0 White Sturgeon recruitment. Research has also suggested that contaminants may affect White Sturgeon reproductive biology The Columbia River is the fourth largest river by volume in the United States [68] and drains a basin of 671,000 km^2 that includes parts of seven states, land ceded to 14

groups of affiliated tribes in the U.S. portion of the Columbia River Basin, land ceded to three tribal groups, known as First Nations, in the Canadian portion of the basin, and one Canadian province ([69]; Fig 1). The Columbia River supports anadromous fish species such as Chinook (*Oncorhynchus tshawytscha)*, Coho (*O. kisutch)*, Chum (*O. keta)*, and Sockeye Salmon (*O. nerka)*, including 12 populations of four species of salmon and steelhead (*O. mykiss)* listed as threatened or endangered under the ESA. The Columbia River also contains a complement of resident native and non-native fishes. Among the resident native fish species are White Sturgeon, the largest freshwater fish in North America [70]. Like other sturgeon species, anthropogenic stressors have negatively affected White Sturgeon productivity.

Our knowledge of factors affecting White Sturgeon productivity remain poorly understood [38]. Therefore, we used the CM to identify knowledge gaps associated with the hypotheses that dam construction and operation, land-use practices, and invasive species, in some combination, affect the recruitment of age-0 White Sturgeon. Within the basin, development of hydroelectric and water-storage dams have changed the magnitude and seasonality of the natural river discharge (Fig S3) and thermal regimes [71], reduced the quantity and quality of spawning habitats [72, 73], and disrupted historical migration patterns [74]. Prior to hydropower development, White Sturgeon experienced a hydrograph that peaked during June-July due to snowmelt [71]. However, from 1949 to 1993 the average discharge in June decreased from 14,000 m^3/s to 6,000 m^3/s and the maximum water temperature has increased by 1.8°C [71]. White Sturgeon likely used the natural hydrograph and thermal regime as cues to seek out optimal spawning habitats and initiate spawning [72, 75].

Factors other than river discharge and water temperature may also be affecting age-0 White Sturgeon recruitment [38]. In areas of the Columbia and Snake Rivers with hydropower

~~development, White Sturgeon populations are functionally isolated by dams. Consequently, White Sturgeon depend on conditions within restricted reaches to sustain production. In some reaches, suitable rearing habitat exists, and individual growth rates are high, but spawning habitat is limited and recruitment of fish is poor [72]. In other reaches, favorable spawning conditions exist but growth of young fish may be density limited [76]. How the availability of food resources for larval and juvenile White Sturgeon varies among reservoirs may affect age-0 White Sturgeon recruitment. Research has also suggested that contaminants may affect White Sturgeon reproductive biology~~ [~~77~~70]. The introduction of non-native fishes has clearly affected the native fish assemblage in the Columbia River [32, ~~78~~71]. Channel Catfish, Smallmouth Bass *Micropterus dolomieu*, and Walleye (*Sander vitreus*) that have been introduced into the Columbia River have all been shown to prey upon or compete with native fish species [~~78, 79~~71, 72] and may also affect White Sturgeon.

The CM (Fig ~~8~~7) provides structure to the multiple competing hypotheses and indicates how anthropogenic drivers may be affecting Tier 1, 2, and 3 EEC's. ~~We hypothesized pathways of stress arising from anthropogenic drivers that affect age-0 White Sturgeon recruitment (Fig 8).~~ Not surprisingly, the strength of our understanding of the effects of stressors on White Sturgeon was greater for relationships between life stages that are more easily sampled (e.g., adults) and that rely on data that are readily accessible as part of monitoring associated with hydropower development (e.g., discharge and water temperature) or metrics that are from combinations of these variables (e.g., estimates of White Sturgeon spawning habitat; Table S3). However, for relationships between harder to sample White Sturgeon life stages (e.g., larvae), biota that require expertise and equipment atypical of traditional fisheries assessments in large rivers (e.g., benthic macroinvertebrates), or stressors that are described by metrics that require specialized

modeling expertise (e.g., sediment transport dynamics), the existing information was insufficient or not available. For example, we identified the need to better understand the effects of channel morphology and hydraulics on benthic macroinvertebrate habitat, invertebrate production, and subsequent larval White Sturgeon production. The most certain pathways connected changes in hydrology, hydraulics, and temperature regimes to reduced spawning habitat in Tier 2, then to decreased egg quality and production at Tier 3.

~~Fig 8. Conceptual model describing the relationship of anthropogenic drivers to essential ecosystem characteristics (EECs) affecting the recruitment of White Sturgeon in the Columbia River, U.S. Essential ecosystem characteristics (EECs) are groupings of ecosystem components. Tier 1 EECs represent physical and chemical effects; fundamental measures of process that are directly affected by anthropogenic and natural drivers. Tier 2 EECs represent a broad habitat category that is intended to encompass the physical, chemical, and biological components of the riverine habitats that influence reproduction, growth and survival of biotic communities. The Tier 3 EECs represent components of the biological systems that respond to changes in the hierarchical components of the conceptual model. The strength of our understanding of how natural and anthropogenic drivers interact with habitats, biological systems, and fish in large rivers is represented by the different types of lines in the figure. Solid blue lines depict a strong understanding of the relationship, the dotted-dashed blue line represents a moderate understanding of the relationship, and the black dashed line represents a weak understanding of the relationship. The different types of lines also represent the strength of our understanding of within EEC-tier relationships.~~

Formatted: Add space between paragraphs of the same style

Our results suggest there are stressors that can affect the management goal of increasing age-0 White Sturgeon recruitment that are poorly understood and that could confound efforts to manage White Sturgeon in the Columbia River. Our characterization of the spatial and temporal scales that data should be collected at could help guide future efforts to fill data gaps to support the inferences needed to address the goal of improving recruitment of age-0 White Sturgeon (Fig 9S5).

Fig 9. The spatial and temporal scales of the management goal, the scientific inferences needed to inform the management goal, and that data collection needs to occur to support the inferences for monitoring information needs identified as requiring additional data in the case study addressing White Sturgeon recruitment in the Columbia River (see Table S3 for additional detail). A:Tier 1 EEC= Biogeochemistry/thermodynamic; Stressor= altered biogeochemical regime and Tier 2 EEC= benthic macroinvertebrate habitat; Stressors=channel stability, sediment deposition, fragmentation and Tier 2 EEC=Larval White Sturgeon habitat; Stressors=habitat fragmentation sediment deposition, water temperature and Tier 2 EEC=White Sturgeon spawning habitat; Stressors=contaminants, sediment deposition and Tier 3 EEC=White Sturgeon egg quality and production; Stressor=predation by invasive species and Tier 3 EEC=larval White Sturgeon production; Stressor=predation by invasive species and Tier 3 EEC=benthic macroinvertebrate production; Stressor=benthic macroinvertebrate habitat quantity and quality; B:Tier 1 EEC=sediment transport; Stressors=altered sediment regime, altered hydraulic regime and Tier 1 EECs=channel morphology/hydraulics, sediment transport; Stressor=altered hydraulic regime and Tier 3 EEC=primary production; Stressor=nutrient fluxes; C:Tier 1 EEC=Channel morphology/Hydraulics, Sediment transport; Stressor=altered hydraulic regime and Tier 1 EEC

Formatted: Font color: Black

~~= channel morphology/hydraulics, sediment transport; Inter-tier interaction=sediment transport dynamics; D:Tier 1 EEC=Channel morphology/Hydraulics, Sediment transport; Stressor=altered hydraulic regime and Tier 1 EEC=Channel morphology/Hydraulics, Sediment transport; Inter-tier interaction=sediment transport dynamics; E:Tier 1 EEC=Biogeochemistry/Thermodynamics; Inter-tier interaction=sediment adsorption of contaminants and nutrients; F: Tier 3 EEC=larval White Sturgeon production; Stressors=larval White Sturgeon habitat quantity and quality and Tier 3 EEC=larval White Sturgeon production, age-0 White Sturgeon recruitment; Inter-tier interaction=mortality and White Sturgeon egg quality and production; Inter-tier interaction=predation of White Sturgeon eggs by native fish and Tier 3 EECs=larval White Sturgeon production; Inter-tier interaction=predation of White Sturgeon larvae by native fish and Tier 3 EECs=all; Inter-tier interactions=trophic level interactions.~~

Upper Mississippi and Illinois Rivers

In the agricultural Midwest, basin-wide land uses affect the delivery of sediments, nutrients, and runoff to the Upper Mississippi and Illinois rivers [~~80-83~~73-76]. Within the floodplain of these two large rivers, agriculture and residential land uses often rely upon the use of levees to isolate productive or developed lands during seasonal high-flow events. Within the channel, these rivers support commercial navigation with locks and dams and river-training structures, which have dramatically altered channel morphology and hydraulics throughout the system. Together, the cumulative effects of these modifications to the basin, floodplain, and river have implications for habitat diversity and native fish biodiversity ~~[84].~~[77]. Additionally, recent invasion and expansion of non-native species, namely Silver Carp (*Hypophthalmichthys*

Formatted: Add space between paragraphs of the same style

molitrix) and Bighead Carp (*H. nobilis)*, have direct and indirect effects on native fishes that likely compound or confound stress pathways on native fish biodiversity [85, 8678, 79]. Therefore, we used the CM to explore how these primary anthropogenic drivers have likely influenced fish habitats and associated life stages (Fig 108).

Fig 108. Conceptual model of how anthropogenic drivers in the upper Mississippi and Illinois Rivers influence native fish habitats and recruitment. Essential ecosystem characteristics (EECs) are groupings of ecosystem components. Tier 1 EECs represent physical and chemical effects; fundamental measures of process that are directly affected by anthropogenic and natural drivers. Tier 2 EECs represent a broad habitat category that is intended to encompass the physical, chemical, and biological components of the riverine habitats that influence reproduction, growth, and survival of biotic communities. The Tier 3 EECs represent components of the biological systems that respond to changes in the hierarchical components of the conceptual model. The strength of our understanding of the relationships of how natural and anthropogenic drivers interact with habitats, biological systems, and fish in large rivers is represented by the different types of lines in the figure. Solid blue lines depict a strong understanding of the relationship, the dotted-dashed blue line represents a moderate understanding of the relationship, and the black dashed line represents a weak understanding of the relationship. The different types of lines also represent the strength of our understanding of within EEC-tier relationships.

Increased sediment loads in combination with altered hydraulics and morphology have resulted in high rates of sedimentation, homogeneity of depth, and loss of low-velocity, off-channel areas [87, 88].[80, 81]. A diversity of off-channel habitat conditions (i.e., increased

residence time, low velocity, warm temperatures, availability of food resources) support growth and development of larval and juvenile fishes [~~89, 90~~82, 83] and often provide important food resources for adult fishes [~~91-95~~84-88]. Further, deep, low-velocity off-channel habitats are recognized as important refugia for a wide range of fishes during high-flow events and seasonal periods of low temperatures [~~96-99~~89-92]. Loss of floodplain connectivity has eliminated the seasonal exchange of nutrients, organisms and organic matter between river and floodplain environments that support biological diversity and productivity ~~[100, 101].~~[93, 94]. Reduced availability of spawning, nursery, foraging, or overwintering habitat conditions can serve as bottlenecks to fish populations through limited larval production, reduced growth, and increased overwinter mortality. For example, high sedimentation rates have been filling backwaters in the Illinois River for decades, thus limiting the availability of overwintering conditions for fishes that bioenergetically need a deep refuge with slow water velocities. Missing year-classes in this reach, represented by truncated size structure in the Largemouth Bass (*Micropterus salmoides*) population are hypothesized to be a result of periodic winter mortality (Fig ~~S4~~S6).

The application of our CM makes clear that while the general effects of anthropogenic drivers on hydrology, sediment transport, biogeochemistry and hydraulics and morphology are well understood, there is much less known about how those effects influence the quality and availability of required habitat conditions (Tier 2, Fig ~~10~~8). Although there is likely overlap of habitat requirements among species with similar life histories, the diversity of habitat conditions necessary to support a native and diverse fish community has not been explored. Consequently, the existing information needed to assess the relationship between habitat quality and quantity, and egg production, juvenile recruitment, and adult survival of fish populations within the Upper Mississippi and Illinois rivers was categorized as insufficient to not available (Tier 3 Inter-tier

interaction, Fig ~~10~~8; Table S4). Addressing these knowledge gaps could improve the effectiveness of habitat restoration efforts focused on maintaining a diverse native fish community. The spatial and temporal scales of data collection that would support needed inferences to address restoring and maintaining native fish biodiversity and habitat quantity and quality are characterized in Fig ~~11~~S7.

~~Fig 11. The spatial and temporal scales of the management goal, the scientific inferences needed to inform the management goal, and that data collection needs to occur to support the inferences for monitoring information needs identified as requiring additional data in the case study addressing native fish biodiversity and habitat diversity in the Mississippi and Illinois rivers (see Table S4 for additional detail). A:Tier 1 EEC=Sediment transport; Stressor=altered hydraulic regime and Tier 1 EEC=Biogeochemistry/Thermodynamics; Stressor=altered biogeochemical regime and Tier 1 EEC=Biogeochemistry/Thermodynamics; Inter-tier interaction=sediment adsorption of contaminants and nutrients; B: Tier 2 EEC=adult native fish overwintering habitat; Stressors=water velocity, water temperature, dissolved oxygen, sediment deposition and Tier 2 EEC=Juvenile native fish habitat; Stressors=water depth, water velocity, water temperature, dissolved oxygen, contaminants, sediment deposition and Tier 2 EEC=Native fish spawning habitat; Stressors=water depth, water velocity, habitat fragmentation, sediment deposition, water temperature, dissolved oxygen, contaminants and Tier 3 EEC=Adult and juvenile native fish recruitment; Inter-tier interaction=mortality and Tier 3 EEC=all; Stressors=invasive species and Tier 3 EEC=all; Inter-tier interaction=trophic level interactions; C:Tier 1 EEC=Channel morphology/Hydraulics; Inter-tier interaction=channel forming processes; D:Tier 1 EEC=Channel morphology/Hydraulics, Sediment transport; Inter-tier interaction=sediment~~

Formatted: Font color: Black

~~transport dynamics; E:Tier 3 EEC=Adult native fish recruitment; Stressor=adult native fish overwintering habitat quantity and quality and Tier 3 EEC=Juvenile native fish recruitment; Stressor=juvenile native fish habitat quantity and quality and Tier 3 EEC=Native fish egg quality and production; Stressor=spawning habitat quantity and quality.~~

Similarities across case studies

We observed similarities in the stressors and interactions within EEC tiers across the four case studies. For Tier 1, an altered hydrologic regime was identified as a stressor to the ~~Hydrology~~hydrology EEC in all four rivers (Table 1). Presumably this is due to the ubiquitous effects of dams on the systems examined. However, in some rivers the altered hydrologic regime originated from other anthropogenic (e.g., water use, land use, biological community alteration) and natural (e.g., ~~Climate~~climate) drivers. Similarly, all four case studies listed an altered water temperature regime as a stressor to the ~~Biogeochemistry/Thermodynamics~~biogeochemistry/thermodynamics EEC with linkages to several anthropogenic drivers (Fig 4, 6-8; Tables S1-S4). There were also similarities across case studies with respect to the identification of interactions between Tier 1 EECs with all four case studies noting interactions between Tier 1 EEC components.

Table 1. Stressors or inter-tier interactions affecting Tier 1 Essential Ecosystem Characteristics (EEC) identified as an information need in the application of the conceptual model to case studies in the Canadian River ~~[54], Colorado River [54], Columbia River (3), and Upper Mississippi and Illinois rivers (4).~~(1), Colorado River (2), Columbia River (3), and Upper Mississippi and Illinois rivers (4). Tier 1 EECs are measurable characteristics that describe processes that can significantly alter the morphological or chemical characteristics within a river channel.

	Tier 1 EEC			
Stressor or inter-tier interaction	Hydrology	Channel Morphology/Hydraulics	Sediment Transport	Biogeochemistry/ Thermodynamics
Altered Hydrologic Regime	1, 2, 3, 4	1	1, 2	
Altered Hydraulic Regime		1, 3, 4	3	
Altered Sediment Regime			3, 4	
Altered Water Temperature Regime				1, 2, 3, 4
Altered Biogeochemical Regime				1, 3, 4
Channel forming processes		1, 2, 3, 4		
Sediment transport dynamics		1, 2, 3, 4	1, 2, 3, 4	
Sediment adsorption of contaminants and nutrients				1, 2, 3, 4

For Tier 2, there were similarities across case studies; however, the adaptation and elaboration of the components to the management goal in the case studies was apparent (Table 2). The management goal associated with the case study for the Upper Mississippi and Illinois Rivers resulted in Tier 2 EEC components (e.g., overwintering habitat) and stressors (e.g., dissolved oxygen) that were unique. Spawning habitat was identified as a Tier 2 EEC component in all the case studies and multiple stressors were identified as affecting this component in two or more of the case studies. Larval fish and invertebrate habitat were noted as Tier 2 EEC components with some similarities in stressors across case studies. Habitat fragmentation, sediment deposition, and water temperature were listed as stressors to Tier 2 EECs in all four case studies. No interactions between Tier 2 EEC components were listed for the case studies.

Table 2. Stressors affecting Tier 2 Essential Ecosystem Characteristics (EEC) identified in the application of the conceptual model to case studies in the Canadian River ~~[54], Colorado River [54],~~(1), Colorado River (2), Columbia River (3), and Upper Mississippi and Illinois rivers (4). Tier 2 EECs are broadly described as physical, chemical, or biological components of "habitat" that are hypothesized to have overall fitness consequences.

	Tier 2 EEC (habitat)				
Stressor	Spawning	Overwintering	Juvenile fish	Larval fish	Invertebrate
Altered riparian community					1
Channel stability					2, 3
Contaminants	1, 3, 4		4		
Discharge					1
Dissolved oxygen	4	4	4		
Habitat fragmentation	1, 3, 4			1, 2, 3	2, 3
Sediment deposition	3, 4	4	4	3	1, 2, 3
Turbidity				2	
Water Depth	4		4		
Water temperature	1, 2, 3, 4	4	4	1, 3	
Water velocity	3, 4	4	4		

The ~~adaption~~adaptation of Tier 3 EECs and elaboration of the biological system related to the management goal addressed by the case studies resulted in EECs that were comprised of fish life stages ranging from eggs to adult fish, primary and invertebrate production, ~~invertebrates~~ and biodiversity (Table 3). All Tier 3 EEC components, except biodiversity, were present in the four case studies. Not surprisingly, habitat quantity and quality were listed as stressors to all the EECs related to fish and invertebrates. Six of eight stressors or inter-tier interactions were listed as affecting fish larvae and five of eight were noted as affecting egg quantity and quality. In contrast to Tier 2, interactions were extensively noted between Tier 3 EECs and trophic level interspecific interactions were listed in all four case studies.

Table 3. Stressors or inter-tier interactions affecting Tier 3 Essential Ecosystem Characteristics (EEC) identified in the application of the conceptual model to case studies in the Canadian River ~~[54], Colorado River [54],~~(1), Colorado River (2), Columbia River (3), and Upper Mississippi and Illinois rivers (4). Tier 3 EECs represent components of the hypothesized biological system upon which the cascading effects of anthropogenic and natural drivers act, and interactions occur.

	Tier 3 EEC						
Stressor or inter-tier interaction	Adult fish recruitment	Juvenile fish recruitment	Larval fish production	Fish egg quality/ production	Invertebrate production	Primary production	Biodiversity
Direct mortality			1				
Predation/competition by invasive species			1, 2, 3	3			4
Habitat quantity/quality	4	4	1, 2, 3	1, 2, 3, 4	1, 2, 3		
Nutrient flux						1, 2, 3	
Trophic level interspecific interactions	1, 2, 3, 4	1, 2, 3, 4	1, 2, 3, 4	1, 2, 3, 4	1, 2, 3, 4	1, 2, 3, 4	4
Predation			3	3			
Mortality	1, 3, 4	1, 2, 3, 4	1, 2, 3				
Fish condition				1, 3, 4			

Discussion

The main objective of our exploration of CMs was to impose some structure on the complex ecosystems found in large rivers and from that structure, identify gaps in monitoring information that could inform the management of fish. Comparison across our four case studies provides some insights into large rivers and the utility of the CM to identify gaps in our understanding of factors affecting fish in large rivers.

Despite large differences in the physical and ecological contexts of the river systems, the case studies ~~also~~ demonstrated substantial commonalities in the data needed to better understand how human activities affect these systems and in the application of the CM. The general tiered structure of drivers and cascading responses through EECs worked well with the four examples. Each of the four rivers could be placed in the tiered CM to illustrate current perceptions about drivers and responses. The hierarchical CM generally increased in complexity from top to bottom. Among all rivers, there tended to be greater understanding of links from drivers to Tier 1 and Tier 2 EECs, and less understanding about linkages to Tier 3.

The strength of understanding of interactions between anthropogenic and natural drivers and EECs, and between and within EECs, varied considerably among river systems, however, resulting from both variable complexity and existing knowledge. For example, linkages from drivers to Tier 1 EECs were considered strong in the case of the Humpback Chub in the Grand Canyon, but between Tier 1 and Tier 2 only moderate. This is probably indicative of the substantial research investments in examination of physical processes in this river system

[~~102~~95]~~. A logical next step in the process of identifying and prioritizing data collection needs, once major hypothesized stressor-response pathways have been described based on expert elicitation, is to conduct an extensive literature review to support or refute those pathways and the strength and understanding of the pathways.~~.

Although we did not prescribe a specific approach to the CM process, the case studies employed similar strategies. Our modelling exercises started with the definition of a management goal. In all our case studies, the management goals pertained to a desired biological endpoint represented in Tier 3. After the definition of the management goal, we conceptualized interactions between drivers and EECs and between EECs with a combination of top-down and bottom-up approaches. A top-to-bottom approach to working with these models is generally consistent with a management perspective wherein anthropogenic drivers that are most directly managed in a large-river system (e.g., land and water use, etc.) cascade from top to bottom through fluxes to physical and chemical habitats, and then to biological responses. While this is generally true for anthropogenic drivers, a notable exception to the top-to-bottom management approach would be that in the U.S., there are few actions currently directed at reducing emissions affecting climate ~~[103]~~[96] which is a natural driver in our CM. Climate was hypothesized to be a stressor in the case study application of the CM to Arkansas River Shiner management in the South Canadian River and is hypothesized to be affecting hydrologic regimes elsewhere ~~[104, 105].~~[97, 98], but was not specifically mentioned in other case studies. The CMs can readily be modified to incorporate other factors or pathways (e.g., climate effects) as new information or perspectives become available. A bottom to top approach is equally or more valuable as it starts with the foundation of understanding about the species or community, and then seeks to identify which stressors affect population or community responses. A bottom-up approach can readily

identify information gaps in linkages from ecological processes to demographic parameters [106].[99].

The top-to-bottom and bottom-up approaches meet in the middle in Tier 2 in the concept of habitat: the resources and conditions present in an area that produce occupancy [107].[100]. Tier 2 is critical as it has little value if it is not defined based on biological requirements or if managers lack understanding on how habitat is formed. Among our examples, the Upper Mississippi River is notable for asserting strong understanding of the linkages from land-use stressors to sediment regime to diminished overwintering habitat for native adult fishes. After that, interactions with other processes and life stages combine to increase uncertainty about whether overwintering habitat is a limiting factor in biodiversity. In contrast, the high confidence in understanding how White Sturgeon egg quality and production are linked to spawning habitat in the Columbia River Basin, provides a strong linkage upward through Tier 1 EECs and potential management actions (Fig 87). Although at times elusive, the concept of habitat is critical for linking management to biotic endpoints [108101].

Field Code Changed

Large rivers are typically managed for multiple objectives, including fisheries, multi-species, or ecosystem objectives. Management decisions typically require an understanding of how management actions propagate through a river ecosystem. Although the emphasis may be on a biological endpoint (among other objectives), understanding the intermediate steps and the processes linking them, and potential interactions between processes or EEC components, can help formulate effective management strategies; especially as multiple objectives compete. In a multi-species context, the conceptual models can help identify commonalities and differences in in how stressors propagate to biota and therefore provide a basis for prioritizing monitoring efforts. In the case where species or guilds have similar habitat affinities and life histories, a

dominant anthropogenic stress pathway may be hypothesized and focus on a single or few monitoring components may be justified. An example may be multiple large-river species that are known to be cued to spawn by spring flow pulses. In such a case, the characteristics of the annual hydrograph would be a dominant physical monitoring variable and biological monitoring could focus on reproductive success of one or more of the species. In the case where multiple species of concern have different reproductive strategies – for example, rheophilic species like sturgeon that may require in-channel dispersion of young to flowing habitats compared to invasive carp whose young thrive when they can disperse to lentic floodplain pools – pathways and monitoring strategies will diverge. In the latter case, it would probably not be sufficient to monitor and assess the characteristics of the annual hydrograph; instead, hydrologic metrics would need to be integrated with hydraulic and geomorphic metrics to assess where and when the different habitats would be available and could be targeted for young-of-the-year sampling. Effectively addressing multiple species would rely on detailed knowledge of life histories and how they play out on the landscape – such information is missing for many species and may need to be developed for effective design of monitoring and management actions.

Management actions intended to benefit fish in large rivers can directly or indirectly affect multiple ecosystem components. Without consideration of the effects of management on non-target ecosystem components, unintended consequences may limit management efficacy. Hypothesizing inter-tier interactions in the Tier 3 EEC (e.g., see Fig 7), can provide insight on the potential interactions among fish species and other biological components in the context of the hierarchical CM. In all our case studies, the lumping of multiple biological interactions in Tier 3 resulted in a simplification of complex trophic interactions. For example, as Tier 3 encompasses all biological responses, it includes multiple life stages of many interacting species

at varying trophic levels. Because of this, the four CMs diverged significantly at Tier 3 as components were expanded to accommodate existing understanding. Even as the Tier 3 components were expanded in complexity, they remained highly simplified views of the ecosystem. Simplification was based, in part, on the importance of key species in management goals and the experts' existing knowledge. Even though the hypothesized Tier 3 interactions in our case studies conveyed a simplification of the trophic interactions, the hypothesized interactions do suggest the need for information that clarifies the trophic interactions and effects of Tier 3 EEC components on the biological endpoint. If desired or warranted, the Tier 3 EEC could be elaborated to capture more complexity. For example, in Fig 7, the Tier 3 inter-tier interaction between anadromous and resident fishes and white sturgeon larvae could expanded to include interactions with specific fish species. Monitoring can help clarify the effects of management actions, including on non-target ecosystem components, but only if data are collected to characterize key ecosystem processes that could affect the outcome. The process of considering and elucidating Tier 3 EEC interactions can help identify the non-target ecosystem components that could be affected when managing for a specific biological endpoint.

~~The CMs explored here also provide a framework for considering return on science investments.~~ The CMs explored here also provide a framework for considering return on science investments. ~~Large rivers are typically managed for multiple objectives, including fisheries, multi-species, or ecosystem objectives. Management decisions typically require an understanding of how management actions propagate through a river ecosystem. Although the emphasis may be on a biological endpoint (among other objectives), understanding the intermediate steps and the processes linking them, and potential interactions between processes or EEC components, can help formulate effective management strategies; especially as multiple~~

~~objectives compete. For example, to increase recruitment of a fish species, it is not enough to know that increased reservoir-flow releases are associated with increased spawning unless we understand how much flow, when, for how long, and at what temperature, are needed to optimize other EECs to increase survival and recruitment.~~

The knowledge needed for effective management of large rivers can be gained by monitoring intermediate endpoints along the cascade, but the type of information and costs vary widely. Costs for monitoring Tier 1 EECs can be high but some programs are already in place. For example, large rivers are likely to have monitoring infrastructure installed for Tier 1 monitoring of discharge and temperature regimes, with varying potential for monitoring sediment transport and water quality. Investment at Tier 2 may emphasize physical processes and habitats that can be measured at relatively low cost, assuming that habitats are adequately defined based on biological criteria. In larger rivers, Tier 2 habitat assessments can be more cost effective compared to smaller rivers because they can rely on automated data collection through hydroacoustics and remote sensing ~~[108].~~[101]. As discussed above, habitat assessments have value only to the extent that they are based on well-defined biological requirements; it is notable that some large-river management efforts have found that relatively simple habitat models are useful to predict biological responses [~~109~~102]. At Tier 3, costs can increase substantially because of structural uncertainties (i.e., which life stages, which species are most important to monitor) and because of the inherent uncertainties of monitoring fish in large river systems where detection probability can be low and highly variable [36, ~~110~~103]~~. The CMs presented here can be used to assess which pathways are thought to be most important, and which components of EECs would yield the best return on investment for decision making.~~. Generally, the cost of monitoring increases from Tier 1 to Tier 3 in the CM hierarchy; at the same time, the

relevance of information to decision making is typically greater for biological responses depicted in Tier 3 [104].

Because both costs and information benefits increase from Tier 1 to Tier 3 in the CM hierarchy, it is difficult to generalize about where the benefit:cost ratio would be optimized. Indeed, as discussed by Jacobson and Berkley [30], the decision about where in the hierarchy monitoring resources would get the highest return on investment may depend more directly on managers' and stakeholders' perceptions about risks of acting with incomplete information. For example, the details of how a fish's reproductive strategy depends on the nuances of a seasonal hydrograph may not be known, but stakeholders may believe strongly that the natural hydrograph was functional for the species and therefore monitoring of the flow regime will have the highest return on investment and, by extension, restoration of the flow regime is likely to have the most positive effects. On the other hand, in systems where stakeholders opinions are divided or socio-economic values would be compromised by a return to a natural flow regime, managers may be required to demonstrate more precisely how elements of the flow regime propagate to species' benefits [105]. Thus, once information needs are identified and there is an assessment of the availability of data identified as information needs, there needs to be a process whereby the costs of collecting the information need to be placed in a socioeconomic context (e.g., see [30]).

The development of the CMs described in this manuscript can be a first step in application of structured decision-making (SDM) and its iterative form-adaptive management (AM) processes [106, 107]. Structured decision-making is a stakeholder driven process by which a problem can be defined with conceptual models and decomposed into decision components that include the problem context, stakeholder objectives, potential management actions,

consequences of those actions on the objectives, and trade-offs related to different decisions (actions) [107-109]. One primary focus of SDM is the identification of uncertainties such as those identified in the CMs for the case studies in this paper [110]. Quantification of the influence of decision relevant uncertainties can be modeled using sensitivity analysis and other techniques and ranked [107, 109, 111]. In addition, the quantitative techniques available to assist in solving complex ecological problems are robust and range in complexity from consequences tables to Bayesian models to dynamic optimization models [107-109, 112, 113]. The SDM process is often used as the set-up phase for adaptive management which includes monitoring over time to reduce uncertainty related to how management will influence important outcomes (e.g. fish population status; [109, 112].

The CM may also help to identify which processes or components are amenable to a field monitoring effort and which are more aptly addressed through laboratory or mesocosm experiments. For example, if it is hypothesized that the condition of age 1+ Arkansas Shiners is a critical determining factor in egg quality or production (Fig ~~5~~4), it could be determined that the best approach to developing a quantitative relation between condition and eggs is through a controlled laboratory experiment rather than field-based monitoring. The CM helps to visualize where different types of information may be applied within a decision-making framework.

A large-river CM may also serve as a precursor to computational ecological or population models ~~[30]~~[30]. Similar questions about how monitoring and other science efforts should be distributed among EECs and processes can be addressed iteratively by carrying out sensitivity analyses in a modeling framework. ~~-~~Indeed, given substantial uncertainties associated with monitoring data, computation modeling can be considered a necessary component of large-river monitoring and evaluation systems [~~106, 111~~99, 114].

Conclusions

We found the process of conceptualizing the relationships between and within EECs fostered a critical assessment of what we know about factors affecting the management endpoint ~~being considered.~~. By visualizing how EEC drivers directly and indirectly affect management endpoints, our CM identified critical information gaps and uncertainties that, if resolved, could improve our understanding of how to best meet management objectives. The process of conceptualizing the EEC relationships affecting fish in large rivers could help to structure, or restructure, monitoring programs around scientifically sound monitoring questions, promote the selection of relevant ecological indicators that characterize resource condition or management outcomes, and facilitate communication and information sharing within and between organizations managing or researching management endpoints. Ultimately, understanding the mechanisms by which EECs influence large-river fishes will improve the effectiveness of restoration and management actions.

As shown with our case studies, our CM is flexible and applicable to a wide range of river systems with different anthropogenic drivers and management objectives. We feel our CM provides a generic structure that scientists can adapt to their management goals and needs. By not being overly prescriptive, for example, with respect to the components of the Tier 2 and 3 EEC components, scientists can adapt the CM to different biological communities and management endpoints. By doing so, we feel that users have the flexibility to place their management questions in the context of EECs that are specific to their large-river system.

Although the case studies addressed management issues that were river or basin specific, there were similarities relative to information needs and data availability. For example, in most

systems information on river discharge and water temperature were needed and available. Conversely, information regarding trophic relationships and the habitat requirements of larval fishes were generally lacking. This result suggests that there may be a common need for a better understanding of certain factors across large-river systems.

Acknowledgements

We thank Megan Dethloff of the Pacific Northwest Aquatic Monitoring Partnership for her role in organizing the logistics associated with the workshop and conference calls needed to develop this manuscript. David Ward from the U.S. Geological Survey's Grand Canyon Monitoring and Research Center provided many valuable comments and suggestions that improved the manuscript. This work was funded in part by U.S. Geological ~~Survey's Core~~Survey, Science ~~Systems Mission Area~~Synthesis, Analysis, and Research Program. This research is a contribution of the Ball State University, Illinois Natural History Survey, Oregon Department of Fish and Wildlife, Pacific Northwest Aquatic Monitoring Partnership, the ~~Oklahoma and~~ Alabama Cooperative Fish and Wildlife Research ~~Units~~Unit, and the U.S. Geological Survey Columbia Environmental Research Center, Grand Canyon Monitoring and Research Center, Oregon Water Science Center, Upper Midwest Environmental Sciences Center, and Western Fisheries Research Center. Any use of trade, firm, or product names is for descriptive purposes only and does not imply endorsement by the U.S. Government. An animal care and use protocol was not required for this research.

References

1. Thomas JA, Emery EB, McCormick FH. Detection of temporal trends in Ohio River fish assemblages based on lockchamber surveys (1957–2001). In: J.N. Rinne, R.M. Hughes, Calamusso B, editors. Historical changes in large river fish assemblages of the Americas. 45. Bethesda, MD: American Fisheries Society; 2005. p. 431-49.
2. Lohner TW, Dixon DA. The value of long-term environmental monitoring programs: an Ohio River case study. Environ Monit Assess. 2013;185(11):9385-96.
3. McClelland MA, Sass GG, Cook TR, Irons KS, Michaels NN, O'Hara TM, et al. The long-term Illinois River fish population monitoring program. Fisheries. 2012;37(8):340-50. doi: 10.1080/03632415.2012.704815. .
4. Ward JV. Riverine landscapes: Biodiversity patterns, disturbance regimes, and aquatic conservation. Biol Conserv. 1998;83(3):269-78. PubMed PMID: WOS:A1998YJ19500005.
5. Lee LS, Garnett JA, Bright EG, Sharitz RR, Batzer DP. Vegetation, invertebrate, and fish community response to past and current flow regulation in floodplains of the Savannah River, Southeastern USA. Wetlands Ecol Manage. 2016;24:443-55. doi: 10.1007/s11273-015-9470-y.
6. Lowe WH, Likens GE, Power ME. Linking scales in stream ecology. Bioscience. 2006;56(7):591-7. PubMed PMID: WOS:000239017800010.
7. Fausch KD, Torgersen CE, Baxter CV, Li HW. Landscapes to riverscapes: Bridging the gap between research and conservation of stream fishes. Bioscience. 2002;52(6):483-98. PubMed PMID: WOS:000176233800009.
8. Allan JD. Landscapes and riverscapes: The influence of land use on stream ecosystems. Annu Rev Ecol Evol S. 2004;35:257-84. PubMed PMID: WOS:000226244100010.
9. Steel EA, Hughes RM, Fullerton AH, Schmutz S, Young JA, Fukushima M, et al. Are we meeting the challenges of landscape-scale riverine research? A review. Living Reviews in Landscape Research. 2010;4.
10. Thoms M, Sheldon F. Large rivers as complex adaptive ecosystems. River Res Appl. 2019;35(5):451-8. doi: 10.1002/rra.3448. PubMed PMID: WOS:000470931600001.
11. Nilsson C, Reidy CA, Dynesius M, Revenga C. Fragmentation and Flow Regulation of the World's Large River Systems. Science. 2005;15:405-8.
12. Harding JS, Benfield EF, Bolstad PV, Helfman GS, Jones EBD. Stream biodiversity: The ghost of land use past. ~~Proceedings of the National Academy of Sciences of the United States of America.~~P Natl Acad Sci USA. 1998;95(25):14843-7. PubMed PMID: WOS:000077436700047.
13. King AJ, Gawne B, Beesley L, Koehn JD, Nielsen DL, Price A. Improving Ecological Response Monitoring of Environmental Flows Environmental Management. 2015;55:991-1005.
14. Gawne B, Hale J, Stewardson MJ, Webb JA, Ryder DS, Brooks SS, et al. Monitoring of environmental flow outcomes in a large river basin: The Commonwealth Environmental Water Holder's long-term intervention in the Murray-Darling Basin, Australia. River Res Appl. 2020;36(4):630-44. doi: 10.1002/rra.3504. PubMed PMID: WOS:000484205300001.
15. Schletterer M, Shaporenko SI, Kuzovlev VV, Minin AE, Van Geest GJ, Middelkoop H, et al. The Volga: Management issues in the largest river basin in Europe. River Res Appl. 2019;35(5):510-9. doi: 10.1002/rra.3268. PubMed PMID: WOS:000470931600007.
16. Lindenmayer DB, Likens GE. The science and application of ecological monitoring. Biol Conserv. 2010;143(6):1317-28. PubMed PMID: WOS:000278572300002.

17. Worthington TA, Brewer SK, Grabowski TB, Mueller J. Backcasting the decline of a vulnerable Great Plains reproductive ecotype: Identifying threats and conservation priorities. Global Change Biology. 2014;20:89-102.

18. Worthington TA, Echelle AA, Perkin JS, Mollenhauer R, Farless N, Dyer JJ, et al. The emblematic minnows of the North American Great Plains: A synthesis of threats and conservation opportunities. Fish ~~Fish~~and Fisheries. 2018;19(2):271-307. doi: 10.1111/faf.12254. PubMed PMID: WOS:000426503000006.

19. Noon BR, Spies TA, Raphael MG. Conceptual basis for designing an effectiveness monitoring plan. The strategy and designing of the effectiveness program for the Northwest Forest Plan1999. p. 21-48.

20. Fancy SG, Gross JE, Carter SL. Monitoring the condition of natural resources in US national parks. Environ Monit Assess. 2009;151(1-4):161-74. PubMed PMID: WOS:000263832800014.

21. Irvine KM, Miller SW, Al-Chokhachy RK, Archer EK, Roper BB, Kershner JL. Empirical evaluation of the conceptual model underpinning a regional aquatic long-term monitoring program using causal modelling. Ecol Indic. 2015;50:8-23. PubMed PMID: WOS:000348265200002.

22. Hein T, Funk A, Pletterbauer F, Graf W, Zsuffa I, Haidvogl G, et al. Management challenges related to long-term ecological impacts, complex stressor interactions, and different assessment approaches in the Danube River Basin. River Res Appl. 2019;35(5):500-9. doi: 10.1002/rra.3243. PubMed PMID: WOS:000470931600006.

23. U.S. Columbia Basin Tribes and Canadian First Nations. Fish passage and reintroduction into the US and Canadian upper Columbia River. *An Interim Joint Paper of the US Columbia Basin Tribes and Canadian First Nations February*. 2014.

24. Carlisle DM, Meador MR, Short TM, Tate CM, Gurtz ME, Bryant Jr WL, et al. The quality of our Nation’s waters--ecological health in the Nation's streams, 1993-2005. US Geological Survey Circular 1391. 2013.

25. Sedeño-Díaz JE, López-López E. Freshwater fish as sentinel organisms: From the molecular to the population level, a review. In: Turker H, editor. New Advances and Contributions to Fish Biology: IntechOpen; 2012.

26. Agency USEP. Rivers and streams assessment 2008-2009 A collaborative survey DRAFT (EPA/841/D-13/001). Washington, DC Office of Wetlands, Oceans and Watersheds Office of Research and Development.; 2013.

27. DeBoer JA, Thoms MC, Casper AF, Delong MD. The Response of Fish Diversity in a Highly Modified Large River System to Multiple Anthropogenic Stressors. J Geophys Res-Biogeo. 2019;124(2):384-404. doi: 10.1029/2018jg004930. PubMed PMID: WOS:000460859100013.

28. Collier KJ, Baker C, David BO, Gorski K, Pingram MA. Linking ecological science with management outcomes on New Zealand's longest river. River Res Appl. 2019;35(5):476-88. doi: 10.1002/rra.3181. PubMed PMID: WOS:000470931600004.

29. Harwell MA, Myers V, Young T, Bartuska A, Gassman N, Gentile JH, et al. A framework for an ecosystem integrity report card. Bioscience. 1999;49(7):543-56. PubMed PMID: WOS:000081211600008.

30. Jacobson RB, Berkley J. Conceptualizing and communicating ecological river restoration. In: Simon A, Bennett SJ, Castro JM, editors. Stream Restoration in Dynamic Fluvial

Systems. Geophysical Monograph Series. Washington, D.C.: American Geophysical Union; 2011. p. 9-27.

31. Ward DL, Casper AF, Counihan TD, Bayer JM, Waite IR, Kosovich JJ, et al. Long-Term Fish Monitoring in Large Rivers: Utility of "Benchmarking" across Basins. Fisheries. 2017;42(2):100-14. PubMed PMID: WOS:000394751100007.

32. Counihan TD, Waite IR, Casper AF, Ward DL, Sauer JS, Irwin ER, et al. Can data from disparate long-term fish monitoring programs be used to increase our understanding of regional and continental trends in large river assemblages? Plos One. 2018;13(1). PubMed PMID: WOS:000423412500050.

33. Carpenter SR, Bennett EM, Peterson GD. Special feature on scenarios for ecosystem services. Ecol Soc. 2006;11(2). PubMed PMID: WOS:000243280800002.

34. Tonn WM. Climate Change and Fish Communities - a Conceptual-Framework. Transactions of the American Fisheries Society. 1990;119(2):337-52. PubMed PMID: WOS:A1990DV01100015.

35. Rosenfeld J. Assessing the habitat requirements of stream fishes: An overview and evaluation of different approaches. Transactions of the American Fisheries Society. 2003;132(5):953-68. PubMed PMID: WOS:000185446500011.

36. Mollenhauer R, Brewer SK. Multinomial N-Mixture Models Improve the Applicability of Electrofishing for Developing Population Estimates of Stream-Dwelling Smallmouth Bass. North American Journal of Fisheries Management. 2017;37(1):211-24. PubMed PMID: WOS:000394648700021.

37. Grill G, Lehner B, Lumsdon AE, MacDonald GK, Zarfl C, Liermann CR. An index-based framework for assessing patterns and trends in river fragmentation and flow regulation by global dams at multiple scales. Environmental Research Letters. 2015;10(1):015001.

38. van Puijenbroek PJ, Buijse AD, Kraak MH, Verdonschot PF. Species and river specific effects of river fragmentation on European anadromous fish species. River Res Appl. 2019;35(1):68-77.

39. Wolter C, Buijse AD, Parasiewicz P. Temporal and Spatial Patterns of Fish Response to Hydromorphological Processes. River Res Appl. 2016;32(2):190-201. doi: 10.1002/rra.2980. PubMed PMID: WOS:000369979000005.

~~38~~40. Counihan TD, Chapman CG. Relating river discharge and water temperature to the recruitment of age-0 White Sturgeon (Acipenser transmontanus Richardson, 1836) in the Columbia River using over-dispersed catch data. J Appl Ichthyol. 2018;34(2):279-89. PubMed PMID: WOS:000428797200004.

~~39~~41. Halvorson LJ, Cady BJ, Kappenman KM, James BW, Webb MAH. Observations of handling trauma of Columbia River adult white sturgeon, Acipenser transmontanus Richardson, 1836, to assess spawning sanctuary success. J Appl Ichthyol. 2018;34(2):390-7.

~~40~~42. Jay DA, Naik PK. Distinguishing human and climate influences on hydrological disturbance processes in the Columbia River, USA. Hydrolog Sci J. 2011;56(7):1186-209. doi: 10.1080/02626667.2011.604324. PubMed PMID: WOS:000297289600008.

~~41~~43. Omernik JM, Griffith GE, Hughes RM, Glover JB, Weber MH. How Misapplication of the Hydrologic Unit Framework Diminishes the Meaning of Watersheds. Environmental Management. 2017;60(1):1-11. doi: 10.1007/s00267-017-0854-z. PubMed PMID: WOS:000402792800001.

~~42~~44. U.S. Geological Survey, U.S. Department of Agriculture - Natural Resources Conservation Service. Federal Standards and Procedures for the National Watershed Boundary Dataset (WBD) (4 ed.). U.S. Geological Survey Techniques and Methods 11–A3; 2013. p. 63.

~~43~~45. Frissell CA, Liss WJ, Warren CE, Hurley MD. A Hierarchical Framework for Stream Habitat Classification - Viewing Streams in a Watershed Context. Environmental Management. 1986;10(2):199-214. doi: Doi 10.1007/Bf01867358. PubMed PMID: WOS:A1986A793400006.

~~44~~46. Simenstad CA, Burke JL, O'Connor JE, Cannon C, Heatwole DW, Ramirez MF, et al. Columbia River Estuary Ecosystem Classification—Concept and Application. U.S. Geological Survey Open-File Report 2011-1228; 2011. p. 54.

~~45~~47. Levin SA, Paine RT. Disturbance, patch formation, and community structure. ~~Proceedings of the National Academy of Sciences of the United States of America.~~P Natl Acad Sci USA. 1974;71(7):2744-7.

~~46~~48. Pringle CM, Naiman RJ, Bretschko G, Karr JR, Oswood MW, Webster JR, et al. Patch Dynamics in Lotic Systems - the Stream as a Mosaic. J N Am Benthol Soc. 1988;7(4):503-24. doi: Doi 10.2307/1467303. PubMed PMID: WOS:A1988R878400011.

~~47. Woods AJ, Omernik JM, Butler DR, Ford JG, Henley JE, Hoagland BW, et al. Ecoregions of Oklahoma (color poster with map, descriptive text, summary tables, and photographs). Reston, Virginia: U.S. Geological Survey; 2005.~~

~~48. Smith SJ, Ellis JH, Wagner DL, Peterson SM. Hydrogeology and simulated groundwater flow and availability in the North Fork Red River aquifer, southwest Oklahoma, 1980–2013. U.S. Geological Survey Scientific Investigations Report 2017–5098; 2017. p. 107.~~

~~49. Perkin JS, Gido KB, Falke JA, Fausch KD, Crockett H, Johnson ER, et al. Groundwater declines are linked to changes in Great Plains stream fish assemblages. Proceedings of the National Academy of Sciences of the United States of America. 2017;114(28):7373-8. doi: 10.1073/pnas.1618936114. PubMed PMID: WOS:000405177100073.~~

~~50. Fox GA. Evaluation of a stream aquifer analysis test using analytical solutions and field data. J Am Water Resour As. 2004;40(3):755-63. doi: DOI 10.1111/j.1752-1688.2004.tb04457.x. PubMed PMID: WOS:000222484600015.~~

~~51. Fox GA, Heeren DM, Miller RB, Mittelstet AR, Storm DE. Flow and transport experiments for a streambank seep originating from a preferential flow pathway. Journal of Hydrology. 2011;403(3-4):360-6. doi: 10.1016/j.jhydrol.2011.04.014. PubMed PMID: WOS:000291914200014.~~

~~52. Warren ML, Burr BM, Walsh SJ, Bart HL, Cashner RC, Etnier DA, et al. Diversity, distribution, and conservation status of the native freshwater fishes of the southern United States. Fisheries. 2000;25(10):7-31. doi: Doi 10.1577/1548-8446(2000)025<0007:Ddacso>2.0.Co;2. PubMed PMID: WOS:000089569600002.~~

~~53~~49. Worthington TA, Brewer SK, Farless N, Grabowski TB, Gregory MS. Interacting Effects of Discharge and Channel Morphology on Transport of Semibuoyant Fish Eggs in Large, Altered River Systems. Plos One. 2014;9(5). doi: 10.1371/journal.pone.0096599. PubMed PMID: WOS:000338029800091.

~~54~~50. Wilde GR. Migration of Arkansas River Shiner and other Broadcast Spawning Fishes in the Canadian River, New Mexico-Texas. Albuquerque, New Mexico2016.

~~55~~51. Hoagstrom CW, Turner TF. Recruitment ecology of pelagic-broadcast spawning minnows: paradigms from the ocean advance science and conservation of an imperilled

freshwater fauna. Fish ~~Fish~~and Fisheries. 2015;16(2):282-99. doi: 10.1111/faf.12054. PubMed PMID: WOS:000352536200005.

~~56~~52. Birken AS, Cooper DJ. Processes of Tamarix invasion and floodplain development along the lower Green River, Utah. Ecol Appl. 2006;16(3):1103-20. doi: Doi 10.1890/1051-0761(2006)016[1103:Potiaf]2.0.Co;2. PubMed PMID: WOS:000238451500023.

~~57~~53. Nagler PL, Glenn EP, Jarnevich CS, Shafroth PB. Distribution and Abundance of Saltcedar and Russian Olive in the Western United States. Crit Rev Plant Sci. 2011;30(6):508-23. doi: 10.1080/07352689.2011.615689. PubMed PMID: WOS:000298323800002.

~~58~~54. Wilde GR, Bonner TH, Zwank PJ. Diets of the Arkansas River shiner and peppered chub in the Canadian River, new Mexico and Texas. J Freshwater Ecol. 2001;16(3):403-10. doi: Doi 10.1080/02705060.2001.9663829. PubMed PMID: WOS:000171102500011.

~~59~~55. Shafer M, Ojima D, Antle JM, Kluck D, McPherson RA, Petersen S, et al. Ch. 19: Great Plains. In: Melillo JM, Richmond TC, Yohe GW, editors. Climate Change Impacts in the United States: The Third National Climate Assessment: U.S. Global Change Research Program; 2014. p. 441-61.

~~60~~56. Otkin JA, Anderson MC, Hain C, Svoboda M, Johnson D, Mueller R, et al. Assessing the evolution of soil moisture and vegetation conditions during the 2012 United States flash drought. Agr Forest Meteorol. 2016;218:230-42. doi: 10.1016/j.agrformet.2015.12.065. PubMed PMID: WOS:000370905100022.

~~61~~57. Worthington TA, Zhang TJ, Logue DR, Mittelstet AR, Brewer SK. Landscape and flow metrics affecting the distribution of a federally-threatened fish: Improving management, model fit, and model transferability. Ecol Model. 2016;342:1-18. doi: 10.1016/j.ecolmodel.2016.09.016. PubMed PMID: WOS:000388154100001.

~~62. Olden JD, Poff NL, Bestgen KR. Life-history strategies predict fish invasions and extirpations in the Colorado River Basin. Ecol Monogr. 2006;76(1):25-40. PubMed PMID: WOS:000236562100003.~~

~~63~~58. Gorman OT, Stone DM. Ecology of spawning humpback chub, Gila cypha, in the Little Colorado River near Grand Canyon, Arizona. Environ Biol Fish. 1999;55(1-2):115-33. PubMed PMID: WOS:000081503900011.

~~64~~59. Childs MR, Clarkson RW, Robinson AT. Resource use by larval and early juvenile native fishes in the Little Colorado River, Grand Canyon, Arizona. Transactions of the American Fisheries Society. 1998;127(4):620-9. PubMed PMID: WOS:000075350600009.

~~65~~60. Converse YK, Hawkins CP, Valdez RA. Habitat relationships of subadult humpback chub in the Colorado River through Grand Canyon: Spatial variability and implications of flow regulation. Regul River. 1998;14(3):267-84. PubMed PMID: WOS:000074199700004.

~~66~~61. Petersen JH, Paukert CP. Development of a bioenergetics model for humpback chub and evaluation of water temperature changes in the Grand Canyon, Colorado River. Transactions of the American Fisheries Society. 2005;134(4):960-74. PubMed PMID: WOS:000230411700015.

~~67~~62. Marsh PC, Douglas ME. Predation by introduced fishes on endangered humpback chub and other native species in the Little Colorado River, Arizona. Transactions of the American Fisheries Society. 1997;126(2):343-6. PubMed PMID: WOS:A1997WW21700018.

~~68. Kammerer JC. Largest rivers in the United States: U.S. Geological Survey Open-File Report OFR 87-242. 1987. p. 2.~~

~~69. Ebel WJ, Becker CD, Mullan JW, Raymond HL. The Columbia River: toward a holistic understanding. Canadian special publication of fisheries and aquatic sciences. 1989;106:205-19.~~

~~70~~63. Page LM, Burr BM. A field guide to freshwater fishes of North America north of Mexico. Boston, MA: Houghton Mifflin Harcourt; 2011.

~~71~~64. Quinn TP, Adams DJ. Environmental changes affecting the migratory timing of American shad and sockeye salmon. Ecology. 1996;77(4):1151-62. PubMed PMID: WOS:A1996UN89400018.

~~72~~65. Parsley MJ, Beckman LG. White sturgeon spawning and rearing habitat in the lower Columbia River. North American Journal of Fisheries Management. 1994;14:812-27.

~~73~~66. Chapman CG, Jones TA. First Documented Spawning of White Sturgeon in the Lower Willamette River, Oregon. Northwest Sci. 2010;84(4):327-35. PubMed PMID: WOS:000285797800002.

~~74~~67. North JA, Beamesderfer RC, Rien TA. Distribution and Movements of White Sturgeon in 3 Lower Columbia River Reservoirs. Northwest Sci. 1993;67(2):105-11. PubMed PMID: WOS:A1993LE90500006.

~~75~~68. Perrin CJ, Rempel LL, Rosenau ML. White sturgeon spawning habitat in an unregulated river: Fraser River, Canada. Transactions of the American Fisheries Society. 2003;132(1):154-65. PubMed PMID: WOS:000180362000016.

~~76~~69. Beamesderfer RCP, Rien TA, Nigro AA. Differences in the Dynamics and Potential Production of Impounded and Unimpounded White Sturgeon Populations in the Lower Columbia River. Transactions of the American Fisheries Society. 1995;124(6):857-72. PubMed PMID: WOS:A1995TF91800007.

~~77~~70. Feist GW, Webb MAH, Gundersen DT, Foster EP, Schreck CB, Maule AG, et al. Evidence of detrimental effects of environmental contaminants on growth and reproductive physiology of white sturgeon in impounded areas of the Columbia River. Environ Health Persp. 2005;113(12):1675-82. PubMed PMID: WOS:000233713200035.

~~78~~71. Sanderson BL, Barnas KA, Rub AMW. Nonindigenous Species of the Pacific Northwest: An Overlooked Risk to Endangered Salmon? Bioscience. 2009;59(3):245-56. PubMed PMID: WOS:000263863400009.

~~79~~72. Zimmerman MP. Food habits of smallmouth bass, walleyes, and northern pikeminnow in the lower Columbia River Basin during outmigration of juvenile anadromous salmonids. Transactions of the American Fisheries Society. 1999;128(6):1036-54. PubMed PMID: WOS:000089184000005.

~~80~~73. Knox JC. Agricultural influence on landscape sensitivity in the Upper Mississippi River Valley. Catena. 2001;42(2-4):193-224.

~~81~~74. Turner RE, Rabalais NN. Linking landscape and water quality in the Mississippi River Basin for 200 years. ~~BioScience~~Bioscience. 2003;53(6):563-72.

~~82~~75. Houser JN, Richardson WB. Nitrogen and phosphorus in the Upper Mississippi River: transport, processing, and effects on the river ecosystem. Hydrobiologia. 2010;640(1):71-88.

~~83~~76. Schilling KE, Chan KS, Liu H, Zhang YK. Quantifying the effect of land use land cover change on increasing discharge in the Upper Mississippi River. Journal of Hydrology. 2010;387(3-4):343-5.

~~84~~77. Bouska KL. Discontinuities and functional resilience of large river fish assemblages. Ecosphere. 2018;9(7). doi: 10.1002/ecs2.2351. PubMed PMID: WOS:000441526900003.

~~85~~78. Pendleton RM, Schwinghamer C, Solomon LE, Casper AF. Competition among river planktivores: are native planktivores still fewer and skinnier in response to the Silver Carp

invasion? Environ Biol Fish. 2017;100(10):1213-22. doi: 10.1007/s10641-017-0637-7. PubMed PMID: WOS:000412840700005.

~~86~~79. Solomon LE, Pendleton RM, Chick JH, Casper AF. Long-term changes in fish community structure in relation to the establishment of Asian carps in a large floodplain river. Biol Invasions. 2016;18(10):2883-95. doi: 10.1007/s10530-016-1180-8. PubMed PMID: WOS:000382847100012.

~~87~~80. Bhowmik NG, Demissie M. Sedimentation in the Illinois River valley and backwater lakes. Journal of Hydrology. 1989;105(1-2):187-95.

~~88~~81. WEST Consultants Inc. Upper Mississippi River and Illinois Waterway Cumulative Effects Study Rock Island, Illinois, USA: Department of the Army, Corps of Engineers, 2000.

~~89~~82. Nannini MA, Goodrich J, Dettmers JM, Soluk DA, Wahl DH. Larval and early juvenile fish dynamics in main channel and backwater lake habitats of the Illinois River ecosystem. Ecol Freshw Fish. 2012;21(4):499-509. PubMed PMID: WOS:000308290000004.

~~90~~83. Love SA, Phelps QE, Tripp SJ, Herzog DP. The Importance of Shallow-Low Velocity Habitats to Juvenile Fish in the Middle Mississippi River. River Res Appl. 2017;33(3):321-7. PubMed PMID: WOS:000397876900002.

~~91~~84. Eckblad JW, Volden CS, Weilgart LS. Allochthonous drift from backwaters to the main channel of the Mississippi River. The American Midland Naturalist. 1984;111(1):16-22.

~~92~~85. Sheaffer WA, Nickum JG. Relative abundance of macroinvertebrates found in habitats associated with backwater area confluences in Pool 13 of the Upper Mississippi River. Hydrobiologia. 1986;136:113-20.

~~93~~86. Wahl DH, Goodrich J, Nannini MA, Dettmers JM, Soluk DA. Exploring riverine zooplankton in three habitats of the Illinois River ecosystem: Where do they come from? Limnology and Oceanography. 2008;53(6):2583-93.

~~94~~87. Burdis RM, Hoxmeier RJH. Seasonal zooplankton dynamics in main channel and backwater habitats of the Upper Mississippi River. Hydrobiologia. 2011;667:69-87.

~~95~~88. Ochs CA, Pongruktham O, Zimba PV. Darkness at the break of noon: Phytoplankton production in the Lower Mississippi River. Limnology and Oceanography. 2013;58(2):555-68.

~~96~~89. Sheehan RJ, Lewis WM, Bodensteiner LR. Winter habitat requirements and overwintering of riverine fishes. Online: http://opensiuc.lib.siu.edu/fiaq_reports/8: Southern Illinois University, 1994.

~~97~~90. Raibley PT, Irons KS, O'Hara TM, Blodgett KD, Sparks RE. Winter habitats used by largemouth bass in the Illinois River, a large river-floodplain ecosystem. North American Journal of Fisheries Management. 1997;17(2):401-12.

~~98~~91. Hurley KL, Sheehan RJ, Heidinger RC, Wills PS, Clevenstine B. Habitat use by Middle Mississippi River pallid sturgeon. Transactions of the American Fisheries Society. 2004;133:1033-41.

~~99~~92. Schwartz JS, Herricks EE. Fish use of stage-specific fluvial habitats as refuge patches during a flood in a low-gradient Illinois stream. Canadian Journal of Fisheries and Aquatic Sciences. 2005;62:1540-52.

~~100~~93. Junk WJ, Bayley PB, Sparks RE. The flood pulse concept in river-floodplain systems. Canadian special publication of fisheries and aquatic sciences. 1989;106(1):110-27.

~~101~~94. Sparks RE. Need for ecosystem management of large rivers and their floodplains. Bioscience. 1995;45(3):168-82. PubMed PMID: WOS:A1995QH75600010.

~~102~~95. Melis TS, Walters CJ, Korman J. Surprise and Opportunity for Learning in Grand Canyon: the Glen Canyon Dam Adaptive Management Program. Ecol Soc. 2015;20(3). doi: Artn 2210.5751/Es-07621-200322. PubMed PMID: WOS:000362913100013.

~~103~~96. Millar RJ, Fuglestvedt JS, Friedlingstein P, Rogelj J, Grubb MJ, Matthews HD, et al. Emission budgets and pathways consistent with limiting warming to 1.5 C. Nature Geoscience. 2017;10(10):741-7.

~~104~~97. Shkolnik I, Pavlova T, Efimov S, Zhuravlev S. Future changes in peak river flows across northern Eurasia as inferred from an ensemble of regional climate projections under the IPCC RCP8. 5 scenario. Climate dynamics. 2018;50:215-30.

~~105~~98. Fonseca AR, Santos JA. Predicting hydrologic flows under climate change: The Tâmega Basin as an analog for the Mediterranean region. Science of the total environment. 2019;668:1013-24.

~~106~~99. Jacobson RB, Annis ML, Colvin ME, James DA, Welker TL, Parsley MJ. Missouri River *Scaphirhynchus albus* (pallid sturgeon) effects analysis—Integrative report 2016. U.S. Geological Survey Scientific Investigations Report 2016-5064. Reston, VA.2016. p. 154.

~~107~~100. Hall LS, Krausman PR, Morrison ML. The habitat concept and a plea for standard terminology. Wildlife Soc B. 1997;25(1):173-82. PubMed PMID: WOS:A1997XA82200031.

~~108~~101. Fisher WL, Bozek MA, Vokoun JC, Jacobson RB. Freshwater aquatic habitat measurements. In: A. V. Zale, D. L. Parrish, Sutton T, editors. Fisheries Techniques, 3rd edition. Bethesda, Maryland: American Fisheries Society; 2012. p. 101-61.

~~109~~102. Lamouroux N, Gore JA, Lepori F, Statzner B. The ecological restoration of large rivers needs science-based, predictive tools meeting public expectations: an overview of the Rhone project. Freshwater ~~Biology~~Biol. 2015;60(6):1069-84. doi: 10.1111/fwb.12553. PubMed PMID: WOS:000354254800001.

~~110~~103. Schloesser JT, Paukert CP, Doyle WJ, Hill TD, Steffensene KD, Travnichek VH. Fish Assemblages at Engineered and Natural Channel Structures in the Lower Missouri River: Implications for Modified Dike Structures. River Res Appl. 2012;28(10):1695-707. doi: 10.1002/rra.1578. PubMed PMID: WOS:000312651000008.

~~111~~104. Jacobson RB, Berkley J. Conceptualizing and communicating ecological river restoration. In: Simon A, Bennet S, Castro J, editors. Stream Restoration in Dynamic Fluvial Systems: Scientific Approaches, Analyses and Tools: AGU Geophysical Monograph 194; 2011. p. 9-28.

105. Jacobson RB, Galat DL. Design of a naturalized flow regime-an example from the Lower Missouri River, USA. Ecohydrology: Ecosystems, Land and Water Process Interactions, Ecohydrogeomorphology. 2008;1(2):81-104. PubMed PMID: WOS:000260000500001.

106. Rudnick DT, Ortner PB, Browder JA, Davis SM. A conceptual ecological model of Florida Bay. Wetlands Ecol Manage. 2005;25:870-83.

107. Conroy MJ, Peterson JT. Decision making in natural resources—A structured, adaptive approach. Oxford, United Kingdom: Wiley-Blackwell; 2013.

108. Gregory R, Failing L, Harstone M, Long G, McDaniels T, Ohlson D. Structured decision making—A practical guide to environmental management choices. Chichester, West Sussex, United Kingdom: John Wiley & Sons; 2012.

109. Irwin ER, Freeman M, Peterson J, Kennedy KDM, Lloyd MC, Ouellette Coffman KM, et al. Adaptive management of flows from R.L. Harris Dam (Tallapoosa River, Alabama)—

Stakeholder process and use of biological monitoring data for decision making: U.S. Geological Survey Open-File Report 2019–1026. Reston, VA: U.S. Geological Survey; 2019. p. 93.

110. Martin J, Runge MC, Nichols JD, Lubow BC, Kendall WL. Structured decision making as a conceptual framework to identify thresholds for conservation and management. Ecol Appl. 2009;19(5):1079-90.

111. Chan TU, Hart BT, Kennard MJ, Pusey BJ, Shenton W, Douglas MM, et al. Bayesian network models for environmental flow decision making in the Daly River, Northern Territory, Australia. River Res Appl. 2012;28(3):283-301.

112. Williams BK, Szaro RC, Shapiro CD. Adaptive management—The U.S. Department of the Interior technical guide. In: Interior USDot, editor. Washington, D.C.2007. p. 72.

113. McGowan CP, Smith DR, Nichols JD, Lyons JE, Sweka J, Kalasz K, et al. Implementation of a framework for multi-species, multi-objective adaptive management in Delaware Bay. Biol Conserv. 2015;191:759-69.

114. Walters C, Korman J, Stevens LE, Gold B. Ecosystem modeling for evaluation of adaptive management policies in the Grand Canyon. Conserv Ecol. 2000;4(2):art. no.-1. PubMed PMID: WOS:000167492900002.

~~112~~115. U.S. Geological Survey. Graphical Fisheries Database Browser - Stratified Random Sampling 2015 [cited 2018 September 9]. Available from: https://umesc.usgs.gov/data_library/fisheries/graphical/fish_front.html.

Supporting Information Captions

Fig S1. Discharge (m^3/s) patterns over time in the Canadian River, Oklahoma near Canadian, TX (USGS 2019; USGS Gage 07228000).

Fig S2. Hydrograph showing pre- and post-Glen Canyon Dam closure in 1964 (dashed line) mean monthly discharge (m^3/s), which transitions from seasonally stochastic to a more homogeneous regime focusing on anthropogenic interests.

Fig S3. The spatial and temporal scales of the management goal, the scientific inferences needed to inform the management goal, and that data collection needs to occur to support the inferences for monitoring information needs identified as requiring additional data in the case study addressing Humpback Chub recruitment in the Colorado River between Glen Canyon Dam and Lake Mead, Arizona (see Table S2 for additional detail). A:Tier 2 EEC= larval Humpback Chub habitat; Stressors=habitat fragmentation, turbidity and Tier 2 EEC= Humpback Chub spawning habitat; Stressor= water temperature and Tier 3 EEC=insect production; Stressor=benthic macroinvertebrate habitat quantity and quality and Tier 3 EECs=all; Inter-tier interaction=trophic level interactions; B:Tier 3 EEC=larval Humpback Chub production; Stressor= larval Humpback chub habitat quantity and quality; C: Tier 3 EEC=Humpback Chub egg quality and production; Stressors= Humpback Chub spawning habitat quantity and quality; D:Larval Humpback Chub production; Inter-tier interaction=mortality of Humpback Chub eggs; E:Tier 3 EEC= larval Humpback Chub production; Stressor=predation by invasive species and Tier 3 EEC=Primary production; Stressor=nutrient flux; F:Tier 1 EEC= biogeochemistry/thermodynamics; Inter-tier interaction=sediment adsorption of contaminants and nutrients; G:Tier 3 EEC=Humpback Chub age-0 recruitment; Inter-tier interaction=mortality of larval Humpback Chub.

Fig S4. Proportion of total annual Columbia River discharge at The Dalles, OR occurring in the month of June from 1879 to 2015.

Fig S5. The spatial and temporal scales of the management goal, the scientific inferences needed to inform the management goal, and that data collection needs to occur to support the inferences for monitoring information needs identified as requiring additional data in the case study addressing White Sturgeon recruitment in the Columbia River (see Table S3 for additional detail). ~~S4~~A:Tier 1 EEC= biogeochemistry/thermodynamic; Stressor= altered biogeochemical regime and Tier 2 EEC= benthic macroinvertebrate habitat; Stressors=channel stability, sediment deposition, fragmentation and Tier 2 EEC=Larval White Sturgeon habitat; Stressors=habitat fragmentation sediment deposition, water temperature and Tier 2 EEC=White Sturgeon spawning habitat; Stressors=contaminants, sediment deposition and Tier 3 EEC=White Sturgeon egg quality and production; Stressor=predation by invasive species and Tier 3 EEC=White Sturgeon larvae production; Stressor=predation by invasive species and Tier 3 EEC=benthic macroinvertebrate production; Stressor=benthic macroinvertebrate habitat quantity and quality; B:Tier 1 EEC=sediment transport; Stressors=altered sediment regime, altered hydraulic regime and Tier 1 EECs=channel morphology/hydraulics, sediment transport; Stressor=altered hydraulic regime and Tier 3 EEC=primary production; Stressor=nutrient fluxes; C:Tier 1 EEC=channel morphology/hydraulics, sediment transport; Stressor=altered hydraulic regime and Tier 1 EEC = channel morphology/hydraulics, sediment transport; Inter-tier interaction=sediment transport dynamics; D:Tier 1 EEC=channel morphology/hydraulics, sediment transport; Stressor=altered hydraulic regime and Tier 1 EEC=channel morphology/hydraulics, sediment transport; Inter-tier interaction=sediment transport dynamics; E:Tier 1 EEC=biogeochemistry/thermodynamics; Inter-tier interaction=sediment adsorption of contaminants and nutrients; F: Tier 3 EEC=White

Formatted: Font color: Black

Sturgeon larvae production; Stressors=larval White Sturgeon habitat quantity and quality and Tier 3 EEC=White Sturgeon larvae production, age-0 White Sturgeon recruitment; Inter-tier interaction=mortality and White Sturgeon egg quality and production; Inter-tier interaction=predation of White Sturgeon eggs by native fish and Tier 3 EECs=White Sturgeon larvae production; Inter-tier interaction=predation of White Sturgeon larvae by native fish and Tier 3 EECs=all; Inter-tier interactions=trophic level interactions.

Fig S6. Largemouth bass (*Micropterus salmoides*) data from the Pool 13 of the Upper Mississippi River (A, B) and the La Grange Pool of the Illinois River (C, D). The two river reaches are roughly the same latitude, but the La Grange Pool is more limited in overwintering habitat. Population abundance is presented in panels A and C where each point is an individual fish cumulatively caught with standardized day time electrofishing (Ratcliff et al. 2014) in a specific year. The dashed triangle highlights 'missing' >400 mm size classes since 2000 in the La Grange Pool. Population size structure is indexed by proportional stock density (PSD) is presented in panels B and D with the dashed line showing trends in the largest size classes over time. Data and methodology were downloaded from the publicly available databases via the Upper Mississippi River Restoration's Long Term Resource Monitoring Graphical Fish Browser [112115].

Field Code Changed

Fig S7. The spatial and temporal scales of the management goal, the scientific inferences needed to inform the management goal, and that data collection needs to occur to support the inferences for monitoring information needs identified as requiring additional data in the case study addressing native fish biodiversity and habitat diversity in the Mississippi and Illinois rivers (see Table S4 for additional detail). A:Tier 1 EEC=sediment transport; Stressor=altered hydraulic regime and Tier 1 EEC=biogeochemistry/thermodynamics; Stressor=altered biogeochemical

Formatted: Font color: Black

regime and Tier 1 EEC=biogeochemistry/thermodynamics; Inter-tier interaction=sediment adsorption of contaminants and nutrients; B: Tier 2 EEC=adult native fish overwintering habitat; Stressors=water velocity, water temperature, dissolved oxygen, sediment deposition and Tier 2 EEC=juvenile native fish habitat; Stressors=water depth, water velocity, water temperature, dissolved oxygen, contaminants, sediment deposition and Tier 2 EEC=native fish spawning habitat; Stressors=water depth, water velocity, habitat fragmentation, sediment deposition, water temperature, dissolved oxygen, contaminants and Tier 3 EEC=adult and juvenile native fish recruitment; Inter-tier interaction=mortality and Tier 3 EEC=all; Stressors=invasive species and Tier 3 EEC=all; Inter-tier interaction=trophic level interactions; C:Tier 1 EEC=channel morphology/hydraulics; Inter-tier interaction=channel forming processes; D:Tier 1 EEC=channel morphology/hydraulics, sediment transport; Inter-tier interaction=sediment transport dynamics; E:Tier 3 EEC=adult native fish recruitment; Stressor=adult native fish overwintering habitat quantity and quality and Tier 3 EEC=juvenile native fish recruitment; Stressor=juvenile native fish habitat quantity and quality and Tier 3 EEC=native fish egg quality and production; Stressor=spawning habitat quantity and quality.

Table S1. Summary of information needs identified in the Conceptual Model describing factors affecting the recruitment of the Arkansas River Shiner in the South Canadian River, OK (Fig 4; this publication) by Essential Ecosystem Characteristic (EEC) Tier, EEC, and stressor or within Tier interactions and an assessment of the status of existing information that could be used to address the information needs.

Table S2. Summary of information needs identified in the Conceptual Model describing factors affecting the recruitment of the Humpback Chub in the Colorado River, Arizona (Fig 6; this publication) by Essential Ecosystem Characteristic (EEC) Tier, EEC, and stressor or inter-tier

interactions and an assessment of the status of existing information that could be used to address the information needs.

Table S3. Summary of information needs identified in the Conceptual Model describing factors affecting the recruitment of age-0 White Sturgeon in the Columbia River (Fig ~~8~~7; this publication), by Essential Ecosystem Characteristic (EEC) Tier, EEC, and stressor or inter-tier interactions and an assessment of the status of existing information that could be used to address the information needs.

Table S4. Summary of information needs identified in the Conceptual Model describing factors affecting the restoration and maintenance of native fish biodiversity and habitat quantity and quality in the Upper Mississippi and Illinois rivers (Fig ~~10~~8; this publication), by Essential Ecosystem Characteristic (EEC) Tier, EEC, and stressor or inter-tier interactions and an assessment of the status of existing information that could be used to address the information needs.

PONE-D-21-12444
Identifying monitoring information needs that support the management of fish in large rivers
PLOS ONE

Reviewer 1 wonders about the utility of such conceptual models in the more realistic case of multiple species contexts. How could the model be expanded in this regard, besides the case of simple richness measures? In addition, how could the model help in actually prioritising or ranking the variables or interactions identified? Finally, reviewer 1 also noticed that an important stressor related to fragmentation and connectivity is only marginally discussed and included in the model. This is critical for meta-population dynamics and should be given more emphasis.

Reviewer 2, similarly raises the critical issue of the multi-species context, and how the needs of different species could be simultaneously identified. Therefore, I advice to expand the Discussion in this regard, eventually acknowledging limitations and suggesting future research needs. Reviewer 2 also wonders how such models could effectively guide restoration and decision-making beyond monitoring needs; if the relative importance of data gaps and interactions is not quantified (e.g. via a cost-benefit analysis), how could it help prioritise the focus of monitoring and action?

Besides carefully responding to each reviewers' comments and modify the manuscript accordingly, I also suggest to simplify the manuscript, which feels rather long. Perhaps some of the background information from each case study could be included as supplementary or shortened. Also, caption from Fig.5 (and sister-figures) is rather hard to digest for the reader. I wonder if this could be simplified as well.

> Response: Thank you for the opportunity to revise our manuscript. We have tried to address comments of Reviewer 1 and 2 below and in the revised manuscript. To shorten and simplify the manuscript, we relocated some of the contextual information from the case studies and moved it to an Appendix in Supplemental Information. With respect to Fig 5 and sister figures, we have discussed trying to simplify the figure captions but have not come up with a good solution. There is a lot of information contained within the figures and feel that further generalizations would not be clarifying. We do, however, acknowledge that the figure caption format is awkwardly long. What we propose is

that we retain Fig 5 in the main body of the text as an example, and then move subsequent sister figures to Supplemental Materials. Please let us know if this satisfies your and the reviewer's comments to reduce the length of the manuscript.

Comments to the Author

5. Review Comments to the Author

Reviewer #1: In this study a conceptual model is used to aid the development of best practices of large river monitoring programs. The model was developed based on former scientific works and during scientist's workshop negotiations. Case study applications prove that the application of this complex conceptual model can be useful to identify critical information gaps, which can then be used to develop management and monitoring objectives.
I like the approach of developing such conceptual models, which can reveal information gaps, and think that the model in general can be useful to adopt across large river systems with some refinements and local adaptations. Consequently, I believe showing such an approach can provide useful information for the readers.

1) What I lack is to show more convincingly how such complex models can be used for multispecies systems, where not only the requirements of a single species is evaluated, which in fact the more realistic situation. How can individual species level models be put together to provide meaningful information for management? It would be useful to discuss this in more detail in the Discussion section.

    Response: Thank you for this comment. We have added text in the discussion that describes how the CM could contribute to our understanding of the need for a multi-species context in management activities. Please see: L645-691

2) Also a critical issue which should be briefly discussed is how the identified critical target variables should be prioritized, especially in a multispecies systems, where several variables will appear. Development of this section could convince the reader and could clearly show the applicability of such conceptual models by management.

Response: Thank you for this insight. We have provided language in the discussion that describes how the CM could be used to identify critical target variables in a multispecies context. Please see: L650-668

3) Although channel morphology/hydraulics may contain fragmentation/connectivity issues this should be made more clear in the material, because this is one of the most critical issue, which determine fish (meta)population or metacommunity dynamics. In fact fragmentation is often used as one of the most critical variable of anthropogenic drivers and as such is a critically important target to mitigate by management. However, it does not appear either in Fig 2 or Fig 3, but only on the case study figures belonging to morphology/hydraulics TIER1 components.

   Response: Thank you. We agree with your assessment. We have further emphasized the importance of fragmentation by specifically mentioning it in the manuscript section describing the CM form. Please see: L223-234. Also, in Table 2, habitat fragmentation is emphasized as affecting multiple facets of the CM in multiple river systems and is mentioned in the text describing Table 2. This result indicates the need to better study the effects of habitat fragmentation on multiple biotic components.

Reviewer #2: Dear Editor,
This study demonstrates how a conceptual model can be used to identifying knowledge gaps in the mechanisms by which Essential Ecosystem Characteristics influence large-river fish species in the USA. These gaps should then be filled to improve the effectiveness of restoration and management.
I agree on the value of these conceptual models to identify knowledge gaps and inform decisions on what to monitor to fill them and allow a better understanding of the system and, therefore, enhance our capacity to manage them adequately. However, I disagree with some of the arguments:

1) The conceptual model represents potential interactions across different structural element of the river system but does not allow quantitative evaluations of strength of those interaction. As such, the value of is conceptual model is limited to identifying knowledge gaps and cannot be used to evaluate the relative importance of each interaction. Therefore, this conceptual model should only be used for identifying knowledge gaps and not for decision-making, as argued (see L771-773), beyond monitoring.

Response: This is an excellent point and we have removed the statement in L771-773 and elaborated on the considerations that need to be accounted for, and the difficulties with, assessing benefit:cost ratios. Please see: L692-726. Also, you are correct that the CM does not provide quantitative evaluations of the strength of the relations. We do acknowledge this and suggest that the CM could provide a basis for developing quantitative assessments in L749-754 and have added language that describes how the CMs could be the basis for Structural Decision Making and Adaptive Management processes (see L727-741).

2) The conceptual model lacks a cost analysis to evaluate the most efficient way of filling knowledge gaps. Some of the gaps might be more difficult/ costly or even feasible to fill. Without such analysis we can only identify the gaps but cannot prioritise where to focus monitoring on a cost-effective way and just confirm where gaps exist.

Response: Thank you for this comment. In addition to addressing the benefit:cost issue above, we have provided language in the discussion that describes how the CM could be used to identify critical target variables in a multispecies context but that there are critical uncertainties that need to be considered. See: L650-668. We agree with you about prioritizing based on cost effectiveness but respectfully suggest that the CM could provide information that would suggest where to focus monitoring effort.

3) Three of the case studies present conceptual models for individual species. While I see the value of developing these conceptual models for charismatic endangered species, I wonder how feasible/ useful it would be this method when facing management needs for many species simultaneously. One of the case studies does present a conceptual model for the full fish community, but focused on diversity, rather than individual species, so no information of particular species issues are addressed. Would it be feasible to elaborate a conceptual model that addressed all individual species needs/ issues simultaneously? This would allow identifying knowledge gaps common to multiple species simultaneously.

Response: Thank you for this comment. We have added text in the discussion that describes how the CM could contribute to our understanding of the need for a multi-species context in management activities. Please see: Please see: L645-691

4) Minor comments:
   - L304 & 369. What does ATV stand for?

      Response: Thank you for bringing this to our attention. ATV stands for all-terrain vehicle. We have removed the acronym from the revision.

   - The manuscript is quite long, especially because of the description of each case study. It would be good to present the information of these case studies in a more synthetic way (maybe on a table?).

      Response: Thank you for the comment. Per your and the Associate Editor's recommendation we have pulled out some of the contextual information from the case studies and moved the information to an Appendix in Supplemental Information. We have also moved three figures and associated captions to the supplemental information section.
