# Supplementary material for: Identifying monitoring information needs that support the management of fish in large rivers
Source: PLoS One. 2022 Apr 29;17(4):e0267113. doi: 10.1371/journal.pone.0267113 (PMC9053787; doi:10.1371/journal.pone.0267113)
Supplement: Attachment — Submitted filename: Counihan_et al. response to reviewers.docx [file pone.0267113.s013.docx]

PONE-D-21-12444
Identifying monitoring information needs that support the management of fish in large rivers
PLOS ONE

____________________________________________________________________________________

Reviewer 1 wonders about the utility of such conceptual models in the more realistic case of multiple species contexts. How could the model be expanded in this regard, besides the case of simple richness measures? In addition, how could the model help in actually prioritising or ranking the variables or interactions identified? Finally, reviewer 1 also noticed that an important stressor related to fragmentation and connectivity is only marginally discussed and included in the model. This is critical for meta-population dynamics and should be given more emphasis.

Reviewer 2, similarly raises the critical issue of the multi-species context, and how the needs of different species could be simultaneously identified. Therefore, I advice to expand the Discussion in this regard, eventually acknowledging limitations and suggesting future research needs. Reviewer 2 also wonders how such models could effectively guide restoration and decision-making beyond monitoring needs; if the relative importance of data gaps and interactions is not quantified (e.g. via a cost-benefit analysis), how could it help prioritise the focus of monitoring and action?

Besides carefully responding to each reviewers' comments and modify the manuscript accordingly, I also suggest to simplify the manuscript, which feels rather long. Perhaps some of the background information from each case study could be included as supplementary or shortened. Also, caption from Fig.5 (and sister-figures) is rather hard to digest for the reader. I wonder if this could be simplified as well.

Response: Thank you for the opportunity to revise our manuscript. We have tried to address comments of Reviewer 1 and 2 below and in the revised manuscript. To shorten and simplify the manuscript, we relocated some of the contextual information from the case studies and moved it to an Appendix in Supplemental Information. With respect to Fig 5 and sister figures, we have discussed trying to simplify the figure captions but have not come up with a good solution. There is a lot of information contained within the figures and feel that further generalizations would not be clarifying. We do, however, acknowledge that the figure caption format is awkwardly long. What we propose is that we retain Fig 5 in the main body of the text as an example, and then move subsequent sister figures to Supplemental Materials. Please let us know if this satisfies your and the reviewer’s comments to reduce the length of the manuscript.

**Comments to the Author**

5. Review Comments to the Author

Reviewer #1: In this study a conceptual model is used to aid the development of best practices of large river monitoring programs. The model was developed based on former scientific works and during scientist’s workshop negotiations. Case study applications prove that the application of this complex conceptual model can be useful to identify critical information gaps, which can then be used to develop management and monitoring objectives.
I like the approach of developing such conceptual models, which can reveal information gaps, and think that the model in general can be useful to adopt across large river systems with some refinements and local adaptations. Consequently, I believe showing such an approach can provide useful information for the readers.

1) What I lack is to show more convincingly how such complex models can be used for multispecies systems, where not only the requirements of a single species is evaluated, which in fact the more realistic situation. How can individual species level models be put together to provide meaningful information for management? It would be useful to discuss this in more detail in the Discussion section.

Response: Thank you for this comment. We have added text in the discussion that describes how the CM could contribute to our understanding of the need for a multi-species context in management activities. Please see: L645-691

2) Also a critical issue which should be briefly discussed is how the identified critical target variables should be prioritized, especially in a multispecies systems, where several variables will appear. Development of this section could convince the reader and could clearly show the applicability of such conceptual models by management.

Response: Thank you for this insight. We have provided language in the discussion that describes how the CM could be used to identify critical target variables in a multispecies context. Please see: L650-668

3) Although channel morphology/hydraulics may contain fragmentation/connectivity issues this should be made more clear in the material, because this is one of the most critical issue, which determine fish (meta)population or metacommunity dynamics. In fact fragmentation is often used as one of the most critical variable of anthropogenic drivers and as such is a critically important target to mitigate by management. However, it does not appear either in Fig 2 or Fig 3, but only on the case study figures belonging to morphology/hydraulics TIER1 components.

Response: Thank you. We agree with your assessment. We have further emphasized the importance of fragmentation by specifically mentioning it in the manuscript section describing the CM form. Please see: L223-234. Also, in Table 2, habitat fragmentation is emphasized as affecting multiple facets of the CM in multiple river systems and is mentioned in the text describing Table 2. This result indicates the need to better study the effects of habitat fragmentation on multiple biotic components.

Reviewer #2: Dear Editor,
This study demonstrates how a conceptual model can be used to identifying knowledge gaps in the mechanisms by which Essential Ecosystem Characteristics influence large-river fish species in the USA. These gaps should then be filled to improve the effectiveness of restoration and management.
I agree on the value of these conceptual models to identify knowledge gaps and inform decisions on what to monitor to fill them and allow a better understanding of the system and, therefore, enhance our capacity to manage them adequately. However, I disagree with some of the arguments:

1) The conceptual model represents potential interactions across different structural element of the river system but does not allow quantitative evaluations of strength of those interaction. As such, the value of is conceptual model is limited to identifying knowledge gaps and cannot be used to evaluate the relative importance of each interaction. Therefore, this conceptual model should only be used for identifying knowledge gaps and not for decision-making, as argued (see L771-773), beyond monitoring.

Response: This is an excellent point and we have removed the statement in L771-773 and elaborated on the considerations that need to be accounted for, and the difficulties with, assessing benefit:cost ratios. Please see: L692-726. Also, you are correct that the CM does not provide quantitative evaluations of the strength of the relations. We do acknowledge this and suggest that the CM could provide a basis for developing quantitative assessments in L749-754 and have added language that describes how the CMs could be the basis for Structural Decision Making and Adaptive Management processes (see L727-741).

2) The conceptual model lacks a cost analysis to evaluate the most efficient way of filling knowledge gaps. Some of the gaps might be more difficult/ costly or even feasible to fill. Without such analysis we can only identify the gaps but cannot prioritise where to focus monitoring on a cost-effective way and just confirm where gaps exist.

Response: Thank you for this comment. In addition to addressing the benefit:cost issue above, we have provided language in the discussion that describes how the CM could be used to identify critical target variables in a multispecies context but that there are critical uncertainties that need to be considered. See: L650-668. We agree with you about prioritizing based on cost effectiveness but respectfully suggest that the CM could provide information that would suggest where to focus monitoring effort.

3) Three of the case studies present conceptual models for individual species. While I see the value of developing these conceptual models for charismatic endangered species, I wonder how feasible/ useful it would be this method when facing management needs for many species simultaneously. One of the case studies does present a conceptual model for the full fish community, but focused on diversity, rather than individual species, so no information of particular species issues are addressed. Would it be feasible to elaborate a conceptual model that addressed all individual species needs/ issues simultaneously? This would allow identifying knowledge gaps common to multiple species simultaneously.

Response: Thank you for this comment. We have added text in the discussion that describes how the CM could contribute to our understanding of the need for a multi-species context in management activities. Please see: Please see: L645-691

4) Minor comments:
   - L304 & 369. What does ATV stand for?

Response: Thank you for bringing this to our attention. ATV stands for all-terrain vehicle. We have removed the acronym from the revision.

- The manuscript is quite long, especially because of the description of each case study. It would be good to present the information of these case studies in a more synthetic way (maybe on a table?).

Response: Thank you for the comment. Per your and the Associate Editor’s recommendation we have pulled out some of the contextual information from the case studies and moved the information to an Appendix in Supplemental Information. We have also moved three figures and associated captions to the supplemental information section.
