# Supplementary material for: Identifying monitoring information needs that support the management of fish in large rivers
Source: PLoS One. 2022 Apr 29;17(4):e0267113. doi: 10.1371/journal.pone.0267113 (PMC9053787; doi:10.1371/journal.pone.0267113)
Supplement: Attachment — Submitted filename: Response to reviewers.docx [file pone.0267113.s015.docx]

PONE-D-21-12444R1

Identifying monitoring information needs that support the management of fish in large rivers

PLOS ONE

Dear Dr. Counihan,

Thank you for submitting your manuscript to PLOS ONE. After careful consideration, we feel that it has merit but does not fully meet PLOS ONE’s publication criteria as it currently stands. Therefore, we invite you to submit a revised version of the manuscript that addresses the points raised during the review process.

==============================
Dear Dr Counihan,

first of all, I apologize for the very long processing time of you manuscript. We have now obtained comments from two additional reviewers; they were aware that the manuscript was already revised and they also had access to the original (first round) comments.

Both reviewers clearly see the value of this work. Reviewer 3 makes an important point regarding the usability of your approach beyond the specific case studies and the scientists involved. In order to facilitate the uptake of your approach to a wider audience, perhaps a step-by-step guide or a concise follow-through instruction box could really help. Reviewer 4 has only minor comments and suggests edits to aid readability of the manuscript. Please note that Reviewer 4 has included an attachment.

We will be pleased to accept this paper once you considered both reviewers comments carefully.

Looking forward to receive the last revision.

Many thanks for your patience

Response: Please see our responses to the reviewer comments below. We appreciate that specific comments were made to improve the readability of the manuscript. Per Reviewer 3’s and your suggestion we have included a series of recommended steps as a table (Table 4) and referenced that recommended steps are included in the abstract and conclusion section. Thank you for the suggestion.

==============================

Please submit your revised manuscript by Dec 25 2021 11:59PM. If you will need more time than this to complete your revisions, please reply to this message or contact the journal office at [plosone@plos.org](https://mail.google.com/mail/?view=cm&fs=1&tf=1&to=plosone@plos.org). When you're ready to submit your revision, log on to https://www.editorialmanager.com/pone/ and select the 'Submissions Needing Revision' folder to locate your manuscript file.

Please include the following items when submitting your revised manuscript:

- A rebuttal letter that responds to each point raised by the academic editor and reviewer(s). You should upload this letter as a separate file labeled 'Response to Reviewers'.
- A marked-up copy of your manuscript that highlights changes made to the original version. You should upload this as a separate file labeled 'Revised Manuscript with Track Changes'.
- An unmarked version of your revised paper without tracked changes. You should upload this as a separate file labeled 'Manuscript'.

If you would like to make changes to your financial disclosure, please include your updated statement in your cover letter. Guidelines for resubmitting your figure files are available below the reviewer comments at the end of this letter.

If applicable, we recommend that you deposit your laboratory protocols in protocols.io to enhance the reproducibility of your results. Protocols.io assigns your protocol its own identifier (DOI) so that it can be cited independently in the future. For instructions see: <https://journals.plos.org/plosone/s/submission-guidelines#loc-laboratory-protocols>. Additionally, PLOS ONE offers an option for publishing peer-reviewed Lab Protocol articles, which describe protocols hosted on protocols.io. Read more information on sharing protocols at <https://plos.org/protocols?utm_medium=editorial-email&utm_source=authorletters&utm_campaign=protocols>.

We look forward to receiving your revised manuscript.

Kind regards,

Stefano Larsen
Academic Editor
PLOS ONE

Journal Requirements:

Please review your reference list to ensure that it is complete and correct. If you have cited papers that have been retracted, please include the rationale for doing so in the manuscript text, or remove these references and replace them with relevant current references. Any changes to the reference list should be mentioned in the rebuttal letter that accompanies your revised manuscript. If you need to cite a retracted article, indicate the article’s retracted status in the References list and also include a citation and full reference for the retraction notice.

[Note: HTML markup is below. Please do not edit.]

Reviewers' comments:

Reviewer's Responses to Questions

**Comments to the Author**

1. If the authors have adequately addressed your comments raised in a previous round of review and you feel that this manuscript is now acceptable for publication, you may indicate that here to bypass the “Comments to the Author” section, enter your conflict of interest statement in the “Confidential to Editor” section, and submit your "Accept" recommendation.

Reviewer #3: (No Response)

Reviewer #4: (No Response)

2. Is the manuscript technically sound, and do the data support the conclusions?

The manuscript must describe a technically sound piece of scientific research with data that supports the conclusions. Experiments must have been conducted rigorously, with appropriate controls, replication, and sample sizes. The conclusions must be drawn appropriately based on the data presented.

Reviewer #3: Yes

Reviewer #4: Yes

3. Has the statistical analysis been performed appropriately and rigorously?

Reviewer #3: N/A

Reviewer #4: Yes

4. Have the authors made all data underlying the findings in their manuscript fully available?

The [PLOS Data policy](http://www.plosone.org/static/policies.action#sharing) requires authors to make all data underlying the findings described in their manuscript fully available without restriction, with rare exception (please refer to the Data Availability Statement in the manuscript PDF file). The data should be provided as part of the manuscript or its supporting information, or deposited to a public repository. For example, in addition to summary statistics, the data points behind means, medians and variance measures should be available. If there are restrictions on publicly sharing data—e.g. participant privacy or use of data from a third party—those must be specified.

Reviewer #3: (No Response)

Reviewer #4: Yes

5. Is the manuscript presented in an intelligible fashion and written in standard English?

PLOS ONE does not copyedit accepted manuscripts, so the language in submitted articles must be clear, correct, and unambiguous. Any typographical or grammatical errors should be corrected at revision, so please note any specific errors here.

Reviewer #3: Yes

Reviewer #4: Yes

6. Review Comments to the Author

Please use the space provided to explain your answers to the questions above. You may also include additional comments for the author, including concerns about dual publication, research ethics, or publication ethics. (Please upload your review as an attachment if it exceeds 20,000 characters)

Reviewer #3: This revised manuscript defines and explores a conceptual model setting a structured framework that aims to identify gaps in monitoring information that could inform the management of fish in large rivers. After reading the manuscript, I still have the general feeling that something is lacking to make a more useful contribution to river and fish managers. As it stands, I feel that the manuscript is the result of trying to synthetize discussions and outcomes from the held workshops. But the manuscript tries to offer more than that. If I understand well, the authors want to provide a model to be followed so that, at the end, the model user comes out with the variable(s) that would need to be measured in order to fill the knowledge gap(s) that we need to improve the management of a river or a species. If the authors want their model to be used by other researchers and managers than those that participated to the workshops and those that work on the four study cases, a clear guidance through the different steps should be provided, just as a manual. Otherwise, the manuscript is just the result of a brainstorming that the authors want to share in a paper. I am NOT saying that the information provided in the manuscript is useless or has no rigor or significance. On the contrary, I think the manuscript brings interesting evidence and synthesis. I am just saying that if the authors want their model to be used, they need to provide clear step-by-step guidance. I think this is more useful than case studies if they want the model to be largely used.
PlosOne is publishing papers on the basis of methodological rigor and high ethical standards. Based on that and since the manuscript is rather conceptual, I think the manuscript can be published in this journal without further changes, so that my above general comment is only a suggestion.

L. 114-116. Something is missing in this sentence?

Response: We have removed the sentence to clarify issues noted by Reviewer 4 (see below).

L.264. add “hierarchically” after “the spatial scales considered are”

Response: Thank you we have implemented your suggestion.

L.512-515. This is repeated in many figures. Consider reducing this to avoid repetitions

Response: Thank you, we have removed this text in the body of the manuscript to avoid redundancy but respectfully suggest that the description needs to be repeated in the figures so that they can be viewed as “stand alone” items.

Resolution of the figures must be improved

Response: Thank you, we have confirmed the resolution of our figures using the PACE tool.

Reviewer #4: In this manuscript the authors used a conceptual model to help management of large river monitoring programs in identifying knowledge gaps.
I believe the authors have addressed very well the comments from the first round of review. For this reason the manuscript story and approach sound solid with a good description for the four cases, highlighting the main potential factors of disturbance and knowledge gaps in large rivers as well as when informations are not enough and more data are necessary.
I have included in the attached pdf some small comments to help the authors to improve the readability, and I believe that the authors would not need any additional comment on the main manuscript concept.

Best regards

Response:

L35 – Thank you for the suggestion but we respectfully suggest that adding this language to the abstract may be more detracting than helpful. We understand your point though. We tried to strike a balance here between succinctness and providing detail.

L48 – Thank you, we have incorporated this suggestion

L54 – Thank you, we have incorporated this suggestion

L114, 131, 152 – We have made edits to paragraphs that encompass these comments to clarify the language about the sequencing of events and who was attending the workshop etc. Thank you for this observation.

L203 - Thank you, we have incorporated this suggestion

L227 – Thank you, we have enumerated the steps in the sentence to retain the connection of the elements and clarify the sequence.

L306 – Thank you, we are aware that the figure captions are long, but this represents our best attempt at minimizing the text while including enough information to understand the complexity in the figures.

L467 – Thank you, we respectfully suggest that the elements in the sentence need to remain together. However, we have edited the sentence to shorten it, hopefully improve the readability, and clarify the content.

L723 – Thank you, for this comment. We have edited the sentence to improve readability.

7. PLOS authors have the option to publish the peer review history of their article ([what does this mean?](https://journals.plos.org/plosone/s/editorial-and-peer-review-process#loc-peer-review-history)). If published, this will include your full peer review and any attached files.


If you choose “no”, your identity will remain anonymous but your review may still be made public.


**Do you want your identity to be public for this peer review?** For information about this choice, including consent withdrawal, please see our [Privacy Policy](https://www.plos.org/privacy-policy).

Reviewer #3: No

Reviewer #4: No

[NOTE: If reviewer comments were submitted as an attachment file, they will be attached to this email and accessible via the submission site. Please log into your account, locate the manuscript record, and check for the action link "View Attachments". If this link does not appear, there are no attachment files.]

While revising your submission, please upload your figure files to the Preflight Analysis and Conversion Engine (PACE) digital diagnostic tool, <https://pacev2.apexcovantage.com/>. PACE helps ensure that figures meet PLOS requirements. To use PACE, you must first register as a user. Registration is free. Then, login and navigate to the UPLOAD tab, where you will find detailed instructions on how to use the tool. If you encounter any issues or have any questions when using PACE, please email PLOS at [figures@plos.org](https://mail.google.com/mail/?view=cm&fs=1&tf=1&to=figures@plos.org). Please note that Supporting Information files do not need this step.
